# Smoothened and ciliary GPCRs regulate ciliary protein kinase A activity involved in Hedgehog signal transduction

**DOI:** 10.1371/journal.pbio.3003841

**Published:** 2026-06-10

**Authors:** Thi D. Nguyen, Mia J. Konjikusic, Lorenzo M. Del Castillo, Roshanak Irannejad, Jeremy F. Reiter

**Affiliations:** 1 Department of Biochemistry and Biophysics, Cardiovascular Research Institute, University of California, San Francisco, California, United States of America; 2 Chan Zuckerberg Biohub, San Francisco, California, United States of America; Rheinische Friedrich-Wilhelms-Universitat Bonn, GERMANY

## Abstract

Hedgehog (HH) signaling in vertebrates is dependent on the primary cilium, an organelle that scaffolds signal transduction. HH signals induce ciliary enrichment of Smoothened (SMO) and ciliary departure of the G protein-coupled receptor (GPCR) GPR161 to trigger GLI activation of the HH transcriptional program. Recently, SMO has been shown to inhibit protein kinase A (PKA). To test the hypothesis that SMO inhibits PKA at cilia to activate the HH signal transduction pathway, we developed a ciliary PKA reporter. Ciliary PKA activity was graded during zebrafish development. Activation of the HH signal transduction pathway by either Sonic hedgehog (SHH) or SMO agonist (SAG) inhibited ciliary PKA activity. Blocking SMO phosphorylation by GRK2/3 prevented ciliary SMO from inhibiting ciliary PKA activity. Converting the SMO C-terminal PKA pseudosubstrate site into a consensus PKA substrate blocked SMO-mediated inhibition of ciliary PKA activity. A ciliary GPCR, SSTR3, activated ciliary PKA and induced HH transcriptional responses in NIH/3T3 cells via a different mechanism: activation of Gα_i/o_. A different ciliary GPCR, GPR161, possesses an A-Kinase Anchoring Protein (AKAP), which we found was critical for the ciliary localization of the catalytic subunit of PKA (PKA-C) to promote ciliary PKA activity. We propose that HH signal transduction is inhibited by GPR161-mediated ciliary enrichment of PKA-C, and activated by GRK2/3-phosphorylated SMO inhibition of ciliary PKA activity.

## Abstract 2

Hedgehog signaling in vertebrates is transduced by the transmembrane protein Smoothened (SMO) on the primary cilium, but the underlying mechanisms remain unclear. This study shows that SMO and ciliary GPCRs regulate ciliary PKA activity to activate the Hedgehog pathway.

## Introduction

The Hedgehog (HH) signaling pathway is a critical means of cell-cell communication used by metazoans to coordinate development and homeostasis of many tissues [1,2]. Indispensable to vertebrate HH signaling is the primary cilium, a microtubule-based organelle that projects from the body of the cell [3–5]. Though the ciliary membrane is contiguous with the plasma membrane and the cilioplasm is not membrane-bounded, the composition of the primary cilium is distinct from that of the rest of the cell [6–11].

In the absence of HH signals, the HH receptor, PTCH1, localizes to the ciliary membrane and keeps the downstream signal transduction pathway off [12]. In the presence of HH signals, HH binds to PTCH1, allowing the seven-pass transmembrane protein Smoothened (SMO) to accumulate in the ciliary membrane [13]. Ciliary SMO is required for activation of GLI transcription factors, the HH effectors in many tissues [14,15]. How SMO activates GLI transcription factors remains an area of active investigation.

One possible mechanism by which SMO regulates GLI transcription factors is via protein kinase A (PKA). PKA represses HH signal transduction by phosphorylating GLI proteins to trigger the formation of their repressor forms, referred to as GLI-R [15–17]. The PKA holoenzyme is comprised of a catalytic kinase subunit (PKA-C) and an inhibitory regulatory subunit (PKA-R) [18]. PKA is activated by PKA-R binding to cyclic adenosine monophosphate (cAMP), a second messenger mediating some forms of GPCR signaling, thereby releasing PKA-C to phosphorylate its substrates [19].

Many GPCRs signal by acting as guanine nucleotide exchange factors for trimeric GTPases, including Gα_s_, Gα_i_, and Gα_o_ [20]. Once GTP-bound, these Gα proteins regulate the activity of adenylyl cyclases, enzymes that generate cAMP [21]. Adenylyl cyclases are stimulated by Gα_s_ and inhibited by Gα_i/o_ [22]. Gα_i/o_ has been investigated as an effector of SMO in HH signal transduction [23–29].

Beyond cAMP, some proteins, known as protein kinase inhibitors (PKIs), regulate PKA activity. PKIs inhibit PKA-C by directly binding to its active site as a pseudosubstrate [30]. Recent work has demonstrated that the C-terminus of SMO can bind PKA and function as a PKI [29,31].

Another regulator of HH signal transduction is GPR161, a Gα_s_-coupled GPCR that localizes to the primary cilium in the absence of HH signals and exits the cilium after SMO is activated and accumulates in the cilium [32–35]. A model of HH signal transduction is that GPR161, signaling through Gα_s_, activates adenylyl cyclases to increase levels of cAMP, activating PKA, and thereby triggers the formation of GLI-R [32]. However, emerging data suggest that the ability of GPR161 to stimulate Gα_s_ may be dispensable for its inhibition of HH signal transduction [36].

Previously, we found that a pool of PKA localizes to the cilium and that inhibition of ciliary PKA, but not nonciliary PKA, is sufficient to activate HH-dependent transcription [37]. Inspired by recent discoveries that SMO inhibits PKA and that HH signaling causes PKA to leave cilia along with GPR161 [29,31,38], we hypothesized that SMO activates HH signaling by specifically inhibiting PKA at cilia. To begin to test this hypothesis, we developed a sensitive measure of ciliary PKA activity. We used this assay to investigate whether SMO inhibits ciliary PKA, whether Gα_i/o_ inhibits ciliary PKA, whether SMO regulates ciliary PKA through Gα_i/o_ or through direct inhibition, and whether GPR161 regulates ciliary PKA activity.

## Results

### Development of a reporter of ciliary PKA activity

**Fig 1:**
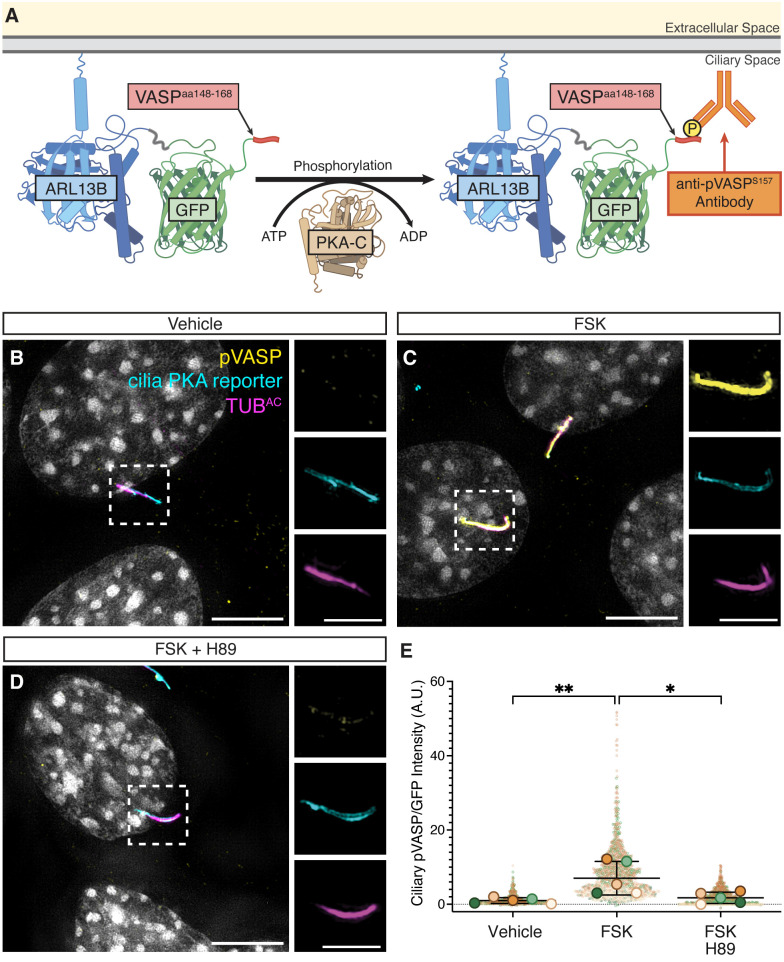
Cilia PKA reporter detects ciliary PKA activity. **(A)** Schematic of ARL13B-GFP-VASP^148-163^, the cilia PKA biosensor. **(B–D)** Immunofluorescence imaging of NIH/3T3 cells stably expressing the cilia PKA reporter. Cells were serum-starved and then treated with either vehicle **(B)**, FSK (100 nM for 15 min) **(C)**, or both FSK and H89 (100 nM and 20 µM, respectively, for 15 min) **(D)**. Images depict cells immunostained for pVASP (pVASP^S157^, yellow), cilia PKA reporter (GFP, cyan), cilia (acetylated tubulin, TUB^AC^, magenta), and nuclei (Hoechst, gray). Scale bars for larger images are 5 µm, and for insets are 2.5 µm. **(E)** Quantification of ciliary pVASP intensity normalized to ciliary GFP intensity. Representative images used for quantification are in S1A–S1C Fig. Each biological replicate is color-coded. Significance was determined via one-way ANOVA of the means of each biological replicate, followed by Šídák’s multiple comparison test. (*P* values are indicated as follows: *p < 0.04, **p < 0.003. Data are represented as means of replicates ± SD.) The underlying data for this figure are in S1 Data.

A previously developed reporter of cytosolic PKA activity [39,40] is based on vasodilator-stimulated phosphoprotein (VASP) Ser^157^, which is phosphorylated specifically by PKA [41–44]. To localize this PKA-phosphorylated peptide at cilia, we fused VASP amino acids 148–164 to ARL13B, a ciliary protein, and GFP (Fig 1A). Stable expression of ARL13B-GFP-VASP^148-163^ in a clonal NIH/3T3 cell line revealed that it, as predicted, localized to cilia (Fig 1B–1D).

To detect PKA-mediated phosphorylation of the VASP peptide, we employed a previously characterized monoclonal antibody that specifically recognizes VASP phosphorylated at Ser^157^ (pVASP) [39]. To test whether PKA can phosphorylate cilia-localized VASP peptide, we treated ciliary VASP-expressing cells with the adenylyl cyclase agonist forskolin (FSK), which induces cAMP production [45]. Immunofluorescence imaging of ciliary VASP-expressing cells using the pVASP-specific antibody revealed that, in the absence of FSK, ciliary pVASP was low and, in the presence of FSK, ciliary pVASP was high (Figs 1C, 1E, S1A, S1B, S1D, S1E). Inhibiting PKA with H89 blocked the FSK-induced increase of ciliary pVASP (Figs 1D, 1E, S1C, S1D). We conclude that this assay measures ciliary PKA activity, and we refer to ARL13B-GFP-VASP^148-163^ hereafter as the cilia PKA reporter.

We tested the dynamic range of the cilia PKA reporter in response to different concentrations of FSK and different durations of FSK treatment. FSK activated the cilia PKA reporter in both dose- and time-dependent ways (S1A–S1E Fig). Treatment of cells with 100 nM of FSK for 15 min produced an intermediate increase in cilia PKA reporter activity.

### Ciliary PKA activity is graded during zebrafish development

During zebrafish development, SHH produced by the notochord pattern the somites and induce muscle pioneers and slow muscle fibers [46]. Because somites are generated in an anterior to posterior manner, at a given developmental time point, the anterior somites can be more mature, whereas posterior somites can still be differentiating and actively responding to SHH [47,48].

**Fig 2:**
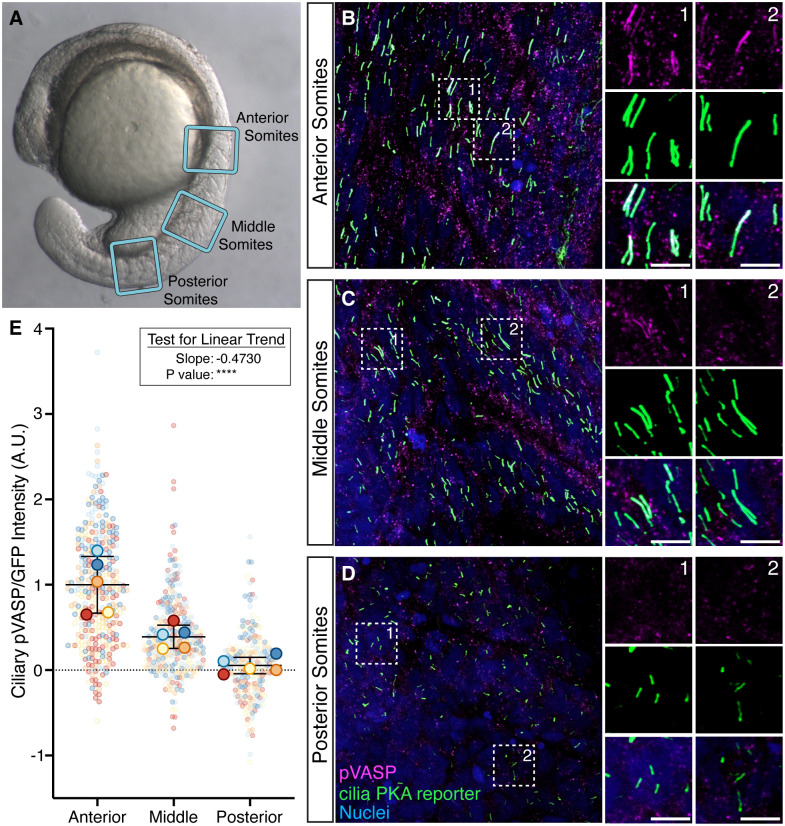
Ciliary PKA activity is graded from anterior to posterior somites in zebrafish development. **(A)** 18 somite-stage zebrafish embryo, somites 2–4 of which are designated anterior, somites 7–9 of which are designated middle, and somites 12–14 of which are designated posterior. **(B–D)** Immunofluorescence images of somites from zebrafish injected with 500 pg mRNA encoding cilia PKA reporter and stained for pVASP (pVASP^S157^, magenta), cilia PKA reporter (GFP, green), and nuclei (Hoechst, blue). Scale bar, 10 µm. **(E)** Quantification of ciliary pVASP intensity normalized to ciliary GFP within each somite region. For all plots, each biological replicate, one fish, is color-coded. Significance was determined via one-way ANOVA of the means of each biological replicate, followed by a post-test for linear trend. (****p < 0.0001. Data are represented as means of replicates ± SD.) The underlying data for this figure are in S2 Data.

Therefore, we hypothesized that, if ciliary PKA activity is suppressed by HH signaling, zebrafish somites would exhibit a posterior to anterior gradient of ciliary PKA activity. To test this hypothesis, we expressed the cilia PKA reporter in zebrafish embryos and stained for pVASP and GFP (Fig 2A–2D). Cilia PKA reporter activity was higher in anterior somites and lower in posterior somites (Fig 2E). Thus, in vivo, ciliary PKA active is dynamic, and domains of active HH signaling display decreased ciliary PKA.

### Active SMO suppresses ciliary PKA activity

**Fig 3:**
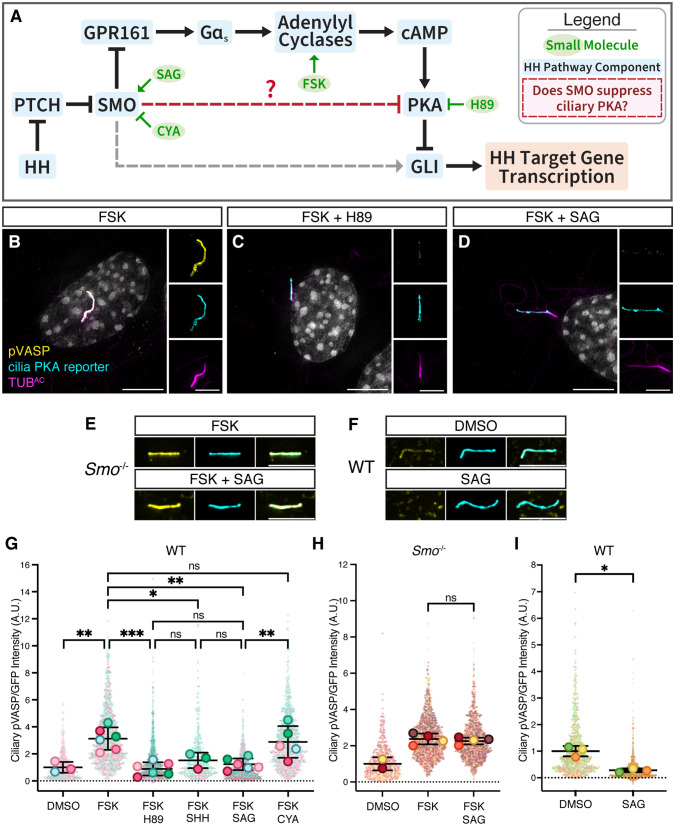
Active SMO inhibits PKA activity at cilia. **(A)** Schematic of a working model of HH signal transduction. The red line indicates part of the system investigated in this figure. **(B–F)** Immunofluorescence images of cilia PKA reporter cells or *Smo*^−/−^ cilia PKA reporter cells serum-starved and treated with either FSK (100 nM for 15 min) (B), FSK and H89 (100 nM and 20 µM, respectively, for 15 min) (C), SAG and FSK (100 nM for 24 h and 100 nM for 15 min, respectively) (D, E), or SAG alone (100 nM for 24 h). Images depict cells stained for pVASP (pVASP^S157^, yellow), cilia PKA reporter (GFP, cyan), cilia (TUB^AC^, magenta), and nuclei (Hoechst, gray). Scale bars for larger images, 5 µm (B–E). Scale bars for insets are 2.5 µm (B–D). **(G)** Quantification of ciliary pVASP intensity in cilia PKA reporter cells. Cells in the FSK and SHH condition were treated with 24 h of 4 nM SHH followed by 100 nM FSK for 15 min. Cells in the FSK and CYA condition were treated with 24 h of 5 µM cyclopamine followed by 100 nM FSK for 15 min. Some datapoints for this panel are part of a dataset also used in Fig 4. **(H)** As with G, but with quantification of ciliary pVASP intensity in *Smo*^−/−^ cilia PKA reporter cells. Data for this panel is also used in Figs 6B and S6A. **(I)** Quantification of ciliary pVASP intensity in cilia PKA reporter cells. Cells in the SAG condition were treated with 100 nM SAG for 24 h. Data for this panel are also used in Fig 5D. For all plots, each biological replicate is color-coded. Significance was determined via one-way ANOVA of the means of each biological replicate, followed by Šídák’s multiple comparison test. *P* values are indicated as follows: *p < 0.04, **p < 0.003, and ***p < 0.0002. Data are represented as means of replicates ± SD. The underlying data for this figure are in S3 Data.

As SMO can inhibit PKA [31], we hypothesized that SMO inhibits PKA at the cilium (Fig 3A). Assays for PKA inhibition, such as with Gα_i/o_-coupled GPCRs, are often conducted under conditions of cAMP stimulation, such as with FSK [45,49]. Therefore, to test whether SMO inhibits ciliary PKA, we treated cilia PKA reporter-expressing NIH/3T3 cells with FSK, FSK and H89, FSK and Smoothened Agonist (SAG), or SAG alone [50,51] (Fig 3B–3I). SAG induced ciliary accumulation of SMO and, like H89, blocked FSK-mediated activation of the cilia PKA reporter (Figs 3C, 3D, 3G, S2B, S2C, and S2F). SHH, like SAG, blocked FSK-mediated activation of the cilia PKA reporter (Fig 3G). We further tested whether activated ciliary SMO would inhibit the cilia PKA reporter even in the absence of cAMP stimulation. Indeed, SAG also decreased cilia PKA reporter activity in the absence of FSK (Fig 3F, 3I). We conclude that activating HH signal transduction attenuates PKA activity in the primary cilium.

To test if HH pathway-mediated inhibition of ciliary PKA is mediated by SMO, we used clustered regularly interspaced short palindromic repeats (CRISPR)–mediated editing to inactivate *Smo* in NIH/3T3 cells (S2A Fig). We treated clonal *Smo*^−/−^ NIH/3T3 cells expressing the cilia PKA reporter with FSK and SAG. In *Smo*^−/−^ cells, SAG had no effect on cilia PKA reporter activity (Fig 3E, 3H), indicating that SMO is critical for suppressing ciliary PKA activity in response to HH pathway activation.

Cyclopamine (CYA) is a small molecule inhibitor of SMO that triggers accumulation of SMO at the primary cilium but keeps SMO in an inactive conformation [14,50,52–54]. Treatment of cilia PKA reporter-expressing NIH/3T3 cells with CYA, accordingly, caused SMO to accumulate at primary cilia, at levels similar to that caused by treatment with SAG (S2B–S2F Fig). We used CYA to test whether ciliary localization of inactive SMO affected ciliary PKA activity. Unlike SAG, CYA did not prevent FSK-mediated activation of the cilia PKA reporter (Fig 3D, 3F). Thus, SMO localization to the primary cilium is not sufficient to inhibit ciliary PKA; SMO must also be in an active state.

### GRK2/3 phosphorylation of SMO is required to suppress PKA activity at the cilium

G protein-coupled receptor kinases 2 and 3 (GRK2/3) promote vertebrate HH signal transduction, and how they regulate HH signal transduction is being actively investigated [5,29,31,34,55–57]. For many activated GPCRs, GRKs participate in desensitization [58]. Indeed, phosphorylation of GPR161 by GRK2 triggers β-Arrestin recruitment and trafficking of GPR161 out of the cilium [33]. However, GRK2/3 has GPR161-independent functions in HH signaling, as GRK2/3 is required for activation of HH target gene transcription even in the absence of GPR161 [34].

**Fig 4:**
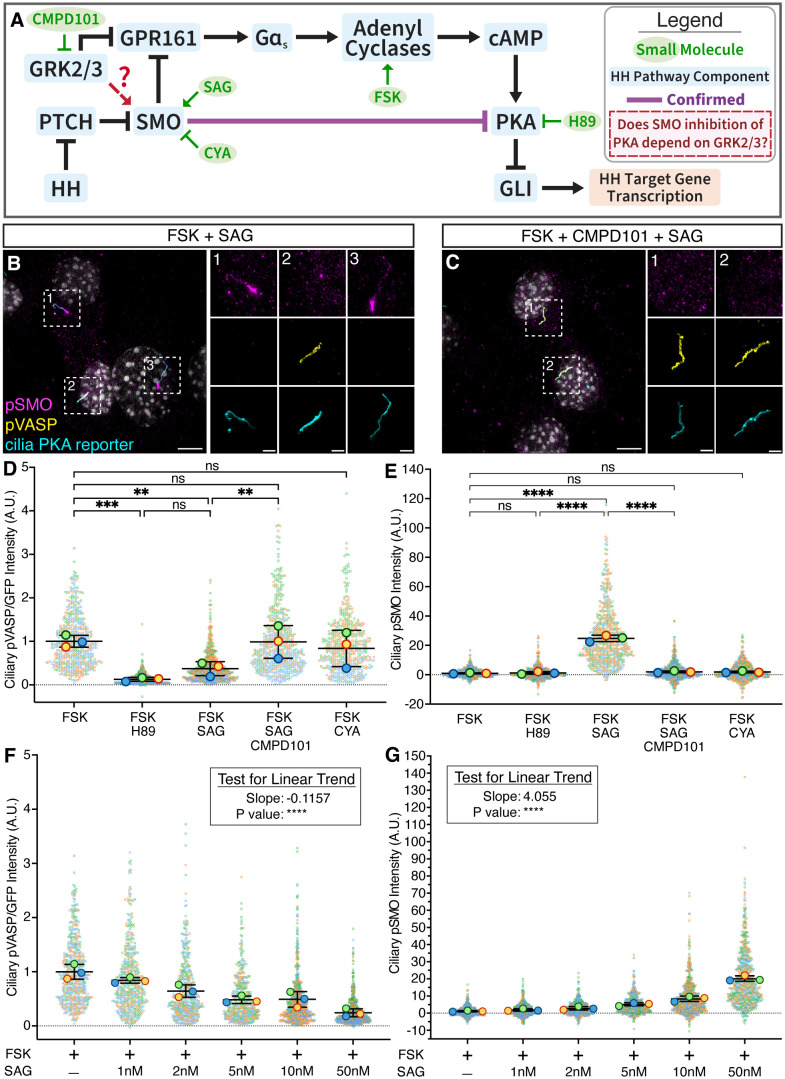
GRK2/3 activity is required for SMO to suppress ciliary PKA activity. **(A)** Schematic of the working model of HH signaling. The red arrow indicates part of the system investigated in this figure. **(B** and **C)** Immunofluorescence images of cilia PKA reporter cells. Cells were treated with SAG and FSK (100 nM for 24 h and 100 nM for 15 min, respectively) (B), or SAG, CMPD101 and FSK (100 nM, 30 µM and 100 nM for 24 h, 24 h and 15 min, respectively) (C). Images depict cells stained for pVASP (pVASP^S157^, yellow), cilia PKA reporter (GFP, cyan), pSMO (phospho-SMO S362/S363/S364, magenta), and nuclei (Hoechst, gray). Scale bars for larger images are 5 µm, and for insets are 2.5 µm. **(D)** Quantification of ciliary pVASP intensity in cilia PKA reporter cells stained for pVASP. Cells were treated with SAG (100 nM), SAG and CMPD101 (100 nM and 30 µM, respectively), CYA (5 µM) or H89 (20 µM for 15 min), and FSK (100 nM for 15 min). **(E)** As with D, but for the quantification of ciliary pSMO intensity in the same cells. **(F)** Quantification of ciliary pVASP intensity in cilia PKA reporter cells. Cells were treated with increasing dosages of SAG (for 24 h) and FSK (75 nM for 15 min). Significance was determined by a one-way ANOVA followed by a post-test for linear trend. **(G)** As with F, but for the quantification of ciliary pSMO intensity in the same cells. For all plots, each biological replicate is color-coded. For D and E, significance was determined via one-way ANOVA of the means of each biological replicate, followed by Šídák’s multiple comparison test (panels D and E), or by a post-test for linear trend (panels F and G). *P* values are indicated as follows: **p < 0.003, ***p < 0.0002, and ****p < 0.0001. Data are represented as means of replicates ± SD. The underlying data for this figure are in S4 Data.

GRK2/3 is also able to phosphorylate the C-terminal tail of SMO [29,55,59,60], and GRK2/3-phosphorylated SMO is enriched in the primary cilium following HH activation [60]. One possibility is that GRK2/3 mediates the interaction between SMO and PKA by phosphorylating the C-terminal tail of ciliary SMO, allowing SMO to directly bind and inhibit the catalytic subunit of PKA (PKA-C) [29,31,60] (Fig 4A). Therefore, we assessed whether SMO-mediated inhibition of ciliary PKA depends on GRK2/3 activity.

To test whether GRK2/3 acts at the level of SMO to suppress ciliary PKA during HH signaling, we employed CMPD101, a pharmacological inhibitor of GRK2/3 [61]. Consistent with previous findings [60], activating SMO with SAG increased ciliary SMO phosphorylated at a GRK2/3 consensus site and CMPD101 blocked GRK2/3-dependent phosphorylation of SMO (Fig 4B, 4C). Treatment of cilia PKA reporter-expressing NIH/3T3 cells with FSK, SAG and CMPD101 revealed that CMPD101 blocked the ability of SMO to inhibit PKA at the cilium (Fig 4B–4E). CYA treatment of cilia PKA reporter cells blocked GRK2/3-dependent phosphorylation of SMO (Fig 4E). Thus, GRK2/3 phosphorylation of active ciliary SMO is required to inhibit ciliary PKA.

To further assess how SMO activation affects its phosphorylation and ability to inhibit ciliary PKA, we measured the SAG dose response of cilia PKA reporter activity and ciliary phosphorylated SMO levels. SAG increased SMO phosphorylation and decreased cilia PKA reporter activity in a dose-dependent way (Fig 4F, 4G).

Interestingly, immunofluorescence imaging of GRK2/3 phosphorylated SMO (pSMO) and pVASP in cells treated with intermediate levels of SAG revealed that cells were heterogeneous, exhibiting ciliary pVASP or pSMO, but not high levels of both (S3 Fig). Quantification revealed that ciliary SMO phosphorylation and ciliary PKA activity were anti-correlated both at the population level and the single cell level. We conclude that GRK2/3 phosphorylation of SMO is critical for inhibition of ciliary PKA and that the activities of ciliary GRK2/3 and PKA are negatively correlated.

### Gα_i_ inhibits ciliary PKA activity downstream of a ciliary GPCR, but not downstream of SMO

**Fig 5:**
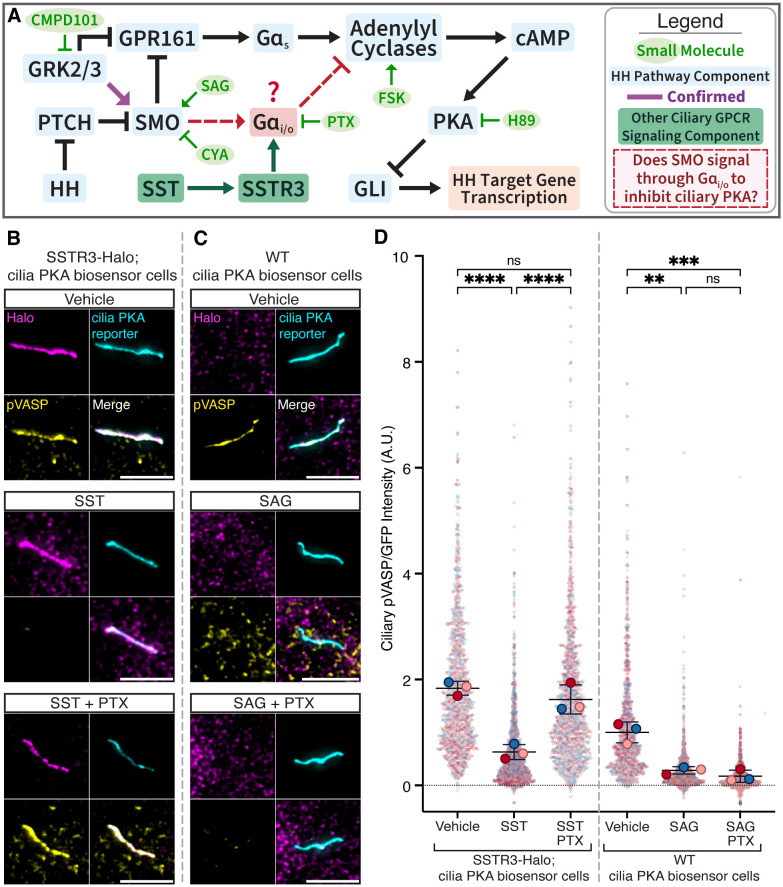
Gα_i_ inhibits ciliary PKA, but does not mediate inhibition of ciliary PKA by SMO. **(A)** Schematic of the working model of HH signaling. Red arrows indicate areas of inquiry relevant to this figure. **(B)** Immunofluorescence imaging of cilia PKA reporter cells stably expressing Halo-tagged SSTR3 in response to doxycycline. Cells were serum-starved and treated with either vehicle, SST (10 µM for 2 h), or SST and PTX (10 µM for 2 h and 100 ng/mL for 16 h, respectively). Images depict cells stained for pVASP (pVASP^S157^, yellow), cilia PKA reporter (GFP, cyan), and Halo (magenta). To detect basal levels of pVASP without FSK treatment in this experiment, we used a higher laser power than in previously described experiments. Scale bars are 5 µm. **(C)** Immunofluorescence imaging of cilia PKA reporter cells. Cells were serum-starved and treated with either vehicle, SAG (100 nM for 24 h), or SAG and PTX (100 nM for 24 h and 100 ng/mL for 16 h, respectively). **(D)** Quantification of ciliary pVASP intensity, normalized to ciliary GFP, of C and D. Each biological replicate has its own color. Significance was determined via one-way ANOVA of the means of each biological replicate, followed by Šídák’s multiple comparison test. *P* values are indicated as follows: **p < 0.003, ***p < 0.0002, and ****p < 0.0001. Data are represented as means of replicates ± SD. The underlying data for this figure can be found in S5 Data.

A growing number of GPCRs have been described that localize to and uniquely function at cilia [62]. For many of these cilia-localized GPCRs, it is unclear whether they act via PKA or other effectors. To test whether a ciliary GPCR also affects ciliary PKA activity, we created cilia PKA reporter NIH/3T3 cells stably expressing somatostatin receptor 3 (SSTR3). SSTR3 is a ciliary GPCR which couples to Gα_i/o_ [63–66] (Fig 5A). Stimulating SSTR3-expressing cells with somatostatin (SST) inhibited cilia PKA reporter activity (Fig 5B, 5D). Thus, a ciliary GPCR also regulates ciliary PKA activity.

To assay the dependency Gα_i/o_ on the ability of SSTR3 to inhibit ciliary PKA, we treated cilia PKA reporter cells with pertussis toxin (PTX), an inhibitor of Gα_i/o_ proteins [67,68]. The ability of SST to inhibit cilia PKA reporter activity was blocked by PTX (Fig 5B, 5D), indicating that Gα_i/o_ can control ciliary PKA activity. Interestingly, we previously found that SST activation of SSTR3-expressing fibroblasts induces *Gli1* similarly to SAG, suggesting that activation of ciliary Gα_i/o_ can activate the HH transcriptional response [37]. We conclude that activation of SSTR3 stimulates Gα_i/o_ to inhibit ciliary PKA and, at least in NIH/3T3 cells, drive GLI-mediated HH target gene transcription.

Gα_i/o_ has been investigated as an effector of SMO in HH signal transduction [23–29]. To test whether Gα_i/o_ is required for SMO to inhibit PKA in the primary cilium, we treated cilia PKA reporter-expressing NIH/3T3 cells with SAG and PTX. Unlike SST activation of SSTR3, PTX did not block the ability of SAG activation of SMO to inhibit cilia PKA reporter activity (Fig 5B–5D). To further test whether Gα_i/o_ contributes to SMO-mediated inhibition of ciliary PKA, we repeated the above experiments in the presence of FSK-stimulated PKA activity. In the presence of FSK, SSTR3-mediated inhibition of cilia PKA reporter activity continued to be blocked by PTX (S4A–S4C Fig). Similarly, in the presence of FSK, SMO-mediated inhibition of cilia PKA reporter activity continued not to be blocked by PTX (S4A–S4C Fig). We propose that Gα_i/o_ is critical for ciliary SSTR3-mediated inhibition of ciliary PKA, but dispensable for SMO-mediated inhibition of ciliary PKA.

### SMO A635 contributes to inhibition of ciliary PKA

An alternative to the hypothesis that Gα_i/o_ mediates SMO inhibition of ciliary PKA is that SMO signals by the direct binding and inhibition of PKA-C via a PKI-like motif [29,31,60]. A portion of the SMO proximal carboxy tail (pCT, residues 615–638) resembles PKI motifs of other PKA inhibitory proteins and these residues are necessary for SMO function in zebrafish [31].

**Fig 6:**
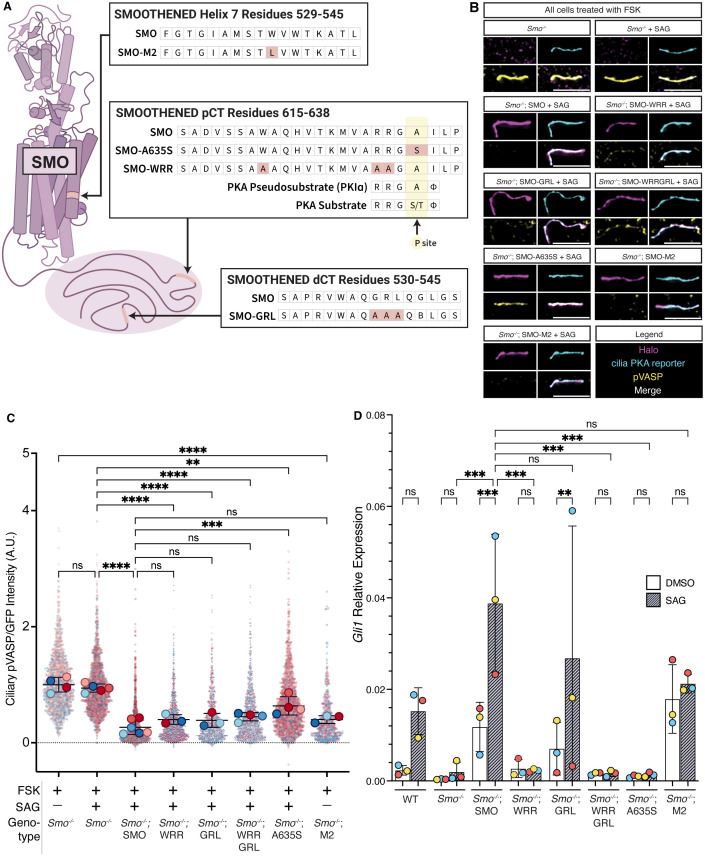
SMO pseudosubstrate site contributes to inhibiting ciliary PKA. **(A)** A schematic of the SMO mutations assessed in this figure. **(B)** *Smo*^−/−^ cilia PKA reporter cells, stably expressing Halo-tagged wild-type SMO, SMO-WRR, SMO-GRL, SMO-WRRGRL, SMO-A635S, or SMO-M2, as indicated. Cells were treated SAG (100 nM for 24 h) and FSK (100 nM for 15 min). Images depict cells stained for pVASP (pVASP^S157^, yellow), cilia PKA reporter (GFP, cyan), and Halo (Halo, magenta). Scale bars are 5 µm. **(C)** Quantification of ciliary pVASP intensity, normalized to ciliary GFP. Significance was determined via one-way ANOVA of the means of each biological replicate, followed by Šídák’s multiple comparison test. **(D)** qRT-PCR of *Gli1* in wild-type or *Smo*^−/−^ cells expressing wild-type SMO, SMO-WRR, SMO-GRL, SMO-WRRGRL, SMO-A635S, or SMO-M2 and treated with SAG, as indicated. Significance was determined via two-way ANOVA of the means of each biological replicate, followed by Šídák’s multiple comparison test. For all plots, each biological replicate is color-coded. *P* values are indicated as follows: **p < 0.003, ***p < 0.0002, and ****p < 0.0001. Data are represented as means of replicates ± SD. The underlying data for this figure are in S6 Data.

Previous work identified that three residues in the pCT (W622, R632, and R633) are critical for SMO function. To test the function of the SMO pCT PKI-like motif, we generated *Smo*^−/−^ cilia PKA reporter NIH/3T3 cells stably expressing Halo-tagged wild-type SMO or a version of SMO in which W622, R632, and R633 are mutated to alanine, referred to as SMO WRR (Fig 6A). As expected, SAG stimulation of wild-type SMO-expressing cells showed inhibited cilia PKA reporter activity (Fig 6B, 6C). Unexpectedly, SAG stimulation of SMO WRR-expressing cells also inhibited cilia PKA reporter activity (Fig 6B, 6C). However, consistent with previous findings [31], SMO WRR failed to induce *Gli1* in response to SAG (Fig 6D). Thus, the SMO WRR mutation in the pCT PKI-like motif inhibits SMO function, but does not block inhibition of ciliary PKA.

Prior work examining the interaction of the SMO C-terminus with PKA-C in HEK293 cells revealed that there may be a second PKI-like motif in the SMO distal carboxy tail (dCT, residues 530–545) [31]. To test the involvement of this additional PKI-like motif, we mutated three residues within the dCT PKI-like motif (G538A, R539A and L540A, referred to as the GRL mutation) (Fig 6A). Similar to wild-type SMO and SMO WRR, SAG stimulation of *Smo*^-/-^ cilia PKA reporter NIH/3T3 cells stably expressing Halo-tagged SMO GRL inhibited cilia PKA reporter activity (Fig 6B-6C). Similar to wild-type SMO, SMO GRL was able to induce *Gli1* in response to SAG (Fig 6D). Thus, mutation of the dCT SMO PKI-like motif neither abrogates the ability of SMO to inhibit ciliary PKA, nor does it block downstream signal transduction.

To test whether the pCT and dCT PKI-like motifs may act redundantly to inhibit PKA, we generated *Smo*^−/−^ cilia PKA reporter NIH/3T3 cells stably expressing Halo-tagged SMO bearing both the WRR and GRL mutations (referred to as SMO WRRGRL). In these cells, activating SMO WRRGRL with SAG also inhibited cilia PKA reporter activity (Fig 6B, 6C), and failed to induce *Gli1* in response to SAG, to a similar extent as SMO WRR (Fig 6D). Therefore, we did not find any evidence of overlapping function of the PKI-like motifs in the pCT and dCT.

Typically, PKA-C phosphorylates its substrates at a serine or threonine at a consensus (RRXS/TΦ where Φ is a hydrophobic residue) phosphorylation site (P site) [69–71]. Pseudosubstrates differ from substrates in having a nonphosphorylatable residue at the P site. Replacement of the P site residue of a PKA pseudosubstrate with serine converts them into substrates and increases dissociation from PKA-C [71,72]. Happ and colleagues previously demonstrated that a version of SMO in which the pCT SMO PKI motif P site alanine was substituted with serine (SMO A635S) did not activate the HH transcriptional response [31].

To assess whether converting the pCT SMO PKI-like motif to a consensus PKA substrate motif affects the ability of SMO to inhibit ciliary PKA, we generated *Smo*^−/−^ cilia PKA reporter NIH/3T3 cells stably expressing Halo-tagged SMO A635S and stimulated them with SAG (Fig 6A). Compared to wild-type SMO, SMO A635S exhibited attenuated inhibition of cilia PKA reporter activity (Fig 6B, 6C). Unlike wild-type SMO, SMO A635S did not induce *Gli1* in response to SAG (Fig 6D). Thus, the SMO pseudosubstrate site is critical for its ability to control ciliary PKA activity and activate the downstream pathway.

An oncogenic single amino acid substitution in SMO, W5535L (better known as SMO-M2), is sufficient to cause basal cell carcinoma, medulloblastoma and rhabdomyosarcoma [73]. To assess whether oncogenic mutations affect the ability of SMO to inhibit ciliary PKA, we generated *Smo*^−/−^ cilia PKA reporter NIH/3T3 cells stably expressing Halo-tagged SMO-M2. Unlike wild-type SMO, SMO-M2 inhibited cilia PKA reporter activity even in the absence of SAG (Fig 6B, 6C). Similarly, SMO-M2 induced *Gli1* even in the absence of SAG (Fig 6D). Thus, an oncogenic mutation constitutively activates the ability of SMO to inhibit ciliary PKA.

To assess whether any of these SMO mutations uncovered a cryptic dependency on Gα_i_, we treated each of the mutant SMO-expressing cells with SAG and PTX. PTX did not attenuate the ability of these mutant forms of SMO to inhibit cilia PKA reporter activity (S5 Fig). We conclude that SMO inhibits ciliary PKA independently of Gα_i_, and that the SMO pseudosubstrate site-mediated inhibition of ciliary PKA activity activates the downstream HH signal transduction pathway.

### GPR161 promotes ciliary PKA activity by localizing PKA-R and PKA-C to the primary cilium

GPR161 inhibits HH signal transduction, can constitutively activate Gα_s_, and exits the cilium upon SMO activation [32–36]. Therefore, we hypothesized that GPR161, present in the cilium in the absence of HH ligand [32], establishes baseline ciliary PKA activity.

**Fig 7:**
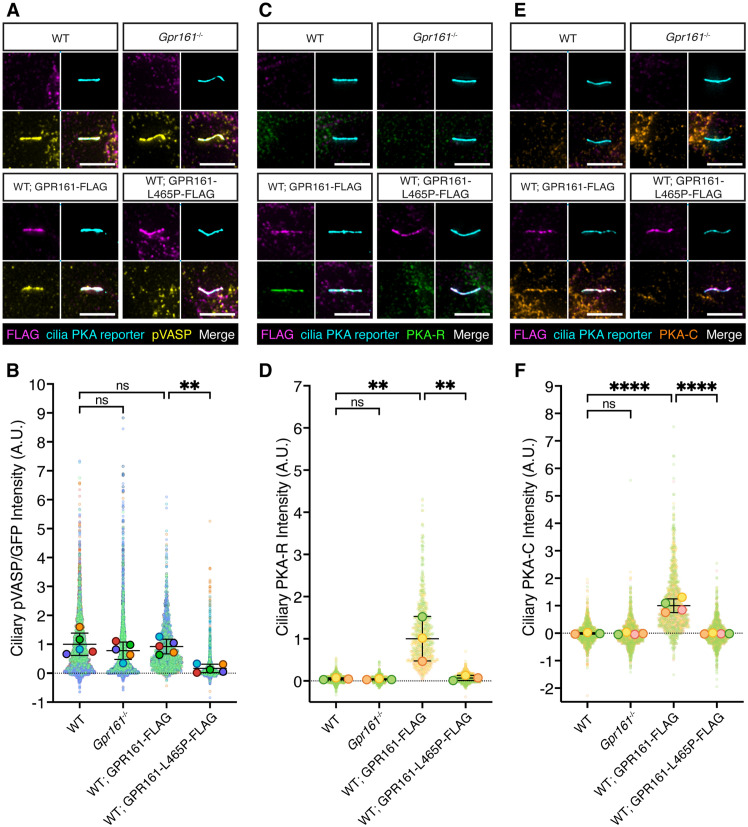
GPR161 AKAP domain promotes ciliary localization of PKA-R and PKA-C. (**A**, **C**, and **E)** Cilia PKA reporter cells, *Gpr161*^−/−^ cilia PKA reporter cells, or wild-type (WT) cilia PKA reporter cells stably expressing FLAG-tagged wild-type GPR161 or GPR161-L465P, which disrupts the AKAP domain of GPR161, as indicated. Images depict cells stained for pVASP (in A, pVASP^S157^, yellow), PKA-R (in C, green), PKA-C (in E, orange), cilia PKA reporter (GFP, cyan), and FLAG (FLAG, magenta). To detect basal levels of pVASP without FSK treatment in this experiment, we used the higher laser power, similar to the experiment described in Fig 5B. Scale bars are 5 µm. **(B)** Quantification of ciliary pVASP intensity, normalized to ciliary GFP. **(D)** Quantification of ciliary PKA-R intensity. **(F)** Quantification of ciliary PKA-C intensity. For all plots, each biological replicate is color-coded. Significance was determined via one-way ANOVA of the means of each biological replicate, followed by Šídák’s multiple comparison test. *P* values are indicated as follows: **p < 0.003, ***p < 0.0002, and ****p < 0.0001. Data are represented as means of replicates ± SD. The underlying data for this figure are in S7 Data.

To test this hypothesis, we used CRISPR to delete *Gpr161* in the ciliary PKA reporter NIH/3T3 cell line. Inconsistent with the hypothesis, deletion of *Gpr161* did not alter baseline levels of ciliary PKA reporter activity (Fig 7A, 7B). We further hypothesized that, because GPR161 is constitutively active [34,36,74], increased GPR161 would increase ciliary PKA reporter activity. Again, inconsistent with the hypothesis, overexpression of FLAG-tagged GPR161 did not increase cilia PKA reporter activity (Fig 7A, 7B). We conclude that GPR161 is not critical for setting baseline ciliary PKA activity, at least in NIH/3T3 cells.

Among GPCRs, GPR161 is unusual in that it possesses an AKAP domain in its C-terminus through which it directly binds PKA-R [75]. A point mutation in the AKAP amphipathic helix, L465P, disrupts the ability of GPR161 to bind PKA-R [36,75]. To test whether the AKAP domain of GPR161 contributes to ciliary PKA activity, we expressed FLAG-tagged GPR161 L465P. GPR161 L465P overexpression decreased ciliary PKA reporter activity (Fig 7A, 7B). One possible interpretation of these results is that GPR161 can both promote and inhibit ciliary PKA activity, with the AKAP domain being critical for the promotion of ciliary PKA activity.

The AKAP domain binds PKA-R, a negative regulator of PKA activity [76]. To test this hypothesis, we immunostained control and GPR161-overexpressing cells for PKA-R. Increased GPR161 increased the amount of PKA-R in cilia (Fig 7C, 7D). In contrast, increased GPR161 L465P did not (Fig 7C, 7D). Thus, GPR161 promotes ciliary PKA-R localization in a way that depends on the AKAP domain.

AKAP domain-dependent recruitment of PKA-R, a negative regulator of PKA activity, to cilia raised the question of how the AKAP domain promotes ciliary PKA activity. We hypothesized that one possible explanation for this apparent incongruity could be that the AKAP domain also recruits, perhaps indirectly, PKA-C to the cilium. To test this hypothesis, we immunostained control and GPR161-overexpressing cells for PKA-C. As with PKA-R, increased GPR161 increased the amount of PKA-C in cilia (Fig 7E, 7F). In contrast, increased GPR161 L465P did not (Fig 7E, 7F). Thus, GPR161 via its AKAP domain promotes the localization of both PKA-R and PKA-C to cilia.

## Discussion

Although vertebrate SMO requires the cilium to activate the downstream HH signal transduction pathway, how it does so has been elusive. Recent revelatory work has discovered that the C-terminus of vertebrate SMO can bind and inhibit PKA [29,31]. PKA phosphorylates GLI to inhibit HH target gene transcription [15–17]. Because SMO, PKA, and GLI all can localize to primary cilia [13,15,37,60], we investigated whether SMO signals through inhibiting PKA at the primary cilium. To test this hypothesis, we developed a reporter that measures ciliary PKA activity.

This reporter revealed that SMO inhibits ciliary PKA in a state-dependent manner. SHH (which acts through its receptor PTCH1 to activate SMO) [77] and SAG (which directly binds and activates SMO) [50,51] both induced SMO to accumulate at cilia and inhibit ciliary PKA. In contrast, CYA, a SMO inhibitor [52,78], induced SMO to accumulate at cilia but did not inhibit ciliary PKA. Thus, the accumulation of SMO in cilia is not sufficient to inhibit ciliary PKA; SMO must also be in an active state.

Another requirement for SMO to inhibit ciliary PKA is GRK2/3: SMO is phosphorylated by GRK2/3 [60] and pharmacological inhibition of GRK2/3 prevented SMO from inhibiting ciliary PKA. Consistent with this conclusion, CYA prevented GRK2/3 from phosphorylating SMO and from inhibiting ciliary PKA. GRK2/3 also phosphorylates GPR161, facilitating the removal of GPR161 from the primary cilium [33], but GRK2/3 can still regulate HH signaling in the absence of GPR161 [34]. Thus, another way in which GRK2/3 participates in HH signal transduction is by phosphorylating SMO to suppress ciliary PKA.

By measuring ciliary PKA reporter activity, we assessed molecular mechanisms by which SMO could control PKA. Two models have been: 1) SMO activates Gα_i_ to inhibit production of cAMP, thereby inhibiting PKA [23–27], and 2) SMO acts like a PKI to directly bind and inactivate PKA [29,31]. We found that Gα_i_ activity is dispensable for SMO to inhibit ciliary PKA in NIH/3T3 cells. Yet, activating Gα_i_ via a cilia-localized Gα_i_-coupled GPCR also inhibited ciliary PKA and activated the HH transcriptional response. Thus, while SMO inhibits ciliary PKA independent of Gα_i_, cAMP-mediated activation of ciliary PKA is sufficient to repress the HH signal transduction pathway.

NIH/3T3 cells are unlikely to faithfully model HH signal transduction in all cell types. Therefore, it is possible that Gα_i_-coupling is a mechanism by which SMO inhibits ciliary PKA activity in other cell types.

A mutation predicted to turn the PKI-like pseudosubstrate within the C-terminus of SMO into a PKA substrate blocked the ability to SMO to inhibit PKA and activate downstream transcription. As PKA substrates dissociate from PKA upon phosphorylation [71,72], we propose that converting SMO from a pseudosubstrate to a substrate increases its dissociation rate from PKA-C, disrupts its ability to inhibit PKA and abrogates its ability to activate the downstream signal transduction pathway.

Unexpectedly, mutating other residues in the PKI-like motif in the SMO proximal carboxy tail inhibited activation of the downstream signal transduction pathway, but did not abrogate inhibition of the ciliary PKA reporter. Disrupting PKI-like motifs in both the proximal and distal carboxy tail of SMO also did not prevent inhibition of the ciliary PKA reporter, arguing against redundancy between PKI-like motifs in SMO. One possibility is that these PKI-like motifs do inhibit ciliary PKA, but that the reporter is not sensitive enough to detect this inhibition. Another possibility is that PKI activity is robust to the mutations tested. A third possibility is that SMO activates the downstream signal transduction pathway via two required mechanisms, one of which depends on pseudosubstrate-mediated inhibition of PKA and another of which is disrupted by the SMO WRR mutations.

These results indicate that HH-mediated activation of SMO turns off ciliary PKA. In the absence of HH signals, SMO is not in the cilium and ciliary PKA is active. In this context, what activates ciliary PKA? One possibility was that GPR161 signals constitutively through Gα_s_ to set basal PKA activity. Inconsistent with this possibility, GPR161 was dispensable for basal ciliary PKA activity.

These results raise the possibility that other proteins promote ciliary PKA activity to keep the HH pathway off in the absence of HH signals. Indeed, Pusapati and colleagues previously found that GPR161 is dispensable for keeping the HH pathway off in the absence of HH signals in NIH/3T3 cells [34]. Additionally, the ventralization of the mouse neural tube in the absence of GPR161 is less than that caused by loss of PTCH1 or PKA [32,79,80], suggesting that GPR161 may not be the only activator of PKA activity relevant to HH signal transduction.

Increased GPR161 increased the ciliary localization of both PKA-R and PKA-C, in a way that depended on the AKAP domain within the carboxy terminus of GPR161. Previously, Bachmann and colleagues demonstrated that GPR161 recruits PKA-RIα to primary cilia, Tschaikner and colleagues demonstrated that GPR161 can biochemically interact with PKA-R and PKA-C, and May and colleagues demonstrated that HH signaling can stimulate the departure of PKA-RIα from primary cilia [35,38,75]. Our results are consistent with these previous findings, and further support that GPR161 recruits both PKA-R and PKA-C to the cilium. Recently, a different ciliary GPCR, GPR45 was found to bring Gα_s_ to the cilium to support the ability of another ciliary GPCR, melanocortin 4 receptor (MC4R), to stimulate adenylyl cyclase [81]. Thus, multiple GPCRs may function to transport other proteins to the cilium.

**Fig 8:**
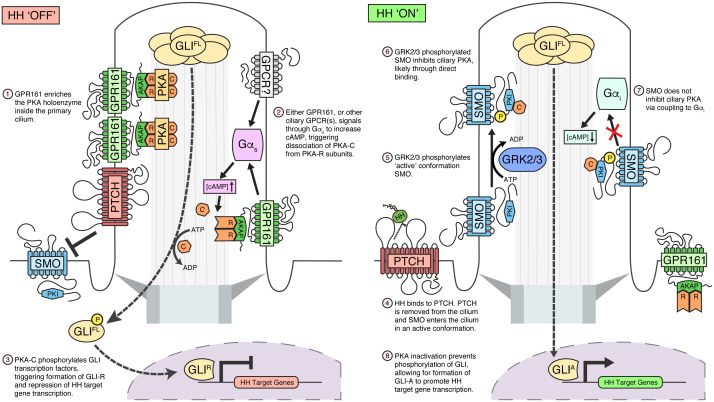
Model for HH-mediated control of ciliary PKA activity. GPR161 recruits the PKA holoenzyme to the primary cilium. There, locally elevated levels of cAMP promote dissociation of PKA-R from PKA-C subunits. PKA-C phosphorylates GLI, promoting processing into GLI-R and repressing HH target gene transcription in the nucleus. When HH binds to PTCH, PTCH leaves the cilium and SMO enters in an active conformation. GRK2/3 phosphorylates active SMO. Phosphorylated SMO directly binds and inhibits the catalytic site of PKA-C. PKA does not phosphorylate GLI, and GLI-A induces the HH transcriptional program in the nucleus.

These data suggest a model of HH signal transduction (Fig 8). In the absence of HH stimuli, GPR161 enriches the PKA holoenzyme inside the primary cilium. Then, GPR161, possibly acting with other ciliary GPCRs, activates Gα_s_ and adenylyl cyclase to increase cAMP concentrations, facilitating dissociation of PKA-R from PKA-C. Activated PKA-C phosphorylates GLI transcription factors, triggering formation of GLI-R, which represses HH target gene transcription in the nucleus.

In the presence of HH stimuli, HH binds to PTCH, allowing SMO to accumulate in the cilium in an active conformation. Ciliary SMO is phosphorylated by GRK2/3 and then binds to and inhibits ciliary PKA. Turning off PKA allows GLI transcription factors to become activated (GLI-A), which induce HH target gene transcription.

One limitation of the ciliary PKA reporter is that it does not assess PKA activity outside of the cilium. Thus, it remains unclear whether SMO or ciliary GPCRs also affect PKA activity outside of the cilium.

An oncogenic form of SMO constitutively inhibits ciliary PKA activity. Oncogenic mutations in SMO cause a number of cancers, including medulloblastoma, basal cell carcinoma and rhabdomyosarcoma [82]. Our findings suggest that, for SMO, constitutive ciliary localization, constitutive ciliary PKA inhibition and oncogenic activity are causally related. Although HH pathway-related medulloblastoma is responsive to small molecule inhibitors of SMO, acquisition of SMO mutations that interfere with drug binding lead to recurrence [83,84]. We propose that an alternative therapeutic approach that bypasses SMO is to reactivate ciliary PKA.

Interestingly, SSTR3, a ciliary Gα_i_-coupled GPCR not implicated in regulating HH signaling, could also inhibit ciliary PKA and activate HH transcription when expressed in NIH/3T3 cells. As the primary cilium is a specialized microenvironment for multiple receptors and signaling pathways, how many are mediated via ciliary PKA? A burgeoning number of other GPCRs have more recently been found to localize to primary cilia across many different tissues [32,85–89]. For example, MC4R is a neuronal cilium-localized GPCR critical for the control of feeding behavior and long-term energy homeostasis [89–92]. Similarly, SSTR3 endogenously localizes to the primary cilia of mammalian neurons and is implicated in learning and memory [63,64]. One possibility is that there are different effectors other than PKA for other ciliary GPCRs. Another possibility is that many ciliary GPCRs act through ciliary PKA but achieve cell type-specific effects via different effectors downstream of PKA.

Moreover, it is unclear if cells simultaneously communicate via multiple ciliary signaling pathways, such as the HH signal transduction pathway and ciliary GPCR signaling pathways. It is possible that the set of cells competent for HH signaling and the set of cells that are competent for ciliary GPCR signaling are mutually exclusive, thus preventing crosstalk within their cilia. Alternatively, all Gα_i_- or Gα_s_-coupled ciliary GPCRs in cells competent for HH signaling may influence GLI-dependent transcription. Untangling GPCR signaling at the primary cilium will be critical to understanding not only HH signaling, but also how a host of signals critical to human health are transduced.

## Materials and methods

### Ethics statement

All zebrafish protocols were approved by the Institutional Animal Care and Use Committee (IACUC) of the University of California, San Francisco, Protocol #: AN194955-01F.

### Vector construction and generation of stable cell lines

To generate NIH/3T3 Flp-In cell lines expressing the ciliary PKA reporter, ARL13B-GFP-VASP was cloned with the In-Fusion HD cloning kit (Takara, 639650) into a version of pgLAP5 with an attenuated EF1a promoter lacking the TATA box [93], a backbone previously generated in our lab [37]. We transfected cells with this plasmid, concurrently with the pOG44 Flp-Recombinase Expression Vector (Invitrogen, V600520), with Lipofectamine LTX (Invitrogen, 15338100), according to the ThermoFisher Flp-In System protocol to generate stable Flp-In expression cell lines. Cells were selected with 70 µg/mL of hygromycin B (Corning, 30–240-CR). Following selection, we selected a single clone of these cells to characterize and build all subsequent cell lines.

For generating mRNA encoding ARL13B-GFP-VASP, ARL13B-GFP-VASP was cloned into the pCS107 expression vector using the In-Fusion HD cloning kit (Takara, 639650).

To generate cell lines expressing Halo-tagged SSTR3, SMO, SMO-WRR, SMO-GRL, SMO-WRRGRL, SMO-A635S and SMO-M2, we cloned the coding region of each protein of interest into a pLVX-TetOne-Puro backbone (Takara 631849). To generate cell lines expressing FLAG-tagged GPR161 and GPR161-L465P, we cloned the coding sequence of each into a pLVX-EF1a-dTATA-Neo backbone.

We used CRISPR-mediated editing to generate loss-of-function mutations in *Smo* and *Gpr161* using two different guide RNAs per gene in our cilia PKA reporter cells. Synthetic guide RNAs for mouse *Smo* (5′-CCCACGCACGGGGCGGCCAG-3′, 5′-UCCCGCUCAAGGCCGCCCCC-3′), and *Gpr161* (5′ AUGCGGUGAGCAGAGCAUGC-3′, 5′- GAGGGAGGAGUUGAGGCUCA-3′) were ordered from Synthego, complexed with TrueCut Cas9 Protein v2 (Invitrogen, A36496), and nucleofected with the Neon Transfection System (Invitrogen). Cells were clonally selected and screened via PCR for genomic deletions with primers for the genomic regions of interest for mouse *Smo* (forward primer: 5′-AGGGTTCCCAGGGTTGAAGA-3′, reverse primer: 5′-CACACGTTGTAGCGCAAAGG-3′) and mouse *Gpr161* (forward primer: 5′- GGAGGTTCCAAACACATTGGC-3′, reverse primer: 5′-CGATGAACTCAGAGACGGCA-3′).

Lentivirus was generated by transfecting 7.5 μg of each plasmid of interest with 1.5μg of pCMV-VSV-G (Addgene, 8454) and 6 μg of psPAX2 (Addgene, 12260) into a 10 cm plate of Lenti-X 293T cells (Takara, 632180) at 70%–80% confluence using Fugene 6 transfection reagent (Promega, E2691). Medium containing lentiviral particles was collected 1 day after transfection and was concentrated with a Lenti-X Concentrator (Takara, 631232), incubated overnight at 4 °C, and followed by centrifugation at 4000 *g* for 45 min at 4 °C. The pellet was resuspended in 120 μL DPBS (Gibco, 14-90-250).

To generate SMO expression cell lines, cilia PKA reporter *Smo*^−/−^ cells were transduced with 20 μL of resuspended lentivirus containing either doxycycline-inducible SMO-Halo, SMO-A635S-Halo, and SMO-M2-Halo as well as puromycin resistance in the presence of 4 μg/mL polybrene. Twenty-four hours after transduction, cells were selected with 1 μg/mL puromycin (Gibco, A11138-03) for 5 days. Cells that survived selection were then incubated with 100ng/mL doxycycline (Sigma-Aldrich, D5207) for 48 h and incubated in HaloTag Alexa Fluor 660 Ligand (Promega, G8472) overnight before being enriched via fluorescence-activated cell sorting (FACS) on a BD FACSAria III Cell Sorter.

To generate SSTR3 expression cell lines, cilia PKA reporter cells were transduced with lentivirus containing doxycycline-inducible SSTR3-Halo and puromycin resistance in the presence of 4 μg/mL polybrene. Cells were then transduced with 1 μg/mL doxycycline (Sigma-Aldrich, D5207) for 48 h, selected with 1 μg/mL puromycin (Gibco, A11138-03). Cells were then incubated in HaloTag Alexa Fluor 660 Ligand (Promega, G8472) overnight before being enriched via fluorescence-activated cell sorting (FACS) on an BD FACSAria III Cell Sorter.

To generate GPR161 expression cell lines, cilia PKA reporter cells were transduced with lentivirus encoding GPR161-FLAG and neomycin resistance in the presence of 4 μg/mL polybrene. Cells were then selected with 1 mg/mL geneticin selective antibiotic (G418 sulfate) (Gibco, 10131035).

### mRNA synthesis

To generate mRNA for expressing the cilia PKA reporter in zebrafish, we grew pCS107-ARL13B-GFP-VASP in *dam*^*–*^*/dcm*^*–*^ competent *E. coli* (New England Biolabs, C2925H). We isolated our plasmid with the Plasmid Plus Midi Kit (QIAGEN, 12943). For zebrafish injections, we linearized the construct with *ApaI* (New England Biolabs, R0114L) and generated mRNA with the mMESSAGE mMACHINE SP6 kit (Invitrogen, AM1340).

### Zebrafish husbandry and mRNA injection

Adult *Danio rerio* zebrafish were maintained under standard laboratory conditions. Zebrafish of Ekkwill (EKW) background were used as wild-type. Embryos were maintained in egg water containing 60 μg/mL sea salt (Instant Ocean) in distilled water. We injected 500 pg of ARL13B-GFP-VASP mRNA at the one-cell stage. We incubated injected embryos in egg water, and unfertilized embryos were removed 6 h post injection. All zebrafish protocols were approved by the IACUC of the University of California, San Francisco.

### Mammalian cell culture

NIH/3T3 Flp-In cells (Invitrogen, R761-07) were cultured in Dulbecco’s modified Eagle’s medium with high glucose (Gibco, 11965118) supplemented with 10% newborn calf serum (Gibco, 16010159) and GlutaMAX supplement (Gibco, 35050061). Cells were treated with antibiotic-antimycotic (Gibco, 152400632) following FACS for 1 week. Cells were otherwise maintained in the absence of antibiotics. To induce ciliation, cells were grown to confluence and starved overnight in Opti-MEM reduced serum medium with GlutaMAX supplement (Gibco, 51985091).

### Immunofluorescence staining

We seeded cells on 12 mm cover glasses of 170 μm thickness (Paul Marienfeld, 0117520) at a density of 8 × 10^4^ cells per well in a 24-well plate. After drug treatment and induction of ciliation, we fixed cells for 10 min in 4% PFA (VWR, 100504-782) diluted in DPBS (Gibco, 14-90-250). We diluted primary antibodies in blocking buffer (0.1% TritonX-100, 0.02% Sodium Azide, 3% BSA) and incubated them overnight at 4 °C. Subsequently, we incubated cells in donkey Alexa Fluor-conjugated secondary antibodies, ChromoTek GFP-Booster Alexa Fluor 488 (Proteintech, gb2AF488), and Hoechst 33342 (ThermoFisher Scientific, H1399) diluted in blocking buffer at room temperature for 1 h. We mounted cover glasses in ProLong Glass antifade mountant (Invitrogen, P36982) and allowed the slides to cure overnight at room temperature before imaging.

We used the following primary antibodies: Anti-VASP (phospho S157) antibody [5C6] (Abcam, ab58555), Anti-SMO (phospho pS594/pT597/pS599) antibody (7TM, 7TM0239A-IC), Anti-HaloTag antibody (Promega, G9281), Anti-Acetyl-ɑ-Tubulin Lys40 (Cell Signaling Technology, 5335), Anti-FLAG [DYKDDDDK] (Cell Signaling Technology, 2368), Anti-GPR161 (gift from Saikat Mukhopadyhay), Anti-PKA-C (BD Biosciences 610981), and Anti-PKA-R (BD Biosciences 610165).

We fixed dechorionated zebrafish embryos in 4% PFA (VWR, 100504-782) diluted in DPBS (Gibco, 14-90-250) for 2 h at room temperature. We blocked embryos in 1% BSA, 1% DMSO, and 0.5% Triton X-100 in PBS (PBDT) for 1 h. After blocking, we incubated embryos overnight at 4 °C with primary antibodies diluted in PBDT. Subsequently, we incubated embryos in donkey Alexa Fluor-conjugated secondary antibodies, ChromoTek GFP-Booster Alexa Fluor 488 (Proteintech, gb2AF488), and Hoechst (ThermoFisher Scientific) diluted in PBDT for 2 h at room temperature. We incubated embryos in 70% glycerol overnight, then mounted in ProLong Glass antifade mountant (Invitrogen, P36982) and allowed the slides to cure overnight at room temperature before imaging.

### Image acquisition and ciliary fluorescence intensity quantification

We imaged fixed cells on a DeltaVision-OMX-SR (GE Healthcare) equipped with a Plan ApoN 60X/1.42 Oil objective and three PCO.edge 5.5 15bit sCMOS Cameras (liquid cooled). Four-channel fluorescence imaging was captured with a Toptica 4 line laser launch light source, laser excitation wavelengths 405 nm/488 nm/568 nm/642 nm, and emission filters 435/31 m, 528/48 m, 609/37 m, and 683/40 m. To detect basal levels of pVASP without FSK treatment in Figs 7 and 5D, we used a higher laser power (20% in 568 nm excitation) than in the other figures (10% in 568 nm excitation).

Images for quantification were acquired using the widefield setting, and representative images, where indicated in the figure legend, were acquired with 3D-SIM Data. Immersion oil with refractive index of 1.518 was used for most experiments. Z stacks of 5–6 µm were collected using a 0.250 µm step size for widefield imaging and 0.125 µm step size for 3D-SIM imaging. Raw images were reconstructed using SoftWorx 6.5.2 (GE Healthcare) using default parameters.

We imaged fixed zebrafish embryos with Zeiss LSM 800 laser scanning confocal microscope equipped with a 63x/1.4 oil immersion objective and captured using the Zen Imaging Software (Zeiss). While collecting images of zebrafish, we held constant the gain, offset and laser power for each antibody combination. We processed images identically and used ImageJ/FIJI software [94] to generate sum and maximal projections.

We used Cell Profiler image analysis software [95] on our sum projection images to generate fluorescence intensity quantifications. A cilia marker (ARL13B-GFP-VASP) was used to identify cilia and create a mask using the object identification module in CellProfiler using differences in signal intensity and size to segment cilia. The ciliary mask was then dilated by 10 pixels to create a dilated ciliary mask. We determined the fluorescence intensity (integrated intensity) and area (in pixels) for both the cilia mask and dilated cilia mask. We determined background-subtracted, area-normalized ciliary fluorescence intensity for all channels of interest with the following formula:


$$\begin{array}{c} Background\;Ciliary\;Intensity\;(A.U.)\;=\;\frac{{\left(Dilated\;Cilia\right)}_{Intensity}-\;{\left(Cilia\right)}_{Intensity}}{(Dilated\;Cilia{)}_{Area}-(Cilia{)}_{Area}} \end{array}$$

$$\begin{array}{c} Ciliary\;Intensity\;(A.U.)=\;\frac{(Cilia{)}_{Intensity}}{(Cilia{)}_{Area}}-Background\;Ciliary\;Intensity\;(A.U.) \end{array}$$

To represent ciliary PKA activity, we report $\frac{ciliary\;pVASP\;Intensity}{ciliary\;GFP\;Intensity}$ to account for the amount of VASP peptide able to be phosphorylated at the primary cilium in our cilia PKA reporter cells. Ciliary intensity calculations were ultimately done through a Jupyter Notebook python script. Data were exported to  .csv files and graphs were generated in GraphPad Prism 10. Statistical analyses were performed using GraphPad Prism 10. Statistical tests used for each experiment are listed in the accompanying figure legend.

### Immunoblotting

Cells were lysed using RIPA buffer (150 nm NaCl, 50 mM Tris, pH 7.6, 0.1% SDS, 0.1% NP-40, and 0.5% sodium deoxycholate) supplemented with protease inhibitors (Roche). Protein concentration was determined using a Pierce BCA Protein Assay Kit (Thermo Fisher Scientific). Protein samples were separated on 4%–15% gradient TGX precast gels (Bio-Rad) and transferred to PVDF membrane (Bio-Rad). 5% nonfat dried milk in TBS with 0.1% Tween was used to block membranes and to dilute antibodies. HRP signal was detected using Clarity Western ECL substrate (Bio-Rad). Primary antibodies used were rabbit anti-GPR161 (gift from Saikat Mukhopahyay) and mouse anti-β-actin (Proteintech, AB_2687938). We used HRP-conjugated secondary antibodies. (Jackson ImmunoResearch Laboratories, Inc).

### Quantitative RT-PCR

NIH/3T3 cells were seeded in 6-well plates at a density of 300,000 cells per well. Total RNA was extracted using the RNeasy Mini Kit (QIAGEN Cat# 74106) according to the manufacturer’s instructions. cDNA was reverse transcribed from 1 μg of RNA using the iSCRIPT cDNA synthesis kit (Bio-Rad Cat#1708891BUN). Each qRT-PCR reaction was performed in technical quadruplicates on a 384 well plate (USA Scientific, Cat# 1438-4700) using PowerUp SYBR Green Master Mix (Applied Biosystems Cat# A25777) and run on a QuantStudio 5 real-time PCR system (Applied Biosciences) running QuantStudio Design and Analysis software (v.1.5.1). We used the following primer sequences: *Hprt* (Forward primer: 5′-CATAACCTGGTTCATCATCGC-3′, Reverse primer: 5′-TCCTCCTCAGACCGCTTT T-3′) and *Gli1* (Forward primer: 5′-GGTGCTGCCTATAGCCAGTGTCCTC-3′, Reverse primer: 5′-GTGCCAATCCGGTGG AGTCAGACCC-3′) Relative expression was calculated using the ΔΔCT method normalized to the expression of the housekeeping gene *hprt*.

## Supporting information

S1 Fig Characterizing the dynamic range of the cilia PKA reporter. **(A–C)** Representative images of immunofluorescence staining of NIH/3T3 cells stably expressing the cilia PKA reporter. Cells were serum-starved and then treated with either vehicle, FSK (100 nM for 15 min), or both FSK and H89 (100 nM and 20 μM, respectively, for 15 min). Images depict cells stained for pVASP (pVASP^S157^, yellow), cilia PKA reporter (GFP, cyan), and nuclei (Hoechst, gray). Scale bar, 10 μm. **(D)** Quantification of ciliary pVASP intensity normalized to ciliary GFP intensity of cells treated with different concentrations of FSK. **(E)** Quantification of ciliary pVASP intensity normalized to ciliary GFP intensity cells treated with FSK for different durations. Significance was determined via one-way ANOVA followed by Tukey’s multiple comparison test. (****p < 0.0001. Data are represented as means ± SD.) The underlying data for this figure are in S1 Data.
(TIF)

S2 Fig SAG and cyclopamine induce equivalent levels of SMO accumulation at primary cilia. **(A)** Clustal Omega alignment of Sanger sequencing of the genomic sequence from *Smo^+/+^*  ^*(*^WT) cilia PKA reporter clone, as well as *Smo*^*−/−*^ cilia PKA reporter clone. NIH/3T3 cells are hypertriploid. The *Smo*^*−/−*^ cilia PKA reporter clone has 3 different alleles. Allele 1 has a 10 bp and 1 bp deletion allele resulting in an early frameshift and early stop codon. Allele 2 has an 85 bp deletion resulting in an early frameshift and an early stop codon. Allele 3 has a 139 bp deletion that deletes the start codon. Stop codons are indicated with red arrows. Visualization adapted from Benchling. **(B–E)** Immunofluorescence images of cilia PKA reporter cells treated with the same regimes as in Fig 3B–3D, 3G. Images depict cells stained for SMO (SMO, yellow), basal bodies (CEP43, cyan), cilia PKA reporter (GFP, magenta), and nuclei (Hoechst, gray). Scale bars for larger images, 5 μm. Scale bars for insets are 2.5 μm. **(F)** Quantification of ciliary SMO localization from A–D. For all plots, each biological replicate is color-coded. Significance was determined via one-way ANOVA of the means of each biological replicate, followed by Šídák’s multiple comparison test. ****p < 0.0001. Data are represented as means of replicates ± SD. The underlying data for this figure are in S3 Data.
(TIF)

S3 Fig Ciliary pVASP and ciliary pSMO intensities are anti-correlated. Each dot represents a cilium of a cilia PKA reporter cell treated with 15 min of 75 nM FSK, or SAG (1 nM, 2 nM, 5 nM, 10 nM or 50 nM) for 24 h followed by 15 min of 75 nM FSK. These data are also used in Fig 3F, 3G. The x-axis represents the level of ciliary pSMO in each cilium, and the y-axis represents the level of ciliary pVASP/GFP in that same cilium. The underlying data for this figure is in S4 Data.
(TIF)

S4 Fig Gα_i_ inhibits ciliary PKA. **(A)** Immunofluorescence imaging of cilia PKA reporter cells stably expressing Halo-tagged SSTR3 in response to doxycycline. Cells were serum-starved and treated with either FSK (100 nM for 15 min), SST and FSK (10 µM for 2 h and 100 nM for 15 min, respectively), or SST, PTX, and FSK (10 µM for 2 h, 100 ng/mL for 16 h, and 100 nM for 15 min, respectively). Images depict cells stained for pVASP (pVASP^S157^, yellow), cilia PKA reporter (GFP, cyan), and Halo (Halo, magenta). Scale bars are 5 µm. **(B)** Immunofluorescence imaging of cilia PKA reporter cells, performed as in A. Cells were serum-starved and treated with either FSK (100 nM for 15 min), SAG and FSK (100 nM for 24 h and 100 nM for 15 min, respectively), or SAG, PTX, and FSK (100 nM for 24 h, 100 ng/mL for 16 h, or 100 nM for 15 min, respectively). Scale bars are 5 µm. **(C)** Quantification of ciliary pVASP intensity, normalized to ciliary GFP, of A and B. Distinct biological replicate are represented with distinct colors. Significance was determined via one-way ANOVA of the means of each biological replicate, followed by Šídák’s multiple comparison test. *P* values are indicated as follows: **p < 0.003, ***p < 0.0002, and ****p < 0.0001. Data are represented as means of replicates ± SD. The underlying data for this figure is in S5 Data.
(TIF)

S5 Fig Gα_i/o_ does not synergize with the SMO PKI-like motif to inhibit ciliary PKA. Quantification of ciliary pVASP intensity, normalized to ciliary GFP, in *Smo*^−/−^ cilia PKA reporter cells expressing wild-type SMO, SMO-WRR, SMO-GRL, SMO-WRRGRL, SMO-A635S, or SMO-M2, as indicated. As indicated, cells were treated SAG (100 nM for 24 h), or SAG and PTX (100 nM for 24 h and 100 ng/mL for 16 h, respectively). All conditions were treated with FSK (100 nM for 15 min). For all plots, each biological replicate is color-coded. Same data as used in Fig 6. Significance was determined via one-way ANOVA of the means of each biological replicate, followed by Šídák’s multiple comparison test. *P* values are indicated as follows: **p < 0.003, ***p < 0.0002, and ****p < 0.0001. Data are represented as means of replicates ± SD. The underlying data for this figure is in S6 Data.
(TIF)

S6 Fig GPR161 does not affect basal levels of ciliary PKA activity in NIH/3T3 cells. **(A)** Clustal Omega alignment of Sanger sequencing of the genomic sequence from *Gpr161^+/+^* (WT) cilia PKA reporter clone, as well as *Gpr161*^*−/−*^ cilia PKA reporter clone. The *Gpr161*^*−/−*^ cilia PKA reporter clone has a 38 bp deletion resulting in an early frameshift and an early stop codon, indicated by the red arrow. Visualization adapted from Benchling. **(B)** Immunoblot of lysates from cilia PKA reporter cells and *Gpr161*^−/−^ cilia PKA reporter cells. GPR161 is 60kDa. Blotting for β-actin controls for loading.
(TIF)

S1 Raw Images Original images for blot in S6B Fig. **(Page 1)** Immunoblot of lysates from cilia PKA reporter cells and *Gpr161*^−/−^ cilia PKA reporter cells with rabbit anti-GPR161 from Saikat Mukhopadhyay. **(Page 2)** Merge of the brightfield ladder and rabbit anti-GPR161 immunoblot. **(Page 3)** Immunoblot of lysates from cilia PKA reporter cells and *Gpr161*^−/−^ cilia PKA reporter cells with anti-β-actin as a loading control.
(PDF)

S1 Data Data underlying Figs 1 and S1. (XLSX)

S2 Data Data underlying Fig 2. (XLSX)

S3 Data Data underlying Figs 3 and S2. (XLSX)

S4 Data Data underlying Figs 4 and S3. (XLSX)

S5 Data Data underlying Figs 5 and S4. (XLSX)

S6 Data Data underlying Figs 6 and S5. (XLSX)

S7 Data Data underlying Fig 7. (XLSX)

## Abbreviations

AKAP: A-kinase anchoring protein
cAMP: cyclic adenosine monophosphate
CRISPR: clustered regularly interspaced short palindromic repeats
CYA: cyclopamine
FACS: fluorescence-activated cell sorting
FSK: forskolin
GPCR: G protein-coupled receptor
GRK2/3: G protein-coupled receptor kinases 2 and 3
HH: Hedgehog
IACUC: Institutional Animal Care and Use Committee
MC4R: melanocortin 4 receptor
PKA: protein kinase A
PKIs: protein kinase inhibitors
pSMO: phosphorylated SMO
pVASP: phosphorylated vasodilator-stimulated phosphoprotein
PTX: pertussis toxin
SAG: SMO agonist
SHH: Sonic hedgehog
SMO: Smoothened
SST: somatostatin
SSTR3: somatostatin receptor 3
VASP: vasodilator-stimulated phosphoprotein
WT: wild-type.
