# Supplementary figures and images for: Smoothened and ciliary GPCRs regulate ciliary protein kinase A activity involved in Hedgehog signal transduction
Source: PLoS Biol. 2026 Jun 10;24(6):e3003841. doi: 10.1371/journal.pbio.3003841 (PMC13289941; doi:10.1371/journal.pbio.3003841)

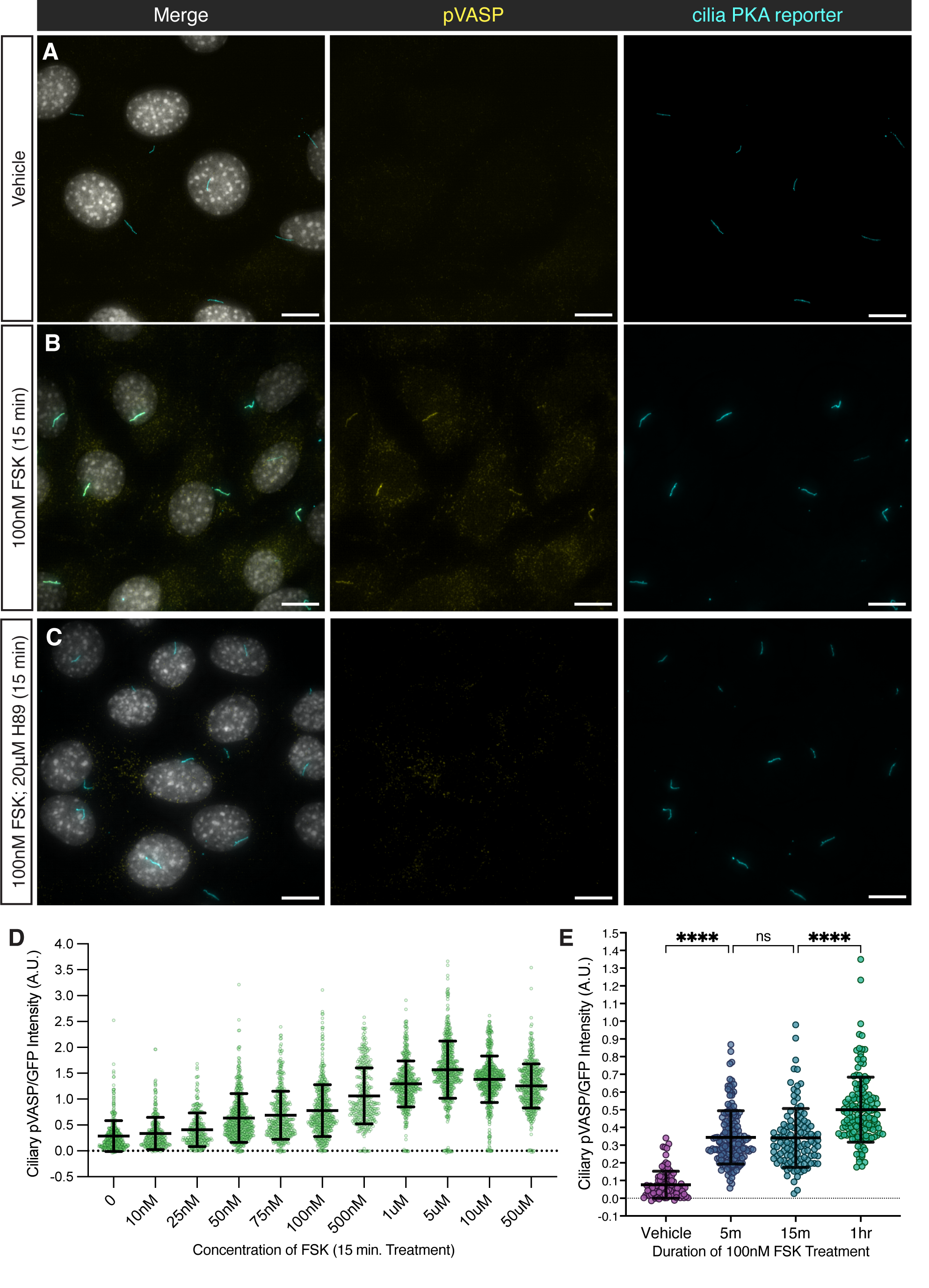

Supplement: S1 Fig — (A–C) Representative images of immunofluorescence staining of NIH/3T3 cells stably expressing the cilia PKA reporter. Cells were serum-starved and then treated with either vehicle, FSK (100 nM for 15 min), or both FSK and H89 (100 nM and 20 μM, respectively, for 15 min). Images depict cells stained for pVASP (pVASPS157, yellow), cilia PKA reporter (GFP, cyan), and nuclei (Hoechst, gray). Scale bar, 10 μm. (D) Quantification of ciliary pVASP intensity normalized to ciliary GFP intensity of cells treated with different concentrations of FSK. (E) Quantification of ciliary pVASP intensity normalized to ciliary GFP intensity cells treated with FSK for different durations. Significance was determined via one-way ANOVA followed by Tukey’s multiple comparison test. (****p < 0.0001. Data are represented as means ± SD.) The underlying data for this figure are in S1 Data. (TIF) [file pbio.3003841.s001.tif]

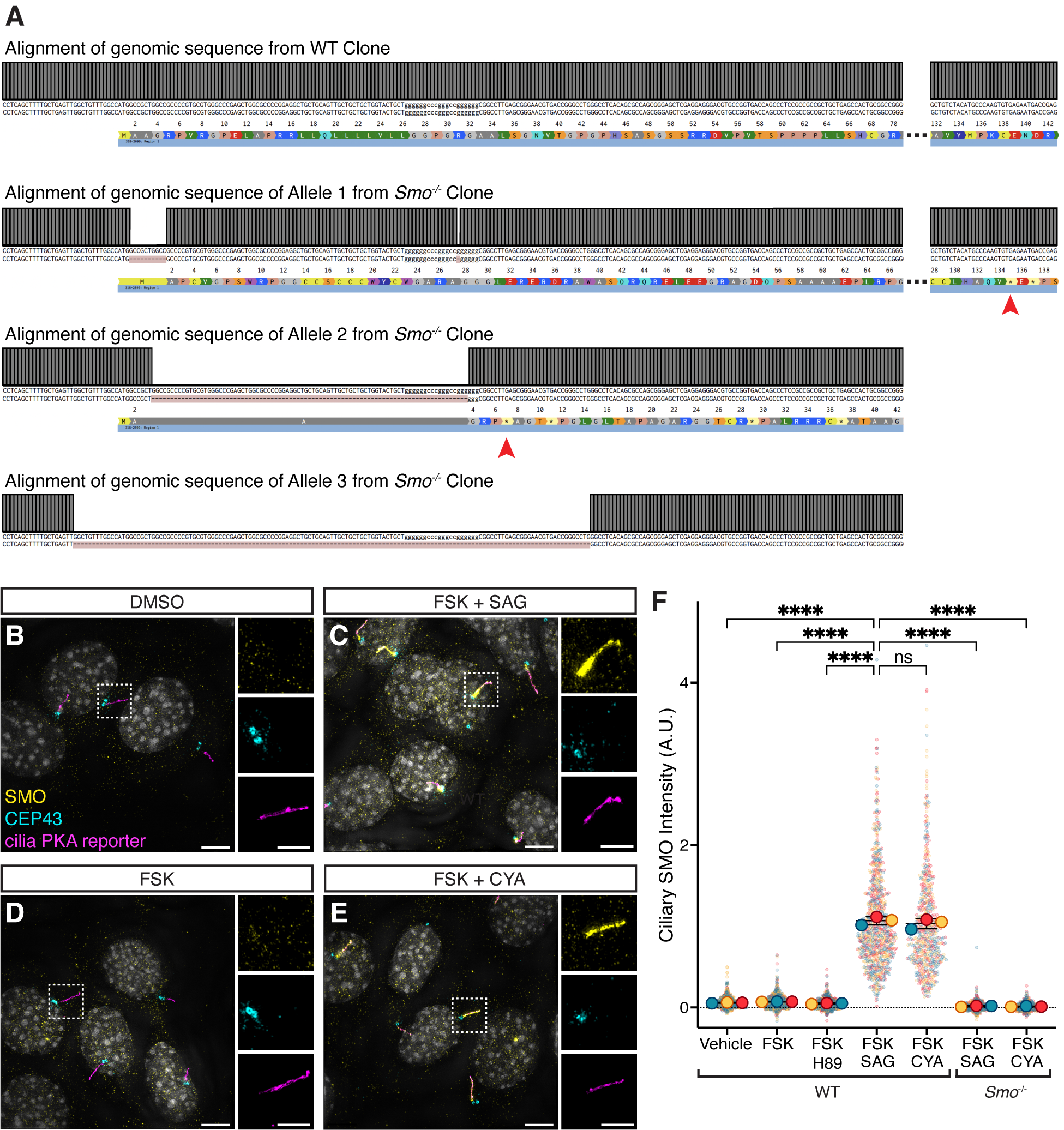

Supplement: S2 Fig — (A) Clustal Omega alignment of Sanger sequencing of the genomic sequence from Smo+/+ (WT) cilia PKA reporter clone, as well as Smo−/− cilia PKA reporter clone. NIH/3T3 cells are hypertriploid. The Smo−/− cilia PKA reporter clone has 3 different alleles. Allele 1 has a 10 bp and 1 bp deletion allele resulting in an early frameshift and early stop codon. Allele 2 has an 85 bp deletion resulting in an early frameshift and an early stop codon. Allele 3 has a 139 bp deletion that deletes the start codon. Stop codons are indicated with red arrows. Visualization adapted from Benchling. (B–E) Immunofluorescence images of cilia PKA reporter cells treated with the same regimes as in Fig 3B–3D, 3G. Images depict cells stained for SMO (SMO, yellow), basal bodies (CEP43, cyan), cilia PKA reporter (GFP, magenta), and nuclei (Hoechst, gray). Scale bars for larger images, 5 μm. Scale bars for insets are 2.5 μm. (F) Quantification of ciliary SMO localization from A–D. For all plots, each biological replicate is color-coded. Significance was determined via one-way ANOVA of the means of each biological replicate, followed by Šídák’s multiple comparison test. ****p < 0.0001. Data are represented as means of replicates ± SD. The underlying data for this figure are in S3 Data. (TIF) [file pbio.3003841.s002.tif]

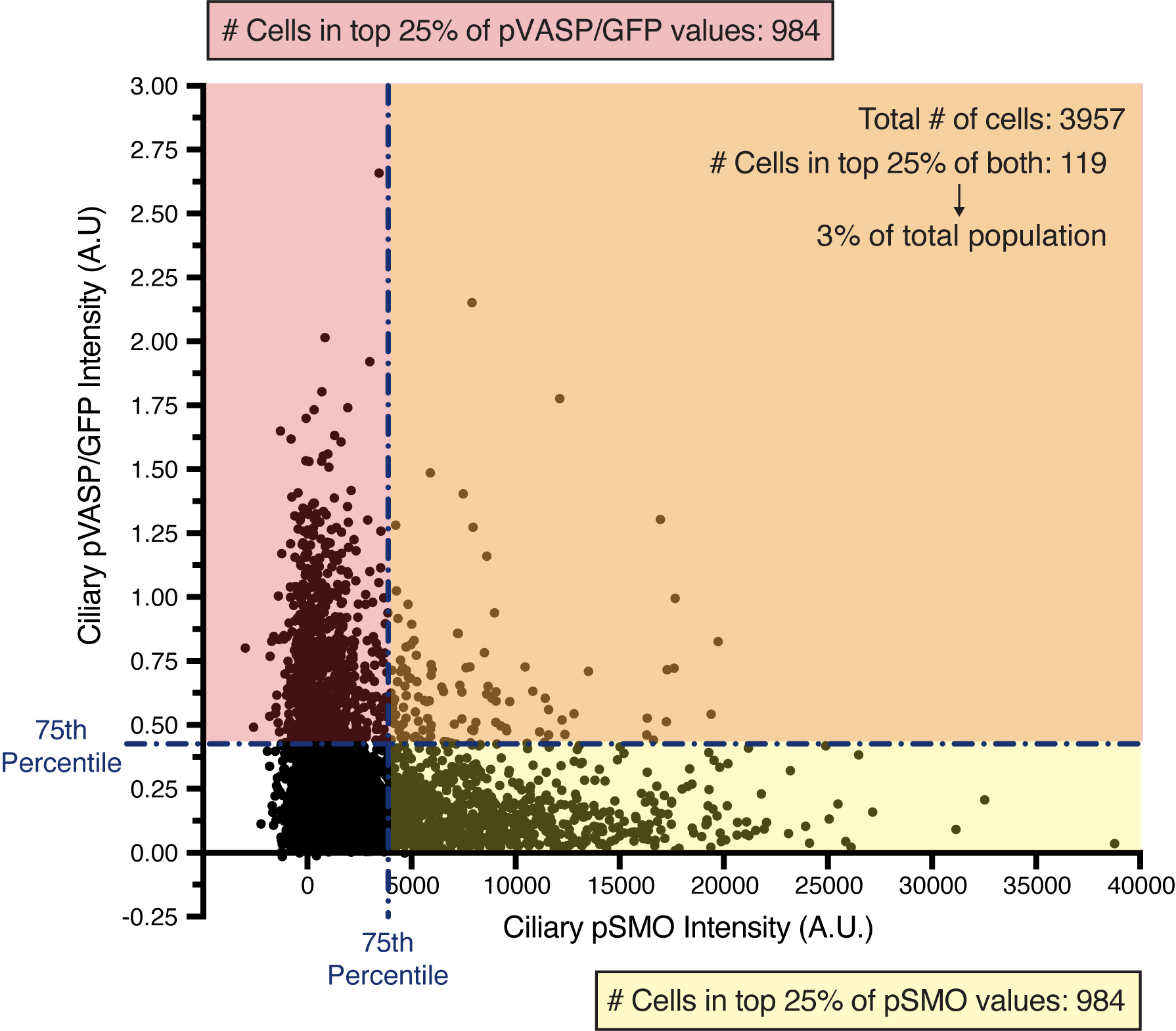

Supplement: S3 Fig — Each dot represents a cilium of a cilia PKA reporter cell treated with 15 min of 75 nM FSK, or SAG (1 nM, 2 nM, 5 nM, 10 nM or 50 nM) for 24 h followed by 15 min of 75 nM FSK. These data are also used in Fig 3F, 3G. The x-axis represents the level of ciliary pSMO in each cilium, and the y-axis represents the level of ciliary pVASP/GFP in that same cilium. The underlying data for this figure is in S4 Data. (TIF) [file pbio.3003841.s003.tif]

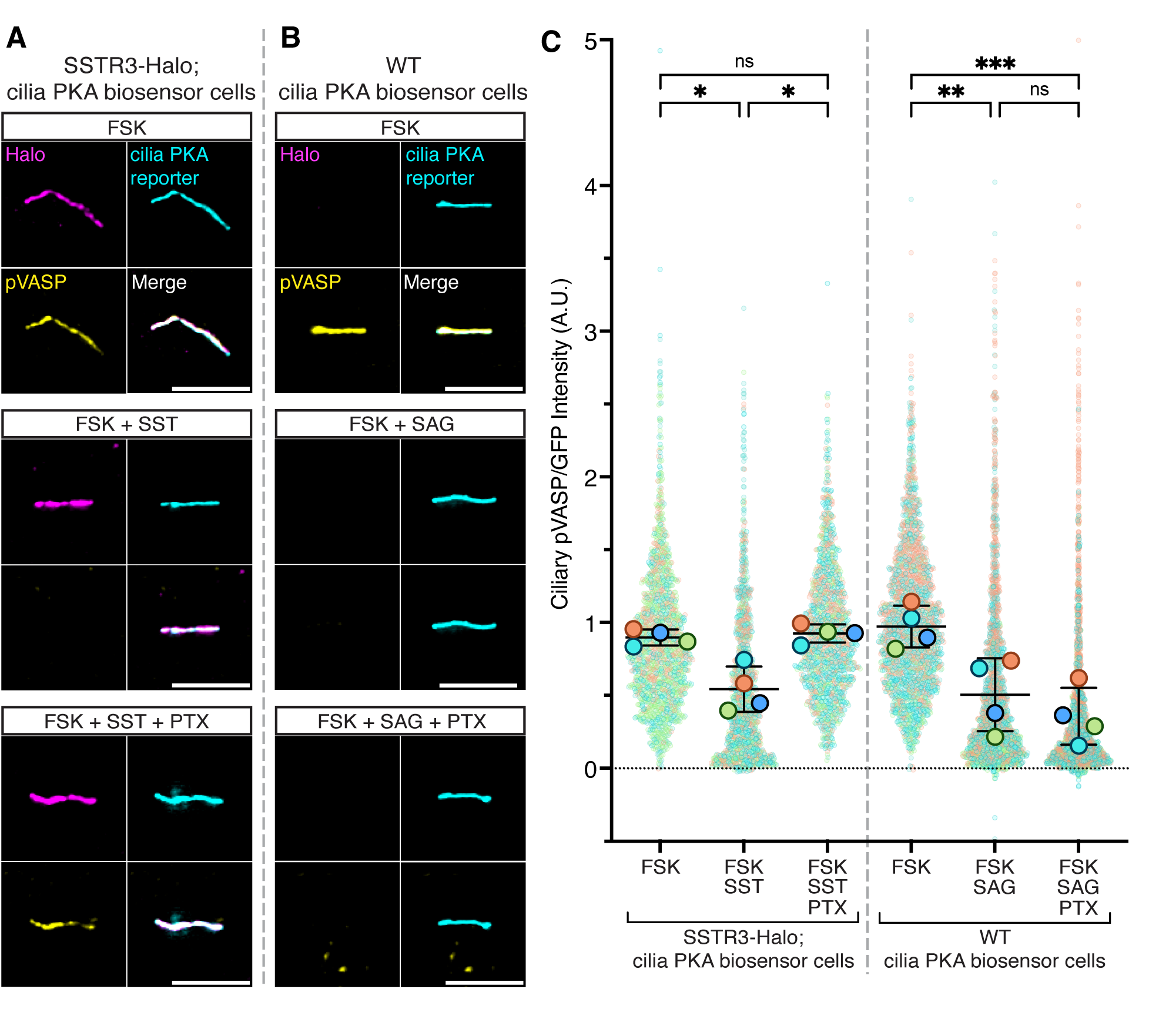

Supplement: S4 Fig — (A) Immunofluorescence imaging of cilia PKA reporter cells stably expressing Halo-tagged SSTR3 in response to doxycycline. Cells were serum-starved and treated with either FSK (100 nM for 15 min), SST and FSK (10 µM for 2 h and 100 nM for 15 min, respectively), or SST, PTX, and FSK (10 µM for 2 h, 100 ng/mL for 16 h, and 100 nM for 15 min, respectively). Images depict cells stained for pVASP (pVASPS157, yellow), cilia PKA reporter (GFP, cyan), and Halo (Halo, magenta). Scale bars are 5 µm. (B) Immunofluorescence imaging of cilia PKA reporter cells, performed as in A. Cells were serum-starved and treated with either FSK (100 nM for 15 min), SAG and FSK (100 nM for 24 h and 100 nM for 15 min, respectively), or SAG, PTX, and FSK (100 nM for 24 h, 100 ng/mL for 16 h, or 100 nM for 15 min, respectively). Scale bars are 5 µm. (C) Quantification of ciliary pVASP intensity, normalized to ciliary GFP, of A and B. Distinct biological replicate are represented with distinct colors. Significance was determined via one-way ANOVA of the means of each biological replicate, followed by Šídák’s multiple comparison test. P values are indicated as follows: **p < 0.003, ***p < 0.0002, and ****p < 0.0001. Data are represented as means of replicates ± SD. The underlying data for this figure is in S5 Data. (TIF) [file pbio.3003841.s004.tif]

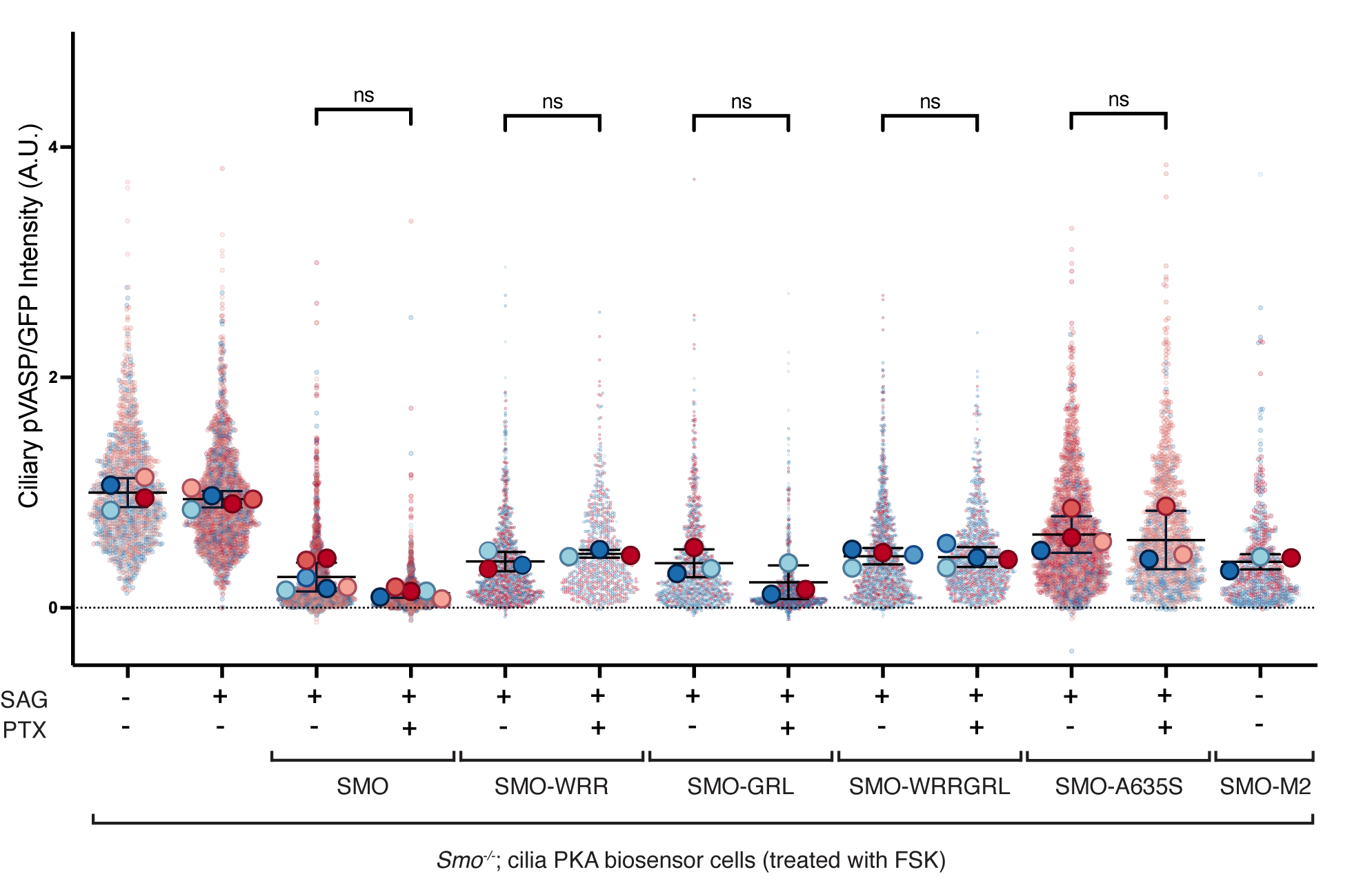

Supplement: S5 Fig — Quantification of ciliary pVASP intensity, normalized to ciliary GFP, in Smo−/− cilia PKA reporter cells expressing wild-type SMO, SMO-WRR, SMO-GRL, SMO-WRRGRL, SMO-A635S, or SMO-M2, as indicated. As indicated, cells were treated SAG (100 nM for 24 h), or SAG and PTX (100 nM for 24 h and 100 ng/mL for 16 h, respectively). All conditions were treated with FSK (100 nM for 15 min). For all plots, each biological replicate is color-coded. Same data as used in Fig 6. Significance was determined via one-way ANOVA of the means of each biological replicate, followed by Šídák’s multiple comparison test. P values are indicated as follows: **p < 0.003, ***p < 0.0002, and ****p < 0.0001. Data are represented as means of replicates ± SD. The underlying data for this figure is in S6 Data. (TIF) [file pbio.3003841.s005.tif]

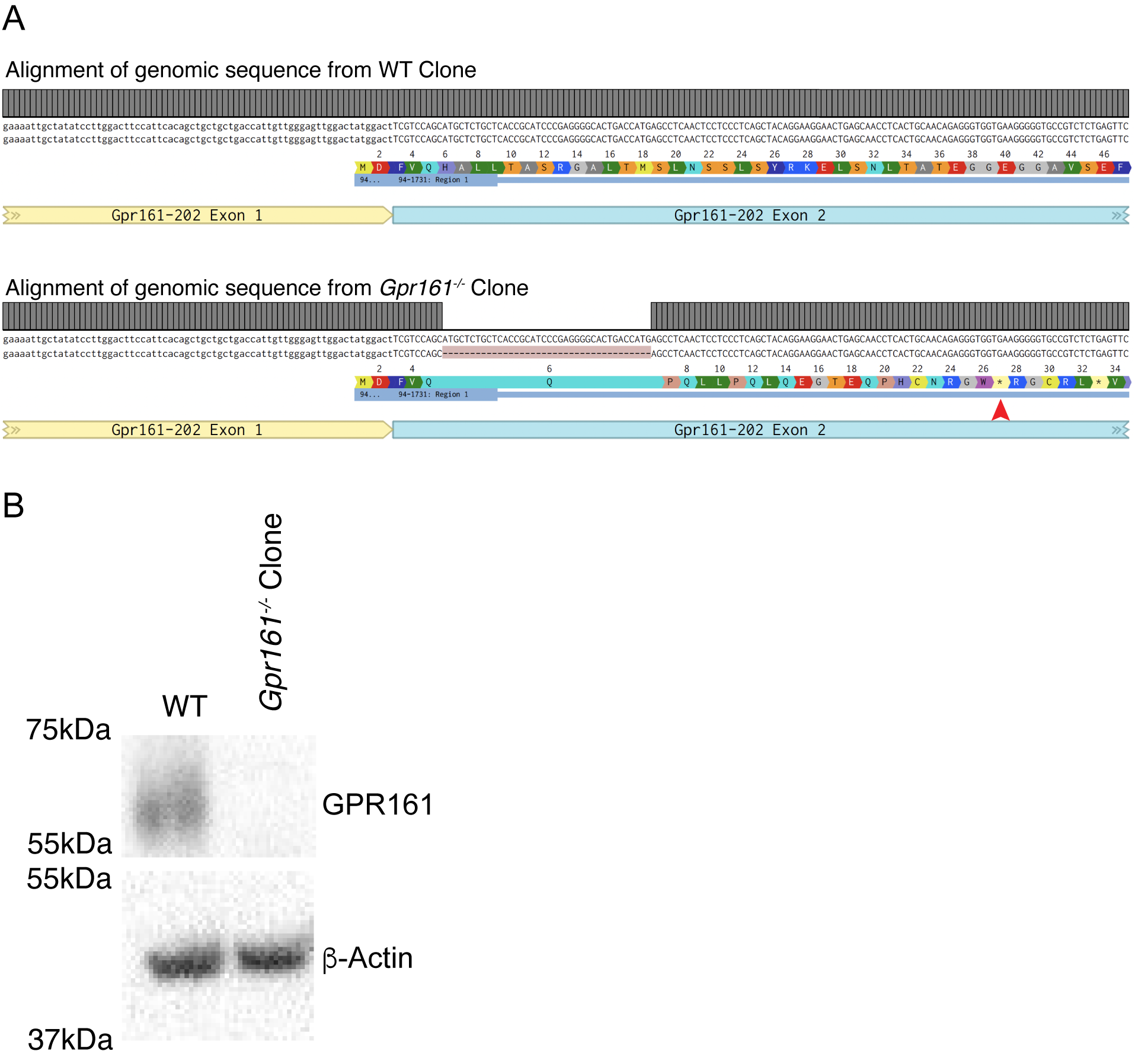

Supplement: S6 Fig — (A) Clustal Omega alignment of Sanger sequencing of the genomic sequence from Gpr161+/+ (WT) cilia PKA reporter clone, as well as Gpr161−/− cilia PKA reporter clone. The Gpr161−/− cilia PKA reporter clone has a 38 bp deletion resulting in an early frameshift and an early stop codon, indicated by the red arrow. Visualization adapted from Benchling. (B) Immunoblot of lysates from cilia PKA reporter cells and Gpr161−/− cilia PKA reporter cells. GPR161 is 60kDa. Blotting for β-actin controls for loading. (TIF) [file pbio.3003841.s006.tif]
