# Supplementary material for: Smoothened and ciliary GPCRs regulate ciliary protein kinase A activity involved in Hedgehog signal transduction
Source: PLoS Biol. 2026 Jun 10;24(6):e3003841. doi: 10.1371/journal.pbio.3003841 (PMC13289941; doi:10.1371/journal.pbio.3003841)
Supplement: S1 Raw Images — (Page 1) Immunoblot of lysates from cilia PKA reporter cells and Gpr161−/− cilia PKA reporter cells with rabbit anti-GPR161 from Saikat Mukhopadhyay. (Page 2) Merge of the brightfield ladder and rabbit anti-GPR161 immunoblot. (Page 3) Immunoblot of lysates from cilia PKA reporter cells and Gpr161−/− cilia PKA reporter cells with anti-β-actin as a loading control. (PDF) [file pbio.3003841.s007.pdf]

Rabbit anti-Gpr161 from Mukhopadhyay Lab

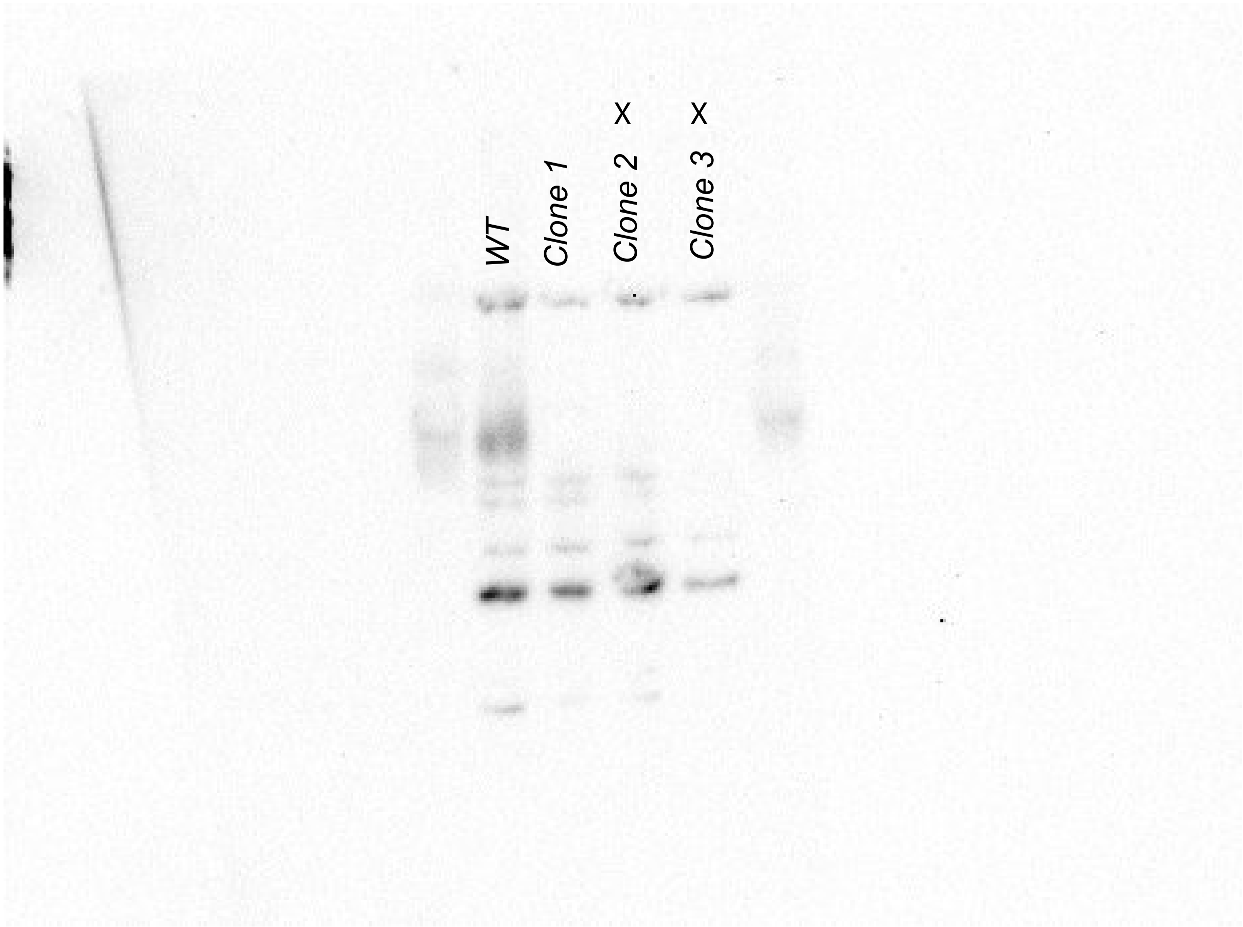

Raw Western blot from S6B.

Merge of Brightfield Ladder + Rabbit anti-Gpr161

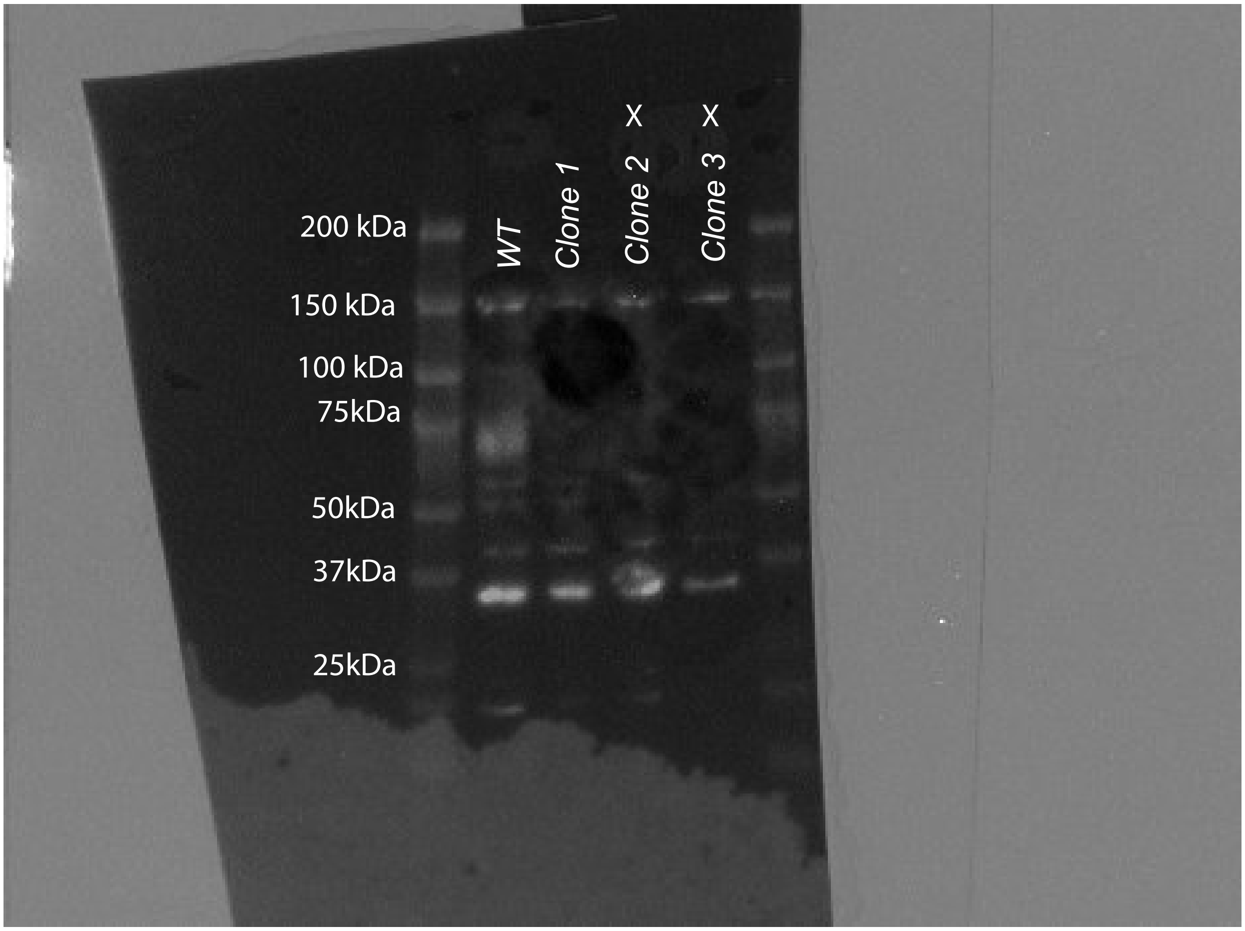

Proteintech ms Beta-Actin

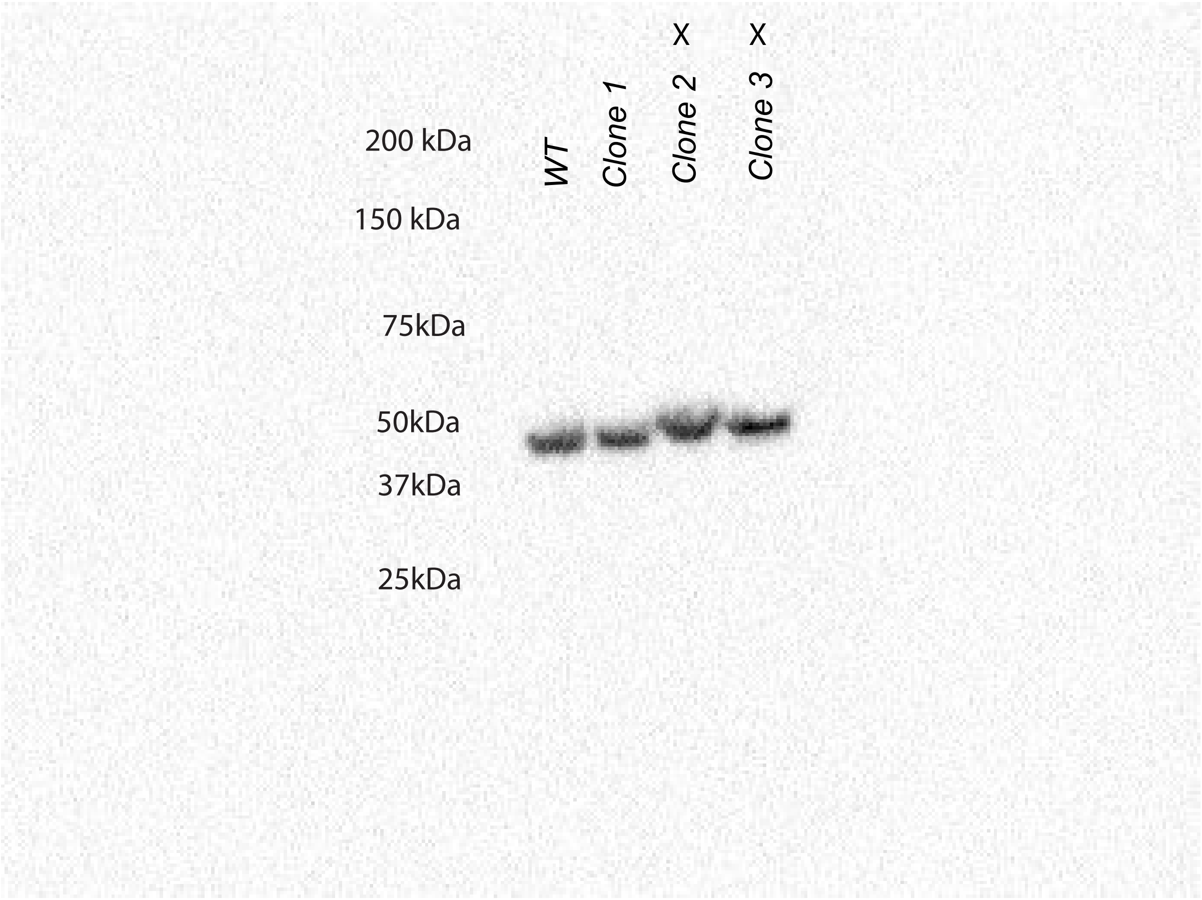

Raw Western blot from S6B.
