# Supplementary material for: Smoothened and ciliary GPCRs regulate ciliary protein kinase A activity involved in Hedgehog signal transduction
Source: PLoS Biol. 2026 Jun 10;24(6):e3003841. doi: 10.1371/journal.pbio.3003841 (PMC13289941; doi:10.1371/journal.pbio.3003841)
Supplement: Attachment — Submitted filename: Response to Reviewers Nguyen et al.pdf [file pbio.3003841.s017.pdf]

University of California
San Francisco

UCSF

Department of Biochemistry and Biophysics

JEREMY.REITER@UCSF.EDU
PHONE: (415) 502-8528
FAX: (415) 476-0526

SMITH CARDIOVASCULAR RESEARCH BUILDING
ROOM 352G, 555 MISSION BAY BLVD. SOUTH
SAN FRANCISCO, CA 94158-9001

Jeremy Reiter, M.D., Ph.D.
Professor

November 11, 2025

Dear Reviewers,

We thank you for your extremely helpful questions and comments. We deeply appreciate your thoughtful suggestions as they have led to extensive new data that have strengthened and extended the scope of the manuscript. We were encouraged to read that you found that the "work is well presented" (Reviewer #1), that the biosensor is "a valuable… powerful tool for the community" and that this work "provides additional mechanistic insight that will be of interest to the readership investigating Hedgehog signal transduction, primary cilia, and PKA signaling" (Reviewer #2), and that the work is "well-performed experimentally and well presented" (Reviewer #3).

After consultation with the editor, we have spent the last few months generating new data and revising the manuscript to strengthen the conclusions and address your concerns. The new data are presented as revisions to several figures (**Fig. S1, Fig. 3I, Fig. S5, Fig. 6, Fig. S6**) and the addition of new figures (**Fig. 7, Fig. S7, and Fig. 8**).

Our point-by-point response to your comments are below in blue.

Reviewers' comments

Reviewer 1:
In this manuscript, Nguyen and colleagues develop a ciliary PKA sensor by targeting the PKA phosphorylation site of VASP to cilia via Arl13b. They then use this reporter to examine how various perturbations to the Hedgehog pathway alter the phosphorylation of this reporter. The work is well presented with useful diagrams showing what aspect is being tested at each point. Overall, I do not have major concerns with the data. However, the discussion is pretty minimal and does not do a good job of explaining the work. I suggest that a version of the pathway diagram be added to the discussion with details of what was established in the work.

Thank you for your suggestion. We have now expanded the discussion and, as you recommended, created a new pathway diagram within the Discussion section (the new Figure 8) which highlights how ciliary PKA activity is regulated and controls downstream HH pathway activity. The figure includes a numbered list of points that we explore in the revised and deepened discussion.

In addition, the authors need to supply information about the antibodies used, and the characterization of critical antibodies. In particular, the sources and verification of phospho VAMP and phospho Smo antibodies are needed.

We appreciate the reviewer pointing out our error: we had forgotten to include the catalog numbers for the primary antibodies that we used. We have included the following information about the antibodies used in this work in the Methods section:

Anti-VASP (phospho S157) antibody [5C6] (Abcam, ab58555)
Anti-SMO (phospho pS594/pT597/pS599) antibody (7TM, 7TM0239A-IC)
Anti-HaloTag antibody (Promega, G9281)
Anti-Acetyl-a-Tubulin Lys40 (Cell Signaling Technology, 5335)
Anti-GFP VHH/Nanobody conjugated to Alexa Fluor 488 (ChromoTek, gb2AF488)

Reviewer 2:
In this manuscript, Nguyen and colleagues investigated the molecular mechanism of Hedgehog signal transduction in the primary cilium, a crucial process during embryonic development with important implications in Hedgehog-driven cancers and other cilia-dependent disorders. More specifically, the authors investigated how the oncogene Smoothened (SMO) inhibits protein kinase A (PKA) activity in the primary cilium. To do so, they developed a ciliary PKA reporter that assesses the PKA signaling status in primary cilia after cell or tissue fixation. In the presented work, the authors confirm the previously reported mode of PKA inhibition by SMO through a protein kinase inhibitor motif in the C-terminal tail, which requires GRK2/3 phosphorylation. While the gist of this mechanism has been described by Walker et al. (in PLoS Biol, 2024), the authors provide additional mechanistic insight that will be of interest to the readership investigating Hedgehog signal transduction, primary cilia, and PKA signaling.

The main conclusions of the manuscript agree with the current literature and are therefore supported not only by the presented data but also previous work. Intriguingly, the authors gained additional insight in the regulation of PKA activity in primary cilia, as SMO appears to block PKA activity independent of G proteins. While this has been suggested in previous work (Walker et al., 2024) the supporting data was limited. Here, the authors provide additional supporting data for such a model, yet, the data is still not convincing, as the experimental setup does not justify the conclusion. However, based on previous publications the authors could design more adequate experiments to unambiguously confirm their hypothesis that PKA regulation in Hedgehog signaling may be independent of a putative G protein coupling of SMO.

While the developed PKA reporter will be a valuable tool for the community, it also has limitations, as it cannot follow the PKA activity in living cells and can only assess one point in time in the primary cilium. Therefore, to avoid confusion, I recommend the use of the word <reporter> instead of <sensor>, as the latter will be misleading for the readership studying signal transduction processes. Nevertheless, I want to re-iterate that this PKA reporter will be a powerful tool for the community, as it is compatible with fixation of specimens -in contrast to most biosensors.

Thank you for your helpful recommendation. Accordingly, we have changed all usage of the word <sensor> or <biosensor> to <reporter> within the manuscript.

The manuscript reports two main findings, 1) the development of a PKA activity reporter, and 2) SMO-mediated inactivation of PKA may be independent of G proteins. The second point requires additional work to warrant publication in its current form: The suggested model claims reduction of PKA activity by SMO when Hedgehog signaling is active. Therefore, one would assume that PKA activity is high when the pathway is inactive. However, the pVASP-based PKA reporter does not detect any PKA activity in unstimulated cells. How do the authors explain that they require a strong PKA activator, such as forskolin (FSK) to activate PKA in a seemingly unphysiological way in cell culture cilia? It is unclear whether FSK also needs to be added to see the in vivo differences in tissues (Fig. 2). Why this discrepancy? If the reporter lacks the sensitivity in cell culture to differentiate between non-stimulated (PKA active) and Hedgehog stimulated (PKA inactive) cilia in the absence of FSK, the resulting

interpretations stand on a weak foundation. How do the authors explain that activation of SMO by SAG antagonizes the unphysiological FSK-mediated increase in PKA activity in cilia.

We apologize for our lack of clarity. To answer the question about whether the pVASP-based PKA reporter has sufficient sensitivity to detect differences in ciliary PKA activity in the absence of FSK, we conducted additional experiments. As alluded to by reviewer 2, the zebrafish embryo experiments performed in the absence of FSK suggested that the PKA reporter detects PKA activity in cells not stimulated by FSK (Figure 2). In the revised manuscript, we include new data showing that baseline ciliary PKA activity in unstimulated cultured cells is detectable using the pVASP-based reporter, and that this baseline ciliary PKA activity is repressed by activation of SMO. We include these new data in a new figure panel (Figure 3I). Inspired by historical approaches to assaying PKA inhibition (such as assessment of $G\alpha_{i/o}$-coupled GPCRs which have typically been conducted under conditions of cAMP stimulation, often with FSK), we included FSK in some experiments to activate adenylyl cyclases (*1*, *2*). For many assays measuring PKA inhibition, including our own, conditions of cAMP stimulation increase the sensitivity of the assay by increasing the dynamic range (*3*–*6*).

The control experiment that this is SMO dependent (Fig 3G) is convincing, but it is unclear whether this is a cilium-specific effect. As FSK activates PKA in the entire cell (not only in cilia) SAG treatment may reverse the non-cilia PKA activity. The same might be true for inhibiting PKA activity by stimulating SSTR3. As these processes seem very difficult to dissect, the authors should at least provide alternative explanations for these findings, as they cannot rule out cilia-independent effects.

Thank you for this important insight. Indeed, it is extremely difficult to prove that any effect is entirely cilium-specific. As in Figure 3B-G, we are measuring activation of the cilium localized PKA reporter at the cilium (defined by costaining for ciliary marker acetylated tubulin). Thus, we conclude that SMO and SSTR3 inhibit PKA activity at the cilium and, as suggested, pointed out by the reviewer, may also inhibit PKA elsewhere. We now explicitly comment on this possibility in the revised, expanded Discussion. We write: "One limitation of the ciliary PKA reporter is that it does not assess PKA activity outside of the cilium. Thus, it remains unclear whether SMO or ciliary GPCRs also affect PKA activity outside of the cilium."

* Whether SMO is indeed a G-I-coupled GPCR appears to be a long-standing controversy in this field. One key experiment of this study (Fig.5) shows that the inhibition of the PKA activity by a cilia-localized SSTR3 receptor can be prevented by pertussis toxin (PTX), which blocks the G-I-mediated signaling cascade. In contrast, PTX treatment does not prevent blocking the PKA activity by SMO/SAG, which suggests that this effect is not mediated by G-I. However, the way how this experiment was performed does not allow drawing this conclusion. PTX works by blocking G-I, which itself blocks adenylate cyclases to lower cAMP production. For PTX to show any effect, requires an -independent- activation of the adenylate cyclases, usually by other stimulating G-S coupled GPCRs. One important adenylate cyclase stimulating GPCR in the Hedgehog signaling pathway is GPR161, which is removed from cilia shortly after Hedgehog induction (Mukhopadhyay, 2013). The experiment in Fig.5 was performed by adding SAG for 24 hours, followed by PTX treatment for 16 hours. Under these conditions the stimulating GPR161 was removed long before the PTX treatment started. Under these conditions, it cannot be expected that PTX increases cAMP levels in cilia as the only known stimulating GPCR is absent from cilia. Hence, on shorter timeframes or in more physiological concentration ranges (Hedgehog morphogens are highly concentration dependent in vivo), it cannot be ruled out that SMO may mediate its function via G-I coupling. One potential way to address this, would be a short-term SMO activation when increased levels of cAMP are present in cilia, which may be provided by FSK or more cilium-specific cAMP production, such as by optogenetics, which the authors have nicely done in previous studies (Truong et al., 2021).

We were inspired by your idea that GPR161 is likely to be an important adenylate cyclase-stimulating GPCR that could be opposed by SMO-coupled $G\alpha_i$ to control ciliary PKA activity. To test this possibility,

we knocked out GPR161 in NIH-3T3 cells. Surprisingly, we found that ciliary PKA activity was still elevated over baseline in the absence of GPR161. These new data are included in the new Figure 7A.

One possibility raised by these findings is that $G\alpha_s$-coupled GPCRs other than GPR161 stimulate ciliary PKA. Candidates for these additional $G\alpha_s$-coupled GPCRs include GPR175 (*7*), a ciliary GPCR studied by Dr. Suzie Scales, and GPR75, a recently described ciliary GPCR that we have found is expressed in fibroblasts (*8*).

Because baseline ciliary PKA activity was not dependent on GPR161, we did not pursue identification of a time window in which SMO was ciliary and activated but ciliary GPR161 had not yet decreased to assess whether $G\alpha_i$ would be important for SMO-mediated ciliary PKA inhibition specifically during this window. Rather, we followed your suggestion to test PTX in the presence of an independent activation of the adenylate cyclases by independently activating adenylate cyclases with FSK. We took inspiration from previous assays for PTX-mediated $G\alpha_i$-coupled GPCRs in the presence of FSK-mediated activation of adenylate cyclases (*1*, *9*–*14*, *2*, *15*–*20*).In the presence of FSK, we found that PTX blocked the ability of SSTR3 to inhibit ciliary PKA activity. In the presence of FSK, PTX did not block the ability of SMO to inhibit ciliary PKA activity. We have included these additional data in the new Figure S5. We concur with the point that in more physiological conditions or in other time windows, SMO may signal via $G\alpha_i$-coupling. We discuss this possibility in the revised and expanded Discussion: "NIH-3T3 cells are unlikely to faithfully model HH signal transduction in all cell types. Therefore, it is possible that $G\alpha_i$-coupling is a mechanism by which SMO inhibits ciliary PKA activity in other cell types."

To further strengthen the manuscript, I have the following suggestions: * A central new finding of this study that PKA activity in cilia is modulated independently of G proteins suggests that it may be independent of cAMP levels. However, this contrasts with their previous, very prominent publication (Truong et al., 2021), in which they show that Hedgehog signaling can be modulated by locally modulating the cAMP levels. The authors should clarify this difference for the non-specialists.

As suggested, we have clarified this difference by highlighting that ciliary cAMP levels are sufficient to inhibit Hedgehog signal transduction and that SMO can inhibit PKA independently of cAMP levels. We emphasize this clarification in the Discussion: "Thus, while SMO inhibits ciliary PKA independent of $G\alpha_i$, cAMP-mediated activation of ciliary PKA is sufficient to repress the HH signal transduction pathway.".

* As the developed PKA reporter is at the heart of this manuscript, a more thorough description would be helpful for a better assessment by the reader. For example, the authors should provide lower magnification images in cell culture, such that the localization and specificity of the signals can be evaluated.

Please see Supplemental Figure 1 for representative lower-magnification images of cultured cells.

Along the same line, the authors overstate findings obtained with their PKA reporter. For example, they conclude that they "produced a half-maximal increase in cilia PKA" activity. Such a specific statement can hardly be made from three concentrations investigated (Fig. S1D).

Thank you for the helpful feedback. We have now conducted a more robust dose-response curve of the PKA reporter over 10 concentrations (the new Figure S1D). Additionally, we edited the text of the Results to read "produced an intermediate increase in cilia PKA" activity, recognizing that we cannot conclusively identify the half-maximal ciliary PKA activity as the assay is unlikely to be perfectly sensitive.

Also, the authors did not determine any enzymatic activity of PKA, so to not confuse experts on molecular signaling, the statement in the discussion should be re-phrased (page28).

As suggested, we changed the statement in the discussion from “measuring ciliary PKA activity” to “measuring ciliary PKA reporter activity.”

* Similarly, while I appreciate the experiment presented in Fig.S4 that only very few cilia with pSMO and pVASP signals could be found by the authors, the "mutually exclusiveness" criterion is not met. I recommend re-phrasing or providing additional data.

We agree with your recommendation and therefore changed “mutually exclusive” to “negatively correlated.”

* The use of the PKA reporter to investigate different tissues in zebrafish embryos is a great achievement, however, the LUT chosen to display the results is misleading. The overlap of magenta and green should be white, yet, white is part of the magenta-based LUT displaying pVAST (displayed in Fig. 2), such that it is unclear how much of the overlap in the insets is indeed from an overlap or the high intensity of the pVASP signal.

Thank you for pointing out this unintentionally confusing LUT use. To avoid this lack of clarity, we changed the LUT from magenta-hot (which includes white) to magenta (which does not include white).

* The authors nicely show that by converting the SMO C-tail from a pseudosubstrate to a substrate of PKA it ceases to block PKA activity. They further propose in the discussion that this should increase its dissociation rate, which seems like the most plausible mechanism. While not essential, the authors should consider generating such data to further confirm this proposed mechanism.

We have spent the last few months attempting to quantitate the dissociation rate of PKA-C from wild-type SMO (PKA pseudosubstrate) and SMO-A635S (PKA substrate). We have met with technical difficulties in isolating sufficient recombinant proteins for these assays. As Thi is graduating and the reviewer deems this experiment not essential, a postdoc in the lab, Mia Konjikusic will continue to work on these experiments for inclusion in a follow-up paper, if that meets with the reviewer’s approval.

Minor points:

* Page2: G proteins that couple to GPCRs are not the "small" GTPases but "trimeric" GTPases by common nomenclature.
* Pages9/10: panel numbering of the figure seems off.
* Page10: The test for linear trend should be specified.
* Scale bars should represent micrometers not micromolar concentrations.
* Hoescht dye is misspelled.
* Page15: bottom, "induced induce" should be fixed.
* Page19: concentration of SAG for the experiments in Fig S4 should be provided.
* Page23: SMO-M2 mutation contains error in amino acid exchange.

We made each of these changes. We deeply appreciate your careful attention to this work.

Rev. 3:

Nguyen et al examines the relationship between SMO and PKA within primary cilia. Specifically, they are building upon their findings that the ciliary pool of PKA and not the cytoplasmic pool is specifically inhibits activation of HH signaling, and also extending recent findings, primarily from the Myers lab, that SMO primarily activates HH signaling at the cilium by binding to and inactivating PKA. The authors develop a PKA biosensor consisting of a PKA-specific substrate motif (from VASP) added to GFP and targeted to cilia by fusion to Arl13b. The phosphorylated motif is then recognized by a specific phospho-VASP antibody. They then demonstrate that this biosensor detects graded PKA activity in zebrafish somite development that corresponds to graded HH signaling in that tissue. They then use this biosensor to interrogate how PKA is regulated by SMO within cilia. I generally found this work to be well-performed experimentally and well presented. My main critique is that it represents only a modest

advance in our mechanistic understanding of SHH pathway regulation in the cilium, relative to prior work (for example: Arveseth et al., 2021; Happ et al., 2022; Walker et al., 2024). The biosensor is and important tool that has enabled confirmation of prior findings with spatial resolution. This certainly has value, but may not be right for publication in PLoS Biology absent substantial additional findings that provide genuinely new mechanistic insights.

Thank you for your helpful comments. We have spent the last few months working to contribute additional findings that provide new mechanistic insights to this manuscript. Most of the new additional data are included in Figures 5, S5, 6, S6, 7 and S7 of the revised manuscript.

We have sought to address the question of how SMO communicates to the GLI proteins to drive HH signaling, focusing on how ciliary PKA is regulated and whether this regulation happens within the primary cilium. The existing literature discusses three ways by which SMO can inhibit PKA to release GLI-mediated induction of the HH transcriptional program. The previous version of our manuscript focused on distinguishing between two of these mechanisms:

1) SMO signals through $G\alpha_{i/o}$ to decrease cAMP concentrations to inhibit PKA (*21–23*)
2) SMO directly binds and sequesters PKA-C to inactivate its kinase activity (*24–26*)

We have strengthened these data (found principally in the revised Figures 5, S5, 6, S6 and discussed above in comments to reviewer 2). These results indicate that the second mechanism, but not the first, contributes to SMO-mediated control of ciliary PKA activity.

The pioneering work of Happ et al. demonstrated that SMO can bind and inhibit PKA. The spatial resolution provided by the ciliary PKA activity reporter provides insight into where within the cell the Hedgehog signal transduction pathway regulates PKA. The additional experiments with SSTR3, a cilia-localized $Ga_{i/o}$-coupled GPCR, extend these results beyond the Hedgehog pathway to reveal that ciliary GPCRs also regulate ciliary PKA activity, but via a distinct mechanism. These findings help to explain how ciliary cAMP and ciliary GPCRs can regulate the Hedgehog transcriptional program, now discussed in the revised Discussion section.

To these insights, we now add investigation of the function of the SMO PKI-like domains and their ability to inhibit ciliary PKA (included in the revised **Figure 6 and S6**). Happ et al. identified that mutation of three residues in one PKI-like domain (W622A, R632A, R633A, referred to as WRR) is sufficient to prevent SMO signaling. Surprisingly, we found that SMO WRR still inhibits cilia PKA reporter activity equivalently to wild-type SMO (**Figure 6B-C**). But, consistent with the previous findings, we find that SMO-WRR is impaired in the activation of the Hedgehog transcriptional response (**Figure 6D**). Together with additional data included in the revised manuscript, these unexpected results suggest that the inability of SMO WRR to transduce HH signaling is due to a mechanism distinct from ciliary localization, GRK2-mediated phosphorylation or ciliary PKA inhibition, a finding that opens new areas of investigation that we are pursuing and expect will inspire further advances from others in the field.

In a further effort to address the request for additional mechanistic insights, we have also extended our analysis to the third mechanism:

3) GPR161 is a $G\alpha_s$-coupled GPCR that increases cAMP in the cilium to activate PKA. Upon activation of SMO, SMO drives exit of GPR161 from the cilium thereby decreasing cAMP in the cilium to inhibit PKA (*27–29*)

Most of these data are found in the entirely new Figure 7 and S7. This work investigates the mechanisms by which baseline ciliary PKA activity keeps HH signaling off and tests the assertion, made in several papers and reviews, that GPR161 acts at the cilium to activate PKA and keep the HH signal transduction pathway off in the absence of ligand(*27*, *28*, *30*–*33*). Unexpectedly, we found that ablation of *Gpr161* did not reduce baseline ciliary PKA activity. One possibility, discussed above in comments for reviewer 2, is that other GPCRs activate ciliary PKA in the absence of HH signaling. Alternatively, a GPCR-independent mechanism may maintain ciliary PKA activity.

To further test how GPR161 may participate in ciliary HH signal transduction, we tested whether GPR161 may regulate ciliary PKA through its AKAP domain (as suggested by (*34*, *35*)). We found that mutation of the GPR161 AKAP domain prevented recruitment of PKA-R to the primary cilium (Figure 7C-D). Decreased recruitment of PKA-R was, surprisingly, associated with reduced ciliary PKA activity (Figure 7A-B). We then examined PKA-C and found that wild-type GPR161 recruits both PKA-R and PKA-C to cilia in a way that depends on the GPR161 AKAP domain. In previously described cellular contexts, PKA-R *inhibits* PKA-C activity (*36*–*38*). In contrast, our results suggest that, in the cilium, GPR161 binding to PKA-R is critical for ciliary localization of PKA-C, thereby acting to *promote* ciliary PKA-C activity. Thus, these new results change the model by which GPR161 inhibits HH signal transduction. Instead of acting through Ga_s to increase cAMP, these results suggest that GPR161 recruits PKA to the primary cilium. The implications of these findings are now also considered in the revised Discussion section.

Reviewers, thank you for your extensive help with this manuscript. Your efforts and suggestions have substantially improved our work, and we deeply appreciate the opportunity to submit a revised manuscript to you. Please let us know if we can provide you with any additional information or answer any further questions you might have.

Sincerely,

Jeremy Reiter, MD, PhD
Professor
Department of Biochemistry and Biophysics
University of California, San Francisco

References

1. K. B. Seamon, W. Padgett, J. W. Daly, Forskolin: unique diterpene activator of adenylate cyclase in membranes and in intact cells. *Proc Natl Acad Sci U S A* **78**, 3363–3367 (1981).

2. T. Wang, Z. Li, M. E. Cvijic, L. Zhang, C. S. Sum, "Measurement of cAMP for Gαs- and Gαi Protein-Coupled Receptors (GPCRs)" in *Assay Guidance Manual*, S. Markossian, A. Grossman, M. Arkin, D. Auld, C. Austin, J. Baell, K. Brimacombe, T. D. Y. Chung, N. P. Coussens, J. L. Dahlin, V. Devanarayan, T. L. Foley, M. Glicksman, K. Gorshkov, J. V. Haas, M. D. Hall, S. Hoare, J. Inglese, P. W. Iversen, M. Lal-Nag, Z. Li, J. R. Manro, J. McGee, O. McManus, M. Pearson, T. Riss, P. Saradjian, G. S. Sittampalam, M. Tarselli, O. J. Trask, J. R. Weidner, M. J. Wildey, K. Wilson, M. Xia, X. Xu, Eds. (Eli Lilly & Company and the National Center for Advancing Translational Sciences, Bethesda (MD), 2004; http://www.ncbi.nlm.nih.gov/books/NBK464633/).

3. C. P. O'Banion, M. A. Priestman, R. M. Hughes, L. E. Herring, S. J. Capuzzi, D. S. Lawrence, Design and Profiling of a Subcellular Targeted Optogenetic cAMP-Dependent Protein Kinase. *Cell Chemical Biology* **25**, 100-109.e8 (2018).

4. C. P. O'Banion, B. M. Vickerman, L. Haar, D. S. Lawrence, Compartmentalized cAMP Generation by Engineered Photoactivated Adenylyl Cyclases. *Cell Chemical Biology* **26**, 1393-1406.e7 (2019).

5. B. L. Ross, B. Tenner, M. L. Markwardt, A. Zviman, G. Shi, J. P. Kerr, N. E. Snell, J. J. McFarland, J. R. Mauban, C. W. Ward, M. A. Rizzo, J. Zhang, Single-color, ratiometric biosensors for detecting signaling activities in live cells. *eLife* **7**, e35458 (2018).

6. C. I. Massengill, J. Day-Cooney, T. Mao, H. Zhong, Genetically encoded sensors towards imaging cAMP and PKA activity in vivo. *Journal of Neuroscience Methods* **362**, 109298 (2021).

7. J. Singh, X. Wen, S. J. Scales, The Orphan G Protein-coupled Receptor Gpr175 (Tpra40) Enhances Hedgehog Signaling by Modulating cAMP Levels. *Journal of Biological Chemistry* **290**, 29663–29675 (2015).

8. Y. Jiang, Y. Xun, Z. Zhang, Central regulation of feeding and body weight by ciliary GPR75. *Journal of Clinical Investigation* **134**, e182121 (2024).

9. T. D. Reisine, Y. Zhang, R. D. Sekura, Pertussis toxin blocks somatostatin's inhibition of stimulated cyclic AMP accumulation in anterior pituitary tumor cells. *Biochemical and Biophysical Research Communications* **115**, 794–799 (1983).

10. F. Okada, Y. Tokumitsu, Y. Nomura, Pertussis Toxin Attenuates 5-Hydroxytryptamine$_{1A}$ Receptor-Mediated Inhibition of Forskolin-Stimulated Adenylate Cyclase Activity in Rat Hippocampal Membranes. *Journal of Neurochemistry* **52**, 1566–1569 (1989).

11. E. R. Neptune, T. Iiri, H. R. Bourne, Gαi Is Not Required for Chemotaxis Mediated by Gi-coupled Receptors. *Journal of Biological Chemistry* **274**, 2824–2828 (1999).

12. F. J. Ehlert, G. W. Sawyer, E. E. Esqueda, Contractile role of M2 and M3 muscarinic receptors in gastrointestinal smooth muscle. *Life Sciences* **64**, 387–394 (1999).

13. N. Bevan, T. Palmer, T. Drmota, A. Wise, J. Coote, G. Milligan, S. Rees, Functional analysis of a human A_1 adenosine receptor/green fluorescent protein/G_{i1} α fusion protein following stable expression in CHO cells. *FEBS Letters* **462**, 61–65 (1999).

14. E. A. Thomas, J. R. Matli, J. L. Hu, M. J. Carson, J. G. Sutcliffe, Pertussis toxin treatment prevents 5-HT5a receptor-mediated inhibition of cyclic AMP accumulation in rat C6 glioma cells. *J. Neurosci. Res.* **61**, 75–81 (2000).

15. R. Mistry, M. R. Dowling, R. A. J. Challiss, An investigation of whether agonist-selective receptor conformations occur with respect to M_2 and M_4 muscarinic acetylcholine receptor signalling *via* $G_{i/o}$ and G_s proteins. *British J Pharmacology* **144**, 566–575 (2005).

16. R. Mostany, Á. Díaz, E. M. Valdizán, M. Rodríguez-Muñoz, J. Garzón, M. A. Hurlé, Supersensitivity to μ-opioid receptor-mediated inhibition of the adenylyl cyclase pathway involves pertussis toxin-resistant Gα protein subunits. *Neuropharmacology* **54**, 989–997 (2008).

17. O. M. Pello, L. Martínez-Muñoz, V. Parrillas, A. Serrano, J. M. Rodríguez-Frade, M. J. Toro, P. Lucas, M. Monterrubio, C. Martínez-A, M. Mellado, Ligand stabilization of CXCR4/δ-opioid receptor heterodimers reveals a mechanism for immune response regulation. *Eur J Immunol* **38**, 537–549 (2008).

18. A. C. Keen, M. H. Pedersen, L. Lemel, D. J. Scott, M. Canals, D. R. Littler, T. Beddoe, Y. Ono, L. Shi, A. Inoue, J. A. Javitch, J. R. Lane, OZITX, a pertussis toxin-like protein for occluding inhibitory G protein signalling including Gαz. *Commun Biol* **5**, 256 (2022).

19. M. J. Wall, E. Hill, R. Huckstepp, K. Barkan, G. Deganutti, M. Leuenberger, B. Preti, I. Winfield, S. Carvalho, A. Suchankova, H. Wei, D. Safitri, X. Huang, W. Imlach, C. La Mache, E. Dean, C. Hume, S. Hayward, J. Oliver, F.-Y. Zhao, D. Spanswick, C. A. Reynolds, M. Lochner, G. Ladds, B. G. Frenguelli, Selective activation of Gαob by an adenosine A1 receptor agonist elicits analgesia without cardiorespiratory depression. *Nat Commun* **13**, 4150 (2022).

20. S. M. Bagger, H. Schihada, A. L. S. Walser, A. K. Drzazga, L. Grätz, T. Palmisano, C. K. Kuhn, M. Mavri, A.-S. Mølleskov-Jensen, G. G. Tall, T. Schöneberg, S. J. Mathiasen, J. A. Javitch, G. Schulte, K. Spiess, M. M. Rosenkilde, Complex G-protein signaling of the adhesion GPCR, ADGRA3. *Journal of Biological Chemistry* **301**, 108441 (2025).

21. S. K. Ogden, D. L. Fei, N. S. Schilling, Y. F. Ahmed, J. Hwa, D. J. Robbins, G protein Gαi functions immediately downstream of Smoothened in Hedgehog signalling. *Nature* **456**, 967–970 (2008).

22. N. A. Riobo, B. Saucy, C. Dilizio, D. R. Manning, Activation of heterotrimeric G proteins by Smoothened. *Proc Natl Acad Sci U S A* **103**, 12607–12612 (2006).

23. F. Shen, L. Cheng, A. E. Douglas, N. A. Riobo, D. R. Manning, Smoothened is a fully competent activator of the heterotrimeric G protein G(i). *Mol Pharmacol* **83**, 691–697 (2013).

24. C. D. Arveseth, J. T. Happ, D. S. Hedeen, J.-F. Zhu, J. L. Capener, D. Klatt Shaw, I. Deshpande, J. Liang, J. Xu, S. L. Stubben, I. B. Nelson, M. F. Walker, K. Kawakami, A. Inoue, N. J. Krogan, D. J. Grunwald, R. Hüttenhain, A. Manglik, B. R. Myers, Smoothened transduces Hedgehog signals via activity-dependent sequestration of PKA catalytic subunits. *PLoS Biol* **19**, e3001191 (2021).

25. M. F. Walker, J. Zhang, W. Steiner, P.-I. Ku, J.-F. Zhu, Z. Michaelson, Y.-C. Yen, A. Lee, A. B. Long, M. J. Casey, A. Poddar, I. B. Nelson, C. D. Arveseth, F. Nagel, R. Clough, S. LaPotin, K. M. Kwan, S. Schulz, R. A. Stewart, J. J. G. Tesmer, T. Caspary, R. Subramanian, X. Ge, B. R. Myers, GRK2 kinases in the primary cilium initiate SMOOTHENED-PKA signaling in the Hedgehog cascade. *PLoS Biol* **22**, e3002685 (2024).

26. J. T. Happ, C. D. Arveseth, J. Bruystens, D. Bertinetti, I. B. Nelson, C. Olivieri, J. Zhang, D. S. Hedeen, J.-F. Zhu, J. L. Capener, J. W. Bröckel, L. Vu, C. C. King, V. L. Ruiz-Perez, X. Ge, G. Veglia, F. W. Herberg, S. S. Taylor, B. R. Myers, A PKA inhibitor motif within SMOOTHENED controls Hedgehog signal transduction. *Nat Struct Mol Biol* **29**, 990–999 (2022).

27. S. Mukhopadhyay, X. Wen, N. Ratti, A. Loktev, L. Rangell, S. J. Scales, P. K. Jackson, The Ciliary G-Protein-Coupled Receptor Gpr161 Negatively Regulates the Sonic Hedgehog Pathway via cAMP Signaling. *Cell* **152**, 210–223 (2013).

28. K. Pal, S. Hwang, B. Somatilaka, H. Badgandi, P. K. Jackson, K. DeFea, S. Mukhopadhyay, Smoothened determines β-arrestin–mediated removal of the G protein–coupled receptor Gpr161 from the primary cilium. *Journal of Cell Biology* **212**, 861–875 (2016).

29. P. M. Tschaikner, D. Regele, R. Röck, W. Salvenmoser, D. Meyer, M. Bouvier, S. Geley, E. Stefan, P. Aanstad, Feedback control of the Gpr161-Gαs-PKA axis contributes to basal Hedgehog repression in zebrafish. *Development* **148**, dev192443 (2021).

30. G. V. Pusapati, J. H. Kong, B. B. Patel, M. Gouti, A. Sagner, R. Sircar, G. Luchetti, P. W. Ingham, J. Briscoe, R. Rohatgi, G protein–coupled receptors control the sensitivity of cells to the morphogen Sonic Hedgehog. *Sci. Signal.* **11**, eaao5749 (2018).

31. J. H. Kong, C. Siebold, R. Rohatgi, Biochemical mechanisms of vertebrate hedgehog signaling. *Development* **146**, dev166892 (2019).

32. C. E. Wang, S. K. Ogden, G Protein-Coupled Receptor Signal Intersection at the Primary Cilium. *BioEssays* **47**, e70015 (2025).

33. S. Yoshida, K. Yoshida, Regulatory mechanisms governing GLI proteins in hedgehog signaling. *Anat Sci Int* **100**, 143–154 (2025).

34. V. A. Bachmann, J. E. Mayrhofer, R. Ilouz, P. Tschaikner, P. Raffeiner, R. Röck, M. Courcelles, F. Apelt, T.-W. Lu, G. S. Baillie, P. Thibault, P. Aanstad, U. Stelzl, S. S. Taylor, E. Stefan, Gpr161 anchoring of PKA consolidates GPCR and cAMP signaling. *Proc. Natl. Acad. Sci. U.S.A.* **113**, 7786–7791 (2016).

35. N. Hoppe, S. Harrison, S.-H. Hwang, Z. Chen, M. Karelina, I. Deshpande, C.-M. Suomivuori, V. R. Palicharla, S. P. Berry, P. Tschaikner, D. Regele, D. F. Covey, E. Stefan, D. S. Marks, J. F. Reiter, R. O. Dror, A. S. Evers, S. Mukhopadhyay, A. Manglik, GPR161 structure uncovers the redundant role of sterol-regulated ciliary cAMP signaling in the Hedgehog pathway. *Nat Struct Mol Biol* **31**, 667–677 (2024).

36. G. S. Anand, M. Hotchko, S. H. J. Brown, L. F. Ten Eyck, E. A. Komives, S. S. Taylor, R-subunit Isoform Specificity in Protein Kinase A: Distinct Features of Protein Interfaces in PKA Types I and II by Amide H/2H Exchange Mass Spectrometry. *Journal of Molecular Biology* **374**, 487–499 (2007).

37. P. Zhang, E. V. Smith-Nguyen, M. M. Keshwani, M. S. Deal, A. P. Kornev, S. S. Taylor, Structure and Allostery of the PKA RIIβ Tetrameric Holoenzyme. *Science* **335**, 712–716 (2012).

38. T.-W. Lu, J. Wu, P. C. Aoto, J.-H. Weng, L. G. Ahuja, N. Sun, C. Y. Cheng, P. Zhang, S. S. Taylor, Two PKA RIα holoenzyme states define ATP as an isoform-specific orthosteric inhibitor that competes with the allosteric activator, cAMP. *Proc. Natl. Acad. Sci. U.S.A.* **116**, 16347–16356 (2019).
