# Supplementary material for: Smoothened and ciliary GPCRs regulate ciliary protein kinase A activity involved in Hedgehog signal transduction
Source: PLoS Biol. 2026 Jun 10;24(6):e3003841. doi: 10.1371/journal.pbio.3003841 (PMC13289941; doi:10.1371/journal.pbio.3003841)
Supplement: Attachment — Submitted filename: Nguyen et al Response to Reviewers 2.docx [file pbio.3003841.s018.docx]

April 13, 2026

Dear Referees,

Thank you for your additional helpful comments. We were heartened to read that we had provided “the needed documentation of the antibodies” and have done “a better job of putting the work into context” (Reviewer 1), that “I do consider the work an important contribution to the mechanistic question of how PKA is activated in primary cilia and how this mediates SHH signaling (Reviewer 2), and that “the additional experiments performed by the authors largely addressed this concern and added more mechanistic insight.” (Reviewer 3).

Your full comments are appended in full, below, together with a description of how we have endeavored to address the critiques in blue font.

Reviewers' comments

Reviewer 1:

In this revision, Nguyen and colleagues revised their previous work to describe a ciliary PKA reporter. My previous concerns focused on needed documentation of the antibodies and their characterization, and the failure of the discussion to put the work into the context of existing data. The discussion now does a better job of putting the work into context. The sources of the antibodies have been provided, but I see little in the way of discussion of what validation they were subjected to.

Below, we discuss the provenance of these antibodies, how they’ve been characterized in previous papers and how we have performed additional validation. Please let us know if we should include some of this analysis in the paper.

Anti-VASP (phospho S157) antibody [5C6] ab58555

Vasodilator-stimulated phosphoprotein (VASP) was originally characterized as a substrate of both cAMP-dependent kinase PKA and cGMP-dependent kinase PKG (also known as cAPK and cGPK, respectively) (Butt *et al.*, 1994). PKG phosphorylates VASP at Ser239, and PKA phosphorylates VASP at Ser157 (Smolenski *et al.*, 1998).

Anti-VASP (phospho S157) antibody [5C6] ab58555 (pVASP) antibody was previously characterized by O’Banion et al., 2018 and O’Banion et al., 2019 to detect subcellular PKA activity. They have shown previously that exposure of MV^D7^ cells to the adenylyl cyclase (AC) activator, forskolin (FSK), resulted in the phosphorylation of a VASP-based mitochondrial-targeted PKA Reporter, plasma membrane-targeted PKA reporter (O’Banion *et al.*, 2018) and nuclear-targeted PKA reporter (O’Banion *et al.*, 2019).

Similarly, in our paper, we show that that FSK increases pVASP signal at the primary cilium in our Cilia-PKA reporter cells (**Figure 1B, 1C, 1E, S1A-B, S1C-E**), and that simultaneous treatment of FSK and H89 abolishes this signal (**Figure 1D-E, S1C**). From this result, we conclude that the antibody recognizes an antigen generated in response to FSK and inhibited in response to H89.

To perform further validation of this antibody, we have immunostained the parental cell line, wild-type NIH-3T3 Flp-In cells, with the pVASP antibody following the protocol described in Methods and Materials (**Reviewer Figure 1**). For the purposes of detecting background, we increased the laser power and gain in the pVASP channel (**Reviewer Figure 1A, 1B**). As performed in the paper, we also treated these cells with SAG to test whether SAG would affect pVASP staining (**Reviewer Figure 1B**). We did not detect any pVASP signal in the cilium, either in the vehicle treated condition, nor in the SAG treated condition (**Reviewer Figure 1A-C**). Low levels of background were detected in the nucleus.

**Reviewer Figure 1. Ciliary pVASP signal is undetectable in the absence of the ciliary PKA reporter.**

**(A-B)** Immunofluorescence imaging of the parental NIH-3T3 Flp-In cells which were treated with either vehicle (**A**), or SAG (100nM for 24 hours) (**B**). Images depict cells immunostained for pVASP (pVASP^S157^, yellow), cilia (acetylated tubulin, TUB^AC^, cyan) and nuclei (Hoechst, grey). Scale bar is 5µM. Inset scale bar is 1µM. (**C**) Quantification of ciliary pVASP intensity. Significance was determined via unpaired t-test. P values are indicated as follows: *p < 0.04, ** p < 0.003.

pS594/pT597/pS599-SMO (phospho-SMO Receptor Antibody) 7TM0239A-IC

Arveseth et al. 2021 utilized quantitative mass spectrometry to identify 7 sites on the C-terminal tail of SMO (S560, S594/T597/S599, and S642/T644/T648), that exhibited phosphorylation dependent on SMO and GRK2/3 activity (Arveseth *et al.*, 2021).

In the subsequent paper from the same group, Walker et al., 2024 raised a phospho-specific antibody by immunizing rabbits with a phosphorylated peptide spanning a cluster of these GRK2/3 phosphorylation sites that they had previously identified in the SMO intracellular membrane (S594, T597, and S599). From these rabbits, they purified an antibody that they called pS594/pT597/pS599-SMO (phospho-SMO Receptor Antibody) 7TM0239A-IC (Walker *et al.*, 2024). As reported in Figure 2 of Walker et al., 2024, the authors treated cells with SAG and found that this antibody recognizes an antigen that accumulates at the primary cilium, consistent with this antigen being a form of SMO. Compound 101 (CMPD101) is a selective inhibitor of GRK2/3 kinases (Ikeda, Kaneko and Fujiwara, 2006; Thal *et al.*, 2011). Walker et al., 2024 treated cells simultaneously with SAG and Compound 101 and observed that the antigen was no longer recognized by the antibody, and concluded that this antibody specifically recognizes a GRK2/3-phosphorylated pool of SMO, referred to as phosphorylated SMO or pSMO (Walker *et al.*, 2024).

We repeated the aforementioned experiment and include the data as part of Figure 4. We also found that treatment of cells with SAG and CMPD101 prevented enrichment of pSMO at the primary cilium. Therefore, we concur that the pSMO antibody recognizes a GRK2/3-phosphorylated form of SMO.

To address whether S594/T597/S599 could also be phosphorylated by casein kinase 1 (CK1), Walker et al., 2024 treated their cells simultaneously with SAG and D4476, an inhibitor of all CK1 isoforms (Rena *et al.*, 2004; Bain *et al.*, 2007) and found that this antibody still recognized an antigen whose localization to the cilium was stimulated by SAG (Walker et al., 2024 Figure 2B). These data suggest that the pSMO antibody recognizes a pool of SMO phosphorylated by GRK2/3 but not phosphorylated by another kinase implicated in Hedgehogs signal transduction, CK1.

The big problem with the current submission is the new figure 7. The investigators knocked out GPR161, but the data were inconsistent with expectations: the loss of GPR161 did not alter (reduce) ciliary PKA activity as expected, nor did over-expression increase ciliary PKA activity. Further compounding the confusion, expression of an L465P mutant form of GPR161 in the KO background did have the expected reduction in ciliary pVASP signal. This would suggest a dominant negative activity, but the L465P construct failed to enrich PKA-R or PKA-C seen with wild type, making the idea of a dominant activity hard to justify.

We concur with the reviewer that the data are not consistent with prior expectations. However, as these experiments were well controlled and performed multiple times, we propose that the results represent a chance to contribute to understanding of the function of GPR161. We concur that the results are consistent with GPR161 L465P acting via a dominant negative mechanism, but via a mechanism independent of the role of GPR161 in affecting the localization of PKA-R or PKA-C to cilium. We will continue to investigate what this dominant negative mechanism is as a worthy subject for follow-up investigation.

The materials and methods are vague about how the genomic DNA was analyzed to demonstrate a strong allele and the western is underwhelming in quality, so it is possible that this is not a null allele.

The Materials and Methods section now includes the following description:

“We used clustered regularly interspaced short palindromic repeats (CRISPR)-mediated editing to generate loss-of-function mutations in *Smo* and *Gpr161* using two different guide RNAs per gene in the cilia PKA reporter cells. Synthetic guide RNAs for mouse *Smo* (5’-CCCACGCACGGGGCGGCCAG-3’, 5’-UCCCGCUCAAGGCCGCCCCC-3’) and *Gpr161* (5’ AUGCGGUGAGCAGAGCAUGC-3’, 5’- GAGGGAGGAGUUGAGGCUCA-3’) were ordered from Synthego, complexed with TrueCut Cas9 Protein v2 (Invitrogen, A36496), and nucleofected with the Neon Transfection System (Invitrogen). Cells were clonally selected and screened via PCR for genomic deletions with primers for the genomic regions of interest for mouse *Smo* (forward primer: 5’-AGGGTTCCCAGGGTTGAAGA-3’, reverse primer: 5’-CACACGTTGTAGCGCAAAGG-3’) and mouse *Gpr161* (forward primer 5’- GGAGGTTCCAAACACATTGGC-3’, reverse primer: 5’-CGATGAACTCAGAGACGGCA-3’).”

We now include the DNA sequencing data of the *Smo*^-/-^ and *Gpr161*^-/-^ clones used in this paper as the new **Figures S2** and **S6**, respectively.

More specifically, Sanger sequencing revealed that the *Smo*^-/-^ cell line that we use has 3 alleles (as expected as NIH-3T3 cells are hypertriploid, <https://www.atcc.org/products/ccl-92>). One allele is a 10bp and 1 bp deletion allele resulting in an early frame shift and stop codon. The second allele is an 85bp deletion allele resulting in an early frame shift and stop codon. The third allele is a 139bp deletion that deletes the start codon. No wild-type sequences are detected in DNA from this clone. Thus, we conclude that this cell line is likely to be null for *Smo*.

Sanger sequencing revealed that the *Gpr161*^-/-^ clone is a 38bp deletion resulting in an early frame shift and stop codon. (We previously included a second clone of Gpr161, clone 2, which Sanger sequencing revealed to possess alleles that contain 38bp deletion and a 37 bp deletion. We have removed this clone from the manuscript as it did not add new data.) No wild-type sequences are detected in DNA. Thus, we conclude that this cell line is likely to be null for *Gpr161*.

Did this mutation show the other expected phenotypes for a GPR161 KO? Did you see increased Gli2 at the ciliary tip of unstimulated cells? Does Smo accumulate (I
expect it would, based on review articles, but I was not able to find the original data to support this point)? As this figure now stands, the data seriously undermines the value of the reporter and the importance of the rest of the manuscript. Perhaps the key experiments need to be repeated in cells where a GPR161 effect is observed.

As suggested by the reviewer, we examined GLI2 localization at the cilia tip and SMO accumulation in the cilia of GPR161 KO cells. We did not observe a difference in ciliary SMO intensities in WT vs *Gpr161*^-/-^ cells (**Reviewer Figure 2 A-C**). Independence of SMO accumulation in cilia from GPR161 function is consistent with prior work indicating that GPR161 functions epistatically to SMO (Mukhopadhyay *et al.*, 2013). Should the reviewer recommend it, we would be happy to include these data in the manuscript.

**Reviewer Figure 2. SMO does not accumulate at the cilia of *Gpr161*^-/-^ cells**

**(A-B)** Immunofluorescence imaging of the Cilia PKA Reporter cells (**A**), as well as *Gpr161^-/-^* Cilia PKA Reporter Cells (**B**); magnifications of 4 representative cilia per condition are highlighted. Images depict cells immunostained for SMO (SMO, yellow), cilia PKA reporter (GFP, cyan) and nuclei (Hoechst, grey) Scale bar is 5µM, 2.5µM for insets.

(**C**) Quantification of ciliary SMO intensity. Representative images used for quantification are in **Reviewer Figure 2 A-B**. Each biological replicate is color coded. Significance was determined via unpaired t-test. P values are indicated as follows: *p < 0.04, ** p < 0.003. Data are represented are means of replicates ± SD.

Previous articles (Hwang et al., 2021 Figure 4D, 4E and Hoppe et al., 2021 Figure 5A, 5B) reported that GLI2 accumulated at the ciliary tip in GPR161 KO cells. These papers performed their quantitation as cilia with and without GLI2 (Hwang *et al.*, 2021; Hoppe *et al.*, 2024). As requested, we stained for GLI2 in our WT and KO cells (**Reviewer Figure 3**). We include images of GLI2, γ−Tubulin (basal body) and GFP (cilia) stains in WT, WT+SAG, and *Gpr161^-/-^* cells. Magnifications of 8 representative cilia per condition are highlighted (**Reviewer Figure 3A-C**).

As expected, activating the Hedgehog signal transduction pathway with SAG increased ciliary GLI2 intensities, indicating that we were able to detect pathway-dependent changes in GLI2 ciliary localization sensitively (**Reviewer Figure 3B**).

We found that ciliary GLI2 localization was not increased in GPR161 KO cells, and that GLI2 tip localization was not dichotomous. Specifically, we found that in WT cells, GLI2 can be detected both at the ciliary tip (**Reviewer Figure 3A insets 1, 2, 5, 6, 7, 8**), and along the length of the cilium (**Reviewer Figure 3A insets 5, 7, 8**). Similarly, we found that in *Gpr161^-/-^* cells, GLI2 can also be detected both at the ciliary tip (**Reviewer Figure 3C all insets**), and along the length of the cilium (**Reviewer Figure 3B insets 2, 4, 5**).

These data indicate that the amount of GLI2 ciliary localization is a continuous variable. We then sought to analyze this continuous variable as a continuous variable, as supported by statistical best practices (Altman and Royston, 2006). Because dichotomization discards data, it can increase the risk of a positive result being a false positive (Austin and Brunner, 2004). We calculated total ciliary GLI2 intensity (**Reviewer Figure 3D**), and found that ciliary GLI2 localization was not increased in GPR161 KO cells.

**Reviewer Figure 3. GLI2 localization to the primary cilia of WT and *Gpr161^-/-^* cells is equivalent.**

**(A-C)** Immunofluorescence imaging of the Cilia PKA Reporter cells (**A**), Cilia PKA Reporter cells treated with 100nM SAG for 24hrs (**B**), and *Gpr161^-/-^* Cilia PKA Reporter Cells (**C**); magnifications of 8 representative cilia per condition are highlighted. Images depict cells immunostained for Gli2 (GLI2, yellow), centrosomes (yTub, Magenta), cilia PKA reporter (GFP, cyan) and nuclei (Hoechst, grey). White arrows indicate location of the ciliary tip. Scale bar is 5µM, 2.5µM for insets.
(**D**) Quantification of ciliary GLI2 intensity. Representative images used for quantification are in **Reviewer Figure 3 A-C**. Each biological replicate is color coded. Significance was determined via unpaired t-test. P values are indicated as follows: *p < 0.04, ** p < 0.003. Data are represented are means of replicates ± SD.

We suggest that these data provide new insights that, although discrepant with prior report, may advance understanding of the role of GPR161 in ciliary Hedgehog signaling transduction. In addition, the rest of the manuscript that the reviewer alludes to tests whether:

1) SMO inhibits ciliary PKA
2) Gα_i/o_ inhibits ciliary PKA
3) SMO regulates ciliary PKA through Gα_i/o_ or through direct inhibition, and
4) whether GPR161 regulates ciliary PKA activity.

Does the reviewer advise us to remove the GPR161 data from this work? We certainly have no desire to undermine the importance of the rest of the manuscript.

Reviewer 2:

I very much appreciate the work that the authors put in the revised version of this manuscript. In the light of the newly presented data, expanding the discussion will certainly be valued by the readers. While I am also thankful for the thorough assessment of this work by the other reviewers, I do not agree with reviewer 3 that this work is "only a modest advance in our mechanistic understanding of SHH pathway regulation". Many different models of how PKA activity in primary cilia may be regulated in SHH signaling are being discussed. The presented data make a strong case that SMO-based inhibition of PKA activity can be independent of G protein-coupling, which is consistent with previous work by other groups. At the same time, the work shows that ciliary PKA activity can also be regulated by "conventional" GPCR signaling via G protein-coupling (see Figure 5), likely due to changes in ciliary cAMP, which is also consistent with previous work by several groups. Therefore, I do consider the work an important contribution to the mechanistic question of how PKA is activated in primary cilia and how this mediates SHH signaling.

Unfortunately, the authors fail to provide seemingly trivial but essential information. The authors now present additional experiments in a newly generated GPR161-deficient cell line, which is very elegant. However, the reader does not get sufficient information on this cell line. Because this cell line is so central to their conclusion and because it brings up a new, very puzzling result (see comments below), I must insist that the authors provide data that the cell line is indeed deficient in GPR161.

Thank you for your comments. We apologize that we did not provide adequate data that the newly generated GPR161-deficient cell line is indeed deficient in GPR161. As also addressed above to Reviewer 1, we have now included Sanger sequencing data for the *Gpr161*^-/-^ cell line. These data are provided as the new Supplemental Figure 6. In brief, Sanger sequencing revealed that the *Gpr161*^-/-^ clone is a 38bp deletion resulting in an early frame shift and stop codon. No wild-type sequences were detected in DNA from these cells. Thus, we conclude that this cell line is likely to be null for *Gpr161*.

Additional points:

1. It is unclear why the authors do not include information on the antibodies they used for the new experiments, as this was one of the few requests raised by reviewer 1. Please include this critical information, also in future publications. It is essential information and you know it.

We apologize. We had indeed forgotten to update our Materials and Methods section. We now include under “Immunofluorescence staining,” the catalog numbers for all antibodies used in this work, including two new ones:

Anti-PKA-C (BD Biosciences 610981)

Anti-PKA-R (BD Biosciences 610165)

2. Figure 7: The changes in the localization of the PKA subunits are striking. However, the result on the PKA reporter in the L465P mutant is very puzzling. The authors provide a potential interpretation; however, a much simpler interpretation is that the putative GPR161-/- is not a full knockout, and hence the L465P might have a dominant-negative effect on PKA activation on wildtype GPR161. The authors must provide unambiguous evidence that the mutant is indeed lacking GPR161.

We have now provided Sanger sequencing data for the *Gpr161*^-/-^ cell line. These data are now included in the new Supplemental 7. In brief, Sanger sequencing revealed that the *Gpr161*^-/-^ clone is a 38bp deletion resulting in an early frame shift and stop codon. No wild-type sequences were detected in DNA from these cells. Thus, we conclude that this cell line is likely to be null for *Gpr161*.

3. The new Figure 7 caption in its current form is not as informative as other captions. Understanding the figure requires reading the main text. Maybe this can be changed, i.e. by hinting at the L465P mutation.

Thank you. We have updated the figure caption as you have suggested.

4. The extended discussion will be very valuable to the readers of this article. The authors' insight into the AKAP function of GPR161 and how it regulates PKA activity by directing PKA holoenzymes to their sites of action is a very important result, but they should be acknowledge that they were not the first to propose that GPR161 functions mainly by localizing the PKA holoenzymes to primary cilia. They ought to cite either Bachmann et al., 2016 (PMID: 27357676) or Tschaikner et al., 2020 (PMID: 31947770) who proposed this, and May et al., 2021 (PMID: 33856408) who provided evidence that the ciliary localization of PKA is indeed regulated in response to SHH signaling.

We appreciate the correction. It was not our intent to not acknowledge these important works. We have now expanded the Discussion to include these additional citations. Excerpts of the revised Discussion are:

“Inspired by recent discoveries that SMO inhibits PKA and that HH signaling causes PKA to leave cilia along with GPR161 (Arveseth *et al.*, 2021; May *et al.*, 2021; Happ *et al.*, 2022), we hypothesized that SMO activates HH signaling by specifically inhibiting PKA at cilia.”

“Previously, Bachmann et al. demonstrated that GPR161 recruits PKA-RIa to primary cilia, Tschaikner et al demonstrated that GPR161 can biochemically interact with PKA-R and PKA-C, and May et al. demonstrated that HH signaling can stimulate the departure of PKA-RIa from primary cilia (Bachmann *et al.*, 2016; May *et al.*, 2021; Tschaikner *et al.*, 2021). Our results are consistent with GPR161 recruiting both PKA-R and PKA-C to the cilium.”

Reviewer 3:

My main concern with this manuscript during the initial review was that despite the utility of the PKA reporter, the paper represented an incremental advance from other recent papers on the mechanisms of SMO activation/PKA inhibition in the primary cilium. I feel that the additional experiments performed by the authors largely addressed this concern and added more mechanistic insight. A few points do remain to be addressed prior to publication, however:

Reviewer 2 made a point about the ability of the reporter to detect base-line levels of PKA activity absent FSK. While the authors address this point, there remain inconsistencies with respect to what baseline levels look like throughout the manuscript. For example, there is still an apparent discrepancy between what is shown in Fig 1- which is no detection of pVASP in the cilium, and Fig.3 which shows baseline localization that disappears upon stimulation with SAG. Lack of detection in cilia is also what is presented in Fig S1. Figure 7 discusses baseline reporter activity in WT and GPR161-/- cells, however here we see intense localization of the reporter in both of these cases, which seems to be inconsistent with Fig. 1. Were these cells treated with FSK? If so they should be labeled as such.

We are sorry for the confusion. The cells in Figure 7 were not treated with FSK.

The laser power and gain used during microscopy for conditions Figure 1 and Figure 7 are different. That is, the pVASP stain in the cilia PKA reporter cells when treated with FSK (as in Figure 1) is elevated such that, to avoid saturating the channel and failing to capture the full dynamic range, a lower laser power is used when cells are not treated with FSK (as in Figure 7).

These same considerations are also true of Figure 5 and Supplemental Figure 4 (formerly labeled Supplemental Figure 5). Images used as data for Figure 5 were generated with a higher laser power than the images used for Supplemental Figure 4. This consideration is also why there is more background pVASP signal in Figure 7 and Figure 5 than in Figure 1 and Supplemental Figure 4.

We have now edited the Figure captions of Figure 5 and Figure 7 to indicate that the laser power for the pVASP channel was higher than used in other figures. We have also included a discussion of this issue in the revised Materials and Methods. Excerpt of the revised Materials and Methods section here:

“We imaged fixed cells on a DeltaVision-OMX-SR (GE Healthcare) equipped with a Plan ApoN 60X/1.42 Oil objective and three PCO.edge 5.5 15bit sCMOS Cameras (liquid cooled). Four-channel fluorescence imaging was captured with a Toptica 4 line laser launch light source, laser excitation wavelengths 405nm/488nm/568nm/642nm, and emission filters 435/31m, 528/48m, 609/37m, and 683/40m. To detect basal levels of pVASP without FSK treatment in Figure 7 and Figure 5D, we used a higher laser power (20% in 568nm excitation) than in the other figures (10% in 568nm excitation).”

In Fig 6 panel B, I am assuming the SMO-WRR, SMO-GRL, and SMO-WRR-GRL were all treated with SAG. The labeling of the figures themselves should reflect this, otherwise it appears that these mutants behave like SMO-M2 and constitutively inhibit PKA activity.

Yes, those conditions (SMO-WRR, SMO-GRL and SMO-WRR-GRL) in Figure 6B were indeed treated with SAG. We have now labeled them appropriately.

Minor:
In Figure 6 the various SMO mutants are discussed in a different order in the text than how they appear in panels 6B, C, and D. Since this is a complex figure, keeping the order consistent will help the reader.

Thank you for the helpful suggestion. We have now re-arranged both Figure 6 and Supplement Figure 5 (formerly labeled Supplemental Figure 6), so that the order the data appears matches the order discussed in the text.

There is no Fig S2- goes from S1 to S3

Thank you. We have re-labeled our figures to fix this error.

Thank you again, reviewers, for your time, attention and care given to helping us improve this manuscript. We are in debt to you!

Sincerely,

Jeremy Reiter, MD, PhD

Professor

Department of Biochemistry and Biophysics

University of California, San Francisco

References for reviewers

Altman, D.G. and Royston, P. (2006) “The cost of dichotomising continuous variables,” *BMJ*, 332(7549), p. 1080. Available at: https://doi.org/10.1136/bmj.332.7549.1080.

Arveseth, C.D. *et al.* (2021) “Smoothened transduces Hedgehog signals via activity-dependent sequestration of PKA catalytic subunits,” *PLOS Biology*. Edited by K. Basler, 19(4), p. e3001191. Available at: https://doi.org/10.1371/journal.pbio.3001191.

Austin, P.C. and Brunner, L.J. (2004) “Inflation of the type I error rate when a continuous confounding variable is categorized in logistic regression analyses,” *Statistics in Medicine*, 23(7), pp. 1159–1178. Available at: https://doi.org/10.1002/sim.1687.

Bachmann, V.A. *et al.* (2016) “Gpr161 anchoring of PKA consolidates GPCR and cAMP signaling,” *Proceedings of the National Academy of Sciences*, 113(28), pp. 7786–7791. Available at: https://doi.org/10.1073/pnas.1608061113.

Bain, J. *et al.* (2007) “The selectivity of protein kinase inhibitors: a further update,” *The Biochemical Journal*, 408(3), pp. 297–315. Available at: https://doi.org/10.1042/BJ20070797.

Butt, E. *et al.* (1994) “cAMP- and cGMP-dependent protein kinase phosphorylation sites of the focal adhesion vasodilator-stimulated phosphoprotein (VASP) in vitro and in intact human platelets,” *The Journal of Biological Chemistry*, 269(20), pp. 14509–14517.

Happ, J.T. *et al.* (2022) “A PKA inhibitor motif within SMOOTHENED controls Hedgehog signal transduction,” *Nature Structural & Molecular Biology*, 29(10), pp. 990–999. Available at: https://doi.org/10.1038/s41594-022-00838-z.

Hoppe, N. *et al.* (2024) “GPR161 structure uncovers the redundant role of sterol-regulated ciliary cAMP signaling in the Hedgehog pathway,” *Nature Structural & Molecular Biology*, 31(4), pp. 667–677. Available at: https://doi.org/10.1038/s41594-024-01223-8.

Hwang, S.-H. *et al.* (2021) “Ciliary and extraciliary Gpr161 pools repress hedgehog signaling in a tissue-specific manner,” *eLife*, 10, p. e67121. Available at: https://doi.org/10.7554/eLife.67121.

Ikeda, S., Kaneko, M. and Fujiwara, S. (2006) “Cardiotonic agent comprising grk inhibitor.”

May, E.A. *et al.* (2021) “Time-resolved proteomics profiling of the ciliary Hedgehog response,” *Journal of Cell Biology*, 220(5), p. e202007207. Available at: https://doi.org/10.1083/jcb.202007207.

Mukhopadhyay, S. *et al.* (2013) “The Ciliary G-Protein-Coupled Receptor Gpr161 Negatively Regulates the Sonic Hedgehog Pathway via cAMP Signaling,” *Cell*, 152(1–2), pp. 210–223. Available at: https://doi.org/10.1016/j.cell.2012.12.026.

O’Banion, C.P. *et al.* (2018) “Design and Profiling of a Subcellular Targeted Optogenetic cAMP-Dependent Protein Kinase,” *Cell Chemical Biology*, 25(1), pp. 100-109.e8. Available at: https://doi.org/10.1016/j.chembiol.2017.09.011.

O’Banion, C.P. *et al.* (2019) “Compartmentalized cAMP Generation by Engineered Photoactivated Adenylyl Cyclases,” *Cell Chemical Biology*, 26(10), pp. 1393-1406.e7. Available at: https://doi.org/10.1016/j.chembiol.2019.07.004.

Rena, G. *et al.* (2004) “D4476, a cell-permeant inhibitor of CK1, suppresses the site-specific phosphorylation and nuclear exclusion of FOXO1a,” *EMBO reports*, 5(1), pp. 60–65. Available at: https://doi.org/10.1038/sj.embor.7400048.

Smolenski, A. *et al.* (1998) “Analysis and Regulation of Vasodilator-stimulated Phosphoprotein Serine 239 Phosphorylation in Vitro and in Intact Cells Using a Phosphospecific Monoclonal Antibody,” *Journal of Biological Chemistry*, 273(32), pp. 20029–20035. Available at: https://doi.org/10.1074/jbc.273.32.20029.

Thal, D.M. *et al.* (2011) “Molecular Mechanism of Selectivity among G Protein-Coupled Receptor Kinase 2 Inhibitors,” *Molecular Pharmacology*, 80(2), pp. 294–303. Available at: https://doi.org/10.1124/mol.111.071522.

Tschaikner, P.M. *et al.* (2021) “Feedback control of the Gpr161-Gαs-PKA axis contributes to basal Hedgehog repression in zebrafish,” *Development*, 148(4), p. dev192443. Available at: https://doi.org/10.1242/dev.192443.

Walker, M.F. *et al.* (2024) “GRK2 kinases in the primary cilium initiate SMOOTHENED-PKA signaling in the Hedgehog cascade,” *PLOS Biology*. Edited by K. Basler, 22(8), p. e3002685. Available at: https://doi.org/10.1371/journal.pbio.3002685.
