# Supplementary material for: Smoothened and ciliary GPCRs regulate ciliary protein kinase A activity involved in Hedgehog signal transduction
Source: PLoS Biol. 2026 Jun 10;24(6):e3003841. doi: 10.1371/journal.pbio.3003841 (PMC13289941; doi:10.1371/journal.pbio.3003841)
Supplement: Peer review history

# Editor report: Decision Letter 0

**DOI:** 10.1371/journal.pbio.3003841.r001

**Authors:** Ines Alvarez-Garcia

21 Apr 2025

Dear Dr Reiter,

Thank you for submitting your manuscript entitled "Smoothened inhibition of PKA at cilia transduces Hedgehog signals" for consideration as a Research Article by PLOS Biology.

Your manuscript has now been evaluated by the PLOS Biology editorial staff as well as by an academic editor with relevant expertise and I am writing to let you know that we would like to send your submission out for external peer review.

However, before we can send your manuscript to reviewers, we need you to complete your submission by providing the metadata that is required for full assessment. To this end, please login to Editorial Manager where you will find the paper in the 'Submissions Needing Revisions' folder on your homepage. Please click 'Revise Submission' from the Action Links and complete all additional questions in the submission questionnaire.

Once your full submission is complete, your paper will undergo a series of checks in preparation for peer review. After your manuscript has passed the checks it will be sent out for review. To provide the metadata for your submission, please Login to Editorial Manager (https://www.editorialmanager.com/pbiology) within two working days, i.e. by Apr 23 2025 11:59PM.

If your manuscript has been previously peer-reviewed at another journal, PLOS Biology is willing to work with those reviews in order to avoid re-starting the process. Submission of the previous reviews is entirely optional and our ability to use them effectively will depend on the willingness of the previous journal to confirm the content of the reports and share the reviewer identities. Please note that we reserve the right to invite additional reviewers if we consider that additional/independent reviewers are needed, although we aim to avoid this as far as possible. In our experience, working with previous reviews does save time.

If you would like us to consider previous reviewer reports, please edit your cover letter to let us know and include the name of the journal where the work was previously considered and the manuscript ID it was given. In addition, please upload a response to the reviews as a 'Prior Peer Review' file type, which should include the reports in full and a point-by-point reply detailing how you have or plan to address the reviewers' concerns.

During the process of completing your manuscript submission, you will be invited to opt-in to posting your pre-review manuscript as a bioRxiv preprint. Visit http://journals.plos.org/plosbiology/s/preprints for full details. If you consent to posting your current manuscript as a preprint, please upload a single Preprint PDF.

Feel free to email us at plosbiology@plos.org if you have any queries relating to your submission.

Kind regards,

Ines —

Ines Alvarez-Garcia, PhD

Senior Editor

PLOS Biology

ialvarez-garcia@plos.org

# Peer review: Decision Letter 1

**DOI:** 10.1371/journal.pbio.3003841.r002

**Authors:** Ines Alvarez-Garcia

6 Jun 2025

Dear Dr Reiter,

Thank you for your patience while your manuscript entitled "Smoothened inhibition of PKA at cilia transduces Hedgehog signals" was peer-reviewed at PLOS Biology. Your manuscript has been evaluated by the PLOS Biology editors, an Academic Editor with relevant expertise, and by three independent reviewers.

The reviews are attached below. As you will see, the reviewers find the conclusions interesting but mostly confirmatory of previous findings. In addition, they raise several issues that would need to be addressed before we can consider the manuscript for publication. Reviewer 1 thinks that the methods and discussion need to be improved, and asks for several clarifications. Reviewer 2 sees the value of the tool for the community, but also mentions that it has limitations as it cannot follow the PKA activity in living cells and can only assess one point in time in the primary cilium. In addition, this reviewer thinks that some of the findings seem contradictory and suggests an experiment inducing short-term SMO activation when increased levels of cAMP are present in cilia to see if the results are similar in more physiological conditions. The reviewer also suggests additional experiments to strengthen the conclusions. Reviewer 3 thinks that the biosensor is an important tool that has enabled confirmation of prior findings with spatial resolution, but that substantial additional findings that provide new mechanistic insights should be added to consider the manuscript for publication.

Based on the reviewers’ comments and following discussion with the Academic Editor, it is clear that a substantial amount of work would be required to meet the criteria for publication in PLOS Biology. However, given our and the reviewers interest in your study, we would be open to inviting a comprehensive revision of the study that thoroughly addresses all the reviewers' comments and significantly adds novel mechanistic insights. Given the extent of revision that would be needed, we cannot make a decision about publication until we have seen the revised manuscript and your response to the reviewers' comments. Your revised manuscript would need to be seen by the reviewers again, but please note that we would not engage them unless their main concerns have been addressed.

In addition to these revisions, you will need to complete some formatting changes, which you will receive in a follow up email. A member of our team will be in touch with a set of requests shortly.

We appreciate that these requests represent a great deal of extra work, and we are willing to relax our standard revision time to allow you 6 months to revise your study. Please email us (plosbiology@plos.org) if you have any questions or concerns, or envision needing a short extension.

At this stage, your manuscript remains formally under active consideration at our journal; please notify us by email if you do not intend to submit a revision so that we may withdraw it.

**IMPORTANT - SUBMITTING YOUR REVISION**

Your revisions should address the specific points made by each reviewer. Please submit the following files along with your revised manuscript:

1. A 'Response to Reviewers' file - this should detail your responses to the editorial requests, present a point-by-point response to all of the reviewers' comments, and indicate the changes made to the manuscript.

*NOTE: In your point by point response to the reviewers, please provide the full context of each review. Do not selectively quote paragraphs or sentences to reply to. The entire set of reviewer comments should be present in full and each specific point should be responded to individually, point by point.

You should also cite any additional relevant literature that has been published since the original submission and mention any additional citations in your response.

2. In addition to a clean copy of the manuscript, please also upload a 'track-changes' version of your manuscript that specifies the edits made. This should be uploaded as a "Revised Article with Changes Highlighted " file type.

3. Resubmission Checklist

When you are ready to resubmit your revised manuscript, please refer to this resubmission checklist: https://plos.io/Biology_Checklist

To submit a revised version of your manuscript, please go to https://www.editorialmanager.com/pbiology/ and log in as an Author. Click the link labelled 'Submissions Needing Revision' where you will find your submission record.

Please make sure to read the following important policies and guidelines while preparing your revision and fulfil the editorial requests:

a) *PLOS Data Policy*

Please note that as a condition of publication PLOS' data policy (http://journals.plos.org/plosbiology/s/data-availability) requires that you make available all data used to draw the conclusions arrived at in your manuscript. If you have not already done so, you must include any data used in your manuscript either in appropriate repositories, within the body of the manuscript, or as supporting information (N.B. this includes any numerical values that were used to generate graphs, histograms etc.). Please also indicate in each figure legend where the data can be found. For an example see here: http://www.plosbiology.org/article/info%3Adoi%2F10.1371%2Fjournal.pbio.1001908#s5

b) *Published Peer Review*

Please note while forming your response, if your article is accepted, you may have the opportunity to make the peer review history publicly available. The record will include editor decision letters (with reviews) and your responses to reviewer comments. If eligible, we will contact you to opt in or out. Please see here for more details:

https://blogs.plos.org/plos/2019/05/plos-journals-now-open-for-published-peer-review/

c) *Blot and Gel Data Policy*

Please provide the original, uncropped and minimally adjusted images supporting all blot and gel results reported in an article's figures or Supporting Information files. We will require these files before a manuscript can be accepted so please prepare them now, if you have not already uploaded them. Please carefully read our guidelines for how to prepare and upload this data: https://journals.plos.org/plosbiology/s/figures#loc-blot-and-gel-reporting-requirements

d) *Protocols deposition*

To enhance the reproducibility of your results, we recommend that if applicable you deposit your laboratory protocols in protocols.io, where a protocol can be assigned its own identifier (DOI) such that it can be cited independently in the future. For instructions see: https://journals.plos.org/plosbiology/s/submission-guidelines#loc-materials-and-methods

Thank you again for your submission to our journal. We hope that our editorial process has been constructive thus far, and we welcome your feedback at any time. Please don't hesitate to contact us if you have any questions or comments.

Sincerely,

Ines

--

Ines Alvarez-Garcia, PhD

Senior Editor

PLOS Biology

ialvarez-garcia@plos.org

-----------------------------------------

Reviewers' comments

Rev. 1:

In this manuscript, Nguen and colleagues develop a ciliary PKA sensor by targeting the PKA phosphorylation site of VASP to cilia via Arl13b. They then use this reporter to examine how various perturbations to the Hedgehog pathway alter the phosphorylation of this reporter. The work is well presented with useful diagrams showing what aspect is being tested at each point. Overall, I do not have major concerns with the data. However, the discussion is pretty minimal and does not do a good job of explaining the work. I suggest that a version of the pathway diagram be added to the discussion with details of what was established in the work. In addition, the authors need to supply information about the antibodies used, and the characterization of critical antibodies. In particular, the sources and verification of phospho VAMP and phospho Smo antibodies are needed.

Rev. 2:

In this manuscript, Nguyen and colleagues investigated the molecular mechanism of Hedgehog signal transduction in the primary cilium, a crucial process during embryonic development with important implications in Hedgehog-driven cancers and other cilia-dependent disorders. More specifically, the authors investigated how the oncogene Smoothened (SMO) inhibits protein kinase A (PKA) activity in the primary cilium. To do so, they developed a ciliary PKA reporter that assesses the PKA signaling status in primary cilia after cell or tissue fixation. In the presented work, the authors confirm the previously reported mode of PKA inhibition by SMO through a protein kinase inhibitor motif in the C-terminal tail, which requires GRK2/3 phosphorylation. While the gist of this mechanism has been described by Walker et al. (in PLoS Biol, 2024), the authors provide additional mechanistic insight that will be of interest to the readership investigating Hedgehog signal transduction, primary cilia, and PKA signaling.

The main conclusions of the manuscript agree with the current literature and are therefore supported not only by the presented data but also previous work. Intriguingly, the authors gained additional insight in the regulation of PKA activity in primary cilia, as SMO appears to block PKA activity independent of G proteins. While this has been suggested in previous work (Walker et al., 2024) the supporting data was limited. Here, the authors provide additional supporting data for such a model, yet, the data is still not convincing, as the experimental setup does not justify the conclusion. However, based on previous publications the authors could design more adequate experiments to unambiguously confirm their hypothesis that PKA regulation in Hedgehog signaling may be independent of a putative G protein coupling of SMO.

While the developed PKA reporter will be a valuable tool for the community, it also has limitations, as it cannot follow the PKA activity in living cells and can only assess one point in time in the primary cilium. Therefore, to avoid confusion, I recommend the use of the word <reporter> instead of <sensor>, as the latter will be misleading for the readership studying signal transduction processes. Nevertheless, I want to re-iterate that this PKA reporter will be a powerful tool for the community, as it is compatible with fixation of specimens -in contrast to most biosensors.

The manuscript reports two main findings, 1) the development of a PKA activity reporter, and 2) SMO-mediated inactivation of PKA may be independent of G proteins. The second point requires additional work to warrant publication in its current form:

* The suggested model claims reduction of PKA activity by SMO when Hedgehog signaling is active. Therefore, one would assume that PKA activity is high when the pathway is inactive. However, the pVASP-based PKA reporter does not detect any PKA activity in unstimulated cells. How do the authors explain that they require a strong PKA activator, such as forskolin (FSK) to activate PKA in a seemingly unphysiological way in cell culture cilia? It is unclear whether FSK also needs to be added to see the in vivo differences in tissues (Fig. 2). Why this discrepancy? If the reporter lacks the sensitivity in cell culture to differentiate between non-stimulated (PKA active) and Hedgehog stimulated (PKA inactive) cilia in the absence of FSK, the resulting interpretations stand on a weak foundation. How do the authors explain that activation of SMO by SAG antagonizes the unphysiological FSK-mediated increase in PKA activity in cilia. The control experiment that this is SMO dependent (Fig 3G) is convincing, but it is unclear whether this is a cilium-specific effect. As FSK activates PKA in the entire cell (not only in cilia) SAG treatment may reverse the non-cilia PKA activity. The same might be true for inhibiting PKA activity by stimulating SSTR3. As these processes seem very difficult to dissect, the authors should at least provide alternative explanations for these findings, as they cannot rule out cilia-independent effects.

* Whether SMO is indeed a G-I-coupled GPCR appears to be a long-standing controversy in this field. One key experiment of this study (Fig.5) shows that the inhibition of the PKA activity by a cilia-localized SSTR3 receptor can be prevented by pertussis toxin (PTX), which blocks the G-I-mediated signaling cascade. In contrast, PTX treatment does not prevent blocking the PKA activity by SMO/SAG, which suggests that this effect is not mediated by G-I. However, the way how this experiment was performed does not allow drawing this conclusion. PTX works by blocking G-I, which itself blocks adenylate cyclases to lower cAMP production. For PTX to show any effect, requires an -independent- activation of the adenylate cyclases, usually by other stimulating G-S coupled GPCRs. One important adenylate cyclase stimulating GPCR in the Hedgehog signaling pathway is GPR161, which is removed from cilia shortly after Hedgehog induction (Mukhopadhyay, 2013). The experiment in Fig.5 was performed by adding SAG for 24 hours, followed by PTX treatment for 16 hours. Under these conditions the stimulating GPR161 was removed long before the PTX treatment started. Under these conditions, it cannot be expected that PTX increases cAMP levels in cilia as the only known stimulating GPCR is absent from cilia. Hence, on shorter timeframes or in more physiological concentration ranges (Hedgehog morphogens are highly concentration dependent in vivo), it cannot be ruled out that SMO may mediate its function via G-I coupling. One potential way to address this, would be a short-term SMO activation when increased levels of cAMP are present in cilia, which may be provided by FSK or more cilium-specific cAMP production, such as by optogenetics, which the authors have nicely done in previous studies (Truong et al., 2021).

To further strengthen the manuscript, I have the following suggestions:

* A central new finding of this study that PKA activity in cilia is modulated independently of G proteins suggests that it may be independent of cAMP levels. However, this contrasts with their previous, very prominent publication (Truong et al., 2021), in which they show that Hedgehog signaling can be modulated by locally modulating the cAMP levels. The authors should clarify this difference for the non-specialists.

* As the developed PKA reporter is at the heart of this manuscript, a more thorough description would be helpful for a better assessment by the reader. For example, the authors should provide lower magnification images in cell culture, such that the localization and specificity of the signals can be evaluated. Along the same line, the authors overstate findings obtained with their PKA reporter. For example, they conclude that they "produced a half-maximal increase in cilia PKA" activity. Such a specific statement can hardly be made from three concentrations investigated (Fig. S1D). Also, the authors did not determine any enzymatic activity of PKA, so to not confuse experts on molecular signaling, the statement in the discussion should be re-phrased (page28).

* Similarly, while I appreciate the experiment presented in Fig.S4 that only very few cilia with pSMO and pVASP signals could be found by the authors, the "mutually exclusiveness" criterion is not met. I recommend re-phrasing or providing additional data.

* The use of the PKA reporter to investigate different tissues in zebrafish embryos is a great achievement, however, the LUT chosen to display the results is misleading. The overlap of magenta and green should be white, yet, white is part of the magenta-based LUT displaying pVAST (displayed in Fig. 2), such that it is unclear how much of the overlap in the insets is indeed from an overlap or the high intensity of the pVASP signal.

* The authors nicely show that by converting the SMO C-tail from a pseudosubstrate to a substrate of PKA it ceases to block PKA activity. They further propose in the discussion that this should increase its dissociation rate, which seems like the most plausible mechanism. While not essential, the authors should consider generating such data to further confirm this proposed mechanism.

Minor points:

* Page2: G proteins that couple to GPCRs are not the "small" GTPases but "trimeric" GTPases by common nomenclature.

* Pages9/10: panel numbering of the figure seems off.

* Page10: The test for linear trend should be specified.

* Scale bars should represent micrometers not micromolar concentrations.

* Hoescht dye is misspelled.

* Page15: bottom, "induced induce" should be fixed.

* Page19: concentration of SAG for the experiments in Fig S4 should be provided.

* Page23: SMO-M2 mutation contains error in amino acid exchange.

Rev. 3:

Nguyen et al examines the relationship between SMO and PKA within primary cilia. Specifically, they are building upon their findings that the ciliary pool of PKA and not the cytoplasmic pool is specifically inhibits activation of HH signaling, and also extending recent findings, primarily from the Myers lab, that SMO primarily activates HH signaling at the cilium by binding to and inactivating PKA. The authors develop a PKA biosensor consisting of a PKA-specific substrate motif (from VASP) added to GFP and targeted to cilia by fusion to Arl13b. The phosphorylated motif is then recognized by a specific phospho-VASP antibody. They then demonstrate that this biosensor detects graded PKA activity in zebrafish somite development that corresponds to graded HH signaling in that tissue. They then use this biosensor to interrogate how PKA is regulated by SMO within cilia. I generally found this work to be well-performed experimentally and well presented. My main critique is that it represents only a modest advance in our mechanistic understanding of SHH pathway regulation in the cilium, relative to prior work (for example: Arveseth et al., 2021; Happ et al., 2022; Walker et al., 2024). The biosensor is and important tool that has enabled confirmation of prior findings with spatial resolution. This certainly has value, but may not be right for publication in PLoS Biology absent substantial additional findings that provide genuinely new mechanistic insights.

# Author comment: Author response to Decision Letter 2

**DOI:** 10.1371/journal.pbio.3003841.r003

13 Nov 2025

Attachment Submitted filename: Response to Reviewers Nguyen et al.pdf

# Peer review: Decision Letter 2

**DOI:** 10.1371/journal.pbio.3003841.r004

**Authors:** Ines Alvarez-Garcia

12 Jan 2026

Dear Dr Reiter,

Thank you for your patience while we considered your revised manuscript entitled "Smoothened and ciliary GPCRs regulate ciliary protein kinase A activity" for publication as a Research Article at PLOS Biology. Your revised study has been evaluated by the PLOS Biology editors, the Academic Editor and the three original reviewers.

The reviews are attached below. You will see that while the reviewers appreciate the improvements made in the revision, there are some issues remaining that would need to be addressed. Reviewer 1 mentions that GPR161 knockout didn’t decrease ciliary PKA activity as expected nor its overexpression increased the activity, and that the L465P mutant shows reduction in ciliary pVASP signalling, suggesting that it is a dominant negative. However, the reviewer thinks it is possible that it is not a null allele, and that experiments in cells where a GRP161 effect is observed should be repeated. Reviewer 2 also mentions that essential information on the GPR161-deficient cell line is missing along with information on the antibodies used in the new experiments, and raises similar concerns than the previous reviewer regarding the L465P mutant. Reviewer 3 is mostly satisfied, but notes some inconsistencies regarding the baseline levels of activity shown in several figures that should be explained, including Fig. 7 baseline reported activity in GPR161 mutant cells, and that Fig. S2 should be provided as it seems to be missing.

In light of the reviews and after consultation with the Academic Editor and the rest of the team, we would like to invite you to revise the work to thoroughly address the remaining issues raised by the reviewers.

Please note that we cannot make a decision about publication until we have seen the revised manuscript and your response to the reviewers' comments. Your revised manuscript is likely to be sent for further evaluation by all or a subset of the reviewers.

In addition to these revisions, you will need to complete some formatting changes, which you will receive in a follow up email. A member of our team will be in touch with a set of requests shortly.

We expect to receive your revised manuscript within 3 months. Please email us (plosbiology@plos.org) if you have any questions or concerns, or would like to request an extension.

At this stage, your manuscript remains formally under active consideration at our journal; please notify us by email if you do not intend to submit a revision so that we may withdraw it.

**IMPORTANT - SUBMITTING YOUR REVISION**

Your revisions should address the specific points made by each reviewer. Please submit the following files along with your revised manuscript:

1. A 'Response to Reviewers' file - this should detail your responses to the editorial requests, present a point-by-point response to all of the reviewers' comments, and indicate the changes made to the manuscript.

*NOTE: In your point-by-point response to the reviewers, please provide the full context of each review. Do not selectively quote paragraphs or sentences to reply to. The entire set of reviewer comments should be present in full and each specific point should be responded to individually, point by point.

You should also cite any additional relevant literature that has been published since the original submission and mention any additional citations in your response.

2. In addition to a clean copy of the manuscript, please also upload a 'track-changes' version of your manuscript that specifies the edits made. This should be uploaded as a "Revised Article with Changes Highlighted" file type.

3. Resubmission Checklist

When you are ready to resubmit your revised manuscript, please refer to this resubmission checklist: https://plos.io/Biology_Checklist

To submit a revised version of your manuscript, please go to https://www.editorialmanager.com/pbiology/ and log in as an Author. Click the link labelled 'Submissions Needing Revision' where you will find your submission record.

Thank you again for your submission to our journal. We hope that our editorial process has been constructive thus far, and we welcome your feedback at any time. Please don't hesitate to contact us if you have any questions or comments.

Sincerely,

Ines

--

Ines Alvarez-Garcia, PhD

Senior Editor

PLOS Biology

ialvarez-garcia@plos.org

------------------------------------

Reviewers' comments

Rev. 1:

In this revision, Nguyen and colleagues revised their previous work to describe a ciliary PKA reporter. My previous concerns focused on needed documentation of the antibodies and their characterization, and the failure of the discussion to put the work into the context of existing data. The discussion now does a better job of putting the work into context. The sources of the antibodies have been provided, but I see little in the way of discussion of what validation they were subjected to.

The big problem with the current submission is the new figure 7. The investigators knocked out GPR161, but the data were inconsistent with expectations: the loss of GPR161 did not alter (reduce) ciliary PKA activity as expected, nor did over-expression increase ciliary PKA activity. Further compounding the confusion, expression of an L465P mutant form of GPR161 in the KO background did have the expected reduction in ciliary pVASP signal. This would suggest a dominant negative activity, but the L465P construct failed to enrich PKA-R or PKA-C seen with wild type, making the idea of a dominant activity hard to justify. The materials and methods are vague about how the genomic DNA was analyzed to demonstrate a strong allele and the western is underwhelming in quality, so it is possible that this is not a null allele. Did this mutation show the other expected phenotypes for a GPR161 KO? Did you see increased Gli2 at the ciliary tip of unstimulated cells? Does Smo accumulate (I expect it would, based on review articles, but I was not able to find the original data to support this point)? As this figure now stands, the data seriously undermines the value of the reporter and the importance of the rest of the manuscript. Perhaps the key experiments need to be repeated in cells where a GPR161 effect is observed.

Rev. 2:

I very much appreciate the work that the authors put in the revised version of this manuscript. In the light of the newly presented data, expanding the discussion will certainly be valued by the readers. While I am also thankful for the thorough assessment of this work by the other reviewers, I do not agree with reviewer 3 that this work is "only a modest advance in our mechanistic understanding of SHH pathway regulation". Many different models of how PKA activity in primary cilia may be regulated in SHH signaling are being discussed. The presented data make a strong case that SMO-based inhibition of PKA activity can be independent of G protein-coupling, which is consistent with previous work by other groups. At the same time, the work shows that ciliary PKA activity can also be regulated by "conventional" GPCR signaling via G protein-coupling (see Figure 5), likely due to changes in ciliary cAMP, which is also consistent with previous work by several groups. Therefore, I do consider the work an important contribution to the mechanistic question of how PKA is activated in primary cilia and how this mediates SHH signaling.

Unfortunately, the authors fail to provide seemingly trivial but essential information. The authors now present additional experiments in a newly generated GPR161-deficient cell line, which is very elegant. However, the reader does not get sufficient information on this cell line. Because this cell line is so central to their conclusion and because it brings up a new, very puzzling result (see comments below), I must insist that the authors provide data that the cell line is indeed deficient in GPR161.

Additional points:

1. It is unclear why the authors do not include information on the antibodies they used for the new experiments, as this was one of the few requests raised by reviewer 1. Please include this critical information, also in future publications. It is essential information and you know it.

2. Figure 7: The changes in the localization of the PKA subunits are striking. However, the result on the PKA reporter in the L465P mutant is very puzzling. The authors provide a potential interpretation; however, a much simpler interpretation is that the putative GPR161-/- is not a full knockout, and hence the L465P might have a dominant-negative effect on PKA activation on wildtype GPR161. The authors must provide unambiguous evidence that the mutant is indeed lacking GPR161.

3. The new Figure 7 caption in its current form is not as informative as other captions. Understanding the figure requires reading the main text. Maybe this can be changed, i.e. by hinting at the L465P mutation.

4. The extended discussion will be very valuable to the readers of this article. The authors' insight into the AKAP function of GPR161 and how it regulates PKA activity by directing PKA holoenzymes to their sites of action is a very important result, but they should be acknowledge that they were not the first to propose that GPR161 functions mainly by localizing the PKA holoenzymes to primary cilia. They ought to cite either Bachmann et al., 2016 (PMID: 27357676) or Tschaikner et al., 2020 (PMID: 31947770) who proposed this, and May et al., 2021 (PMID: 33856408) who provided evidence that the ciliary localization of PKA is indeed regulated in response to SHH signaling.

Rev. 3:

My main concern with this manuscript during the initial review was that despite the utility of the PKA reporter, the paper represented an incremental advance from other recent papers on the mechanisms of SMO activation/PKA inhibition in the primary cilium. I feel that the additional experiments performed by the authors largely addressed this concern and added more mechanistic insight. A few points do remain to be addressed prior to publication, however:

Reviewer 2 made a point about the ability of the reporter to detect base-line levels of PKA activity absent FSK. While the authors address this point, there remain inconsistencies with respect to what baseline levels look like throughout the manuscript. For example, there is still an apparent discrepancy between what is shown in Fig 1- which is no detection of pVASP in the cilium, and Fig.3 which shows baseline localization that disappears upon stimulation with SAG. Lack of detection in cilia is also what is presented in Fig S1. Figure 7 discusses baseline reporter activity in WT and GPR161-/- cells, however here we see intense localization of the reporter in both of these cases, which seems to be inconsistent with Fig. 1. Were these cells treated with FSK? If so they should be labeled as such.

In Fig 6 panel B, I am assuming the SMO-WRR, SMO-GRL, and SMO-WRR-GRL were all treated with SAG. The labeling of the figures themselves should reflect this, otherwise it appears that these mutants behave like SMO-M2 and constitutively inhibit PKA activity.

Minor:

In Figure 6 the various SMO mutants are discussed in a different order in the text than how they appear in panels 6B, C, and D. Since this is a complex figure, keeping the order consistent will help the reader.

There is no Fig S2- goes from S1 to S3

# Author comment: Author response to Decision Letter 3

**DOI:** 10.1371/journal.pbio.3003841.r005

22 Apr 2026

Attachment Submitted filename: Nguyen et al Response to Reviewers 2.docx

# Editor report: Decision Letter 3

**DOI:** 10.1371/journal.pbio.3003841.r006

**Authors:** Ines Alvarez-Garcia

8 May 2026

Dear Dr Reiter,

Thank you for your patience while we considered your new revised manuscript entitled "Smoothened and ciliary GPCRs regulate ciliary protein kinase A activity" for publication as a Research Article at PLOS Biology. This revised version of your manuscript has been evaluated by the PLOS Biology editors and by the Academic Editor.

Based on our Academic Editor's assessment of your revision, we are likely to accept this manuscript for publication, provided you satisfactorily address the data and other policy-related requests stated below my signature.

In addition, we would like you to consider a suggestion to improve the title:

"Smoothened and ciliary GPCRs regulate ciliary protein kinase A activity to activate the hedgehog pathway"

As you address these items, please take this last chance to review your reference list to ensure that it is complete and correct. If you have cited papers that have been retracted, please include the rationale for doing so in the manuscript text, or remove these references and replace them with relevant current references. Any changes to the reference list should be mentioned in the cover letter that accompanies your revised manuscript.

In addition to these revisions, you may need to complete some formatting changes, which you will receive in a follow up email. A member of our team will be in touch with a set of requests shortly. If you do not receive a separate email within a few days, please assume that checks have been completed, and no additional changes are required.

We expect to receive your revised manuscript within two weeks.

To submit your revision, please go to https://www.editorialmanager.com/pbiology/ and log in as an Author. Click the link labelled 'Submissions Needing Revision' to find your submission record. Your revised submission must include the following:

- a cover letter that should detail your responses to any editorial requests, if applicable, and whether changes have been made to the reference list

- a Response to Reviewers file that provides a detailed response to the reviewers' comments (if applicable, if not applicable please do not delete your existing 'Response to Reviewers' file.)

- a track-changes file indicating any changes that you have made to the manuscript.

NOTE: If Supporting Information files are included with your article, note that these are not copyedited and will be published as they are submitted. Please ensure that these files are legible and of high quality (at least 300 dpi) in an easily accessible file format. For this reason, please be aware that any references listed in an SI file will not be indexed. For more information, see our Supporting Information guidelines:

https://journals.plos.org/plosbiology/s/supporting-information

*Published Peer Review History*

Please note that you may have the opportunity to make the peer review history publicly available. The record will include editor decision letters (with reviews) and your responses to reviewer comments. If eligible, we will contact you to opt in or out. Please see here for more details:

https://plos.org/published-peer-review-history/

*Press*

Should you, your institution's press office or the journal office choose to press release your paper, please ensure you have opted out of Early Article Posting on the submission form. We ask that you notify us as soon as possible if you or your institution is planning to press release the article.

*Protocols deposition*

To enhance the reproducibility of your results, we recommend that if applicable you deposit your laboratory protocols in protocols.io, where a protocol can be assigned its own identifier (DOI) such that it can be cited independently in the future. Additionally, PLOS ONE offers an option for publishing peer-reviewed Lab Protocol articles, which describe protocols hosted on protocols.io. Read more information on sharing protocols at https://plos.org/protocols?utm_medium=editorial-email&utm_source=authorletters&utm_campaign=protocols

Please do not hesitate to contact me should you have any questions.

Sincerely,

Ines

--

Ines Alvarez-Garcia, PhD

Senior Editor

PLOS Biology

ialvarez-garcia@plos.org

------------------------------------------------------------------------

ETHICS STATEMENT:

Thank you for providing the ethics statement. Please also include an approval number.

------------------------------------------------------------------------

DATA POLICY:

Thank you for providing the data underlying all the graphs shown in the figures. I have checked it and I have the following queries:

- Please let us know where the data underlying the graph in Fig. S2F is located or provide it if it is missing.

- The data underlying the graph in Fig S4C seem to be mislabeled as Fig. S5C. Please correct this.

- Please let us know where the data underlying the graph in Fig. S5 is located or provide it if it is missing.

- Please relabel the data files following format verbatim: S1 Data, S2 Data, etc. and save it using exactly the following convention: S1_Data.xlsx (using an underscore).

- Please also mention in all the corresponding figure legends (including the ones for the main figures) where the data can be found.

------------------------------------------------------------------------

SPECIES INDICATED IN THE ABSTRACT?

We note that you use zebrafish embryos in some of the experiments, but this is not mentioned in the abstract. Please mention this.

-------------------------------------------------------------------------

BLOT AND GEL REPORTING REQUIREMENTS:

We require the original, uncropped and minimally adjusted images supporting all blot and gel results reported in an article's figures or Supporting Information files. We will require these files before a manuscript can be accepted so please prepare and upload them now. Please carefully read our guidelines for how to prepare and upload this data: https://journals.plos.org/plosbiology/s/figures#loc-blot-and-gel-reporting-requirements

# Author comment: Author response to Decision Letter 4

**DOI:** 10.1371/journal.pbio.3003841.r007

13 May 2026

Attachment Submitted filename: Nguyen_et_al_Response_to_Reviewers_2_auresp_4.docx

# Editor report: Decision Letter 4

**DOI:** 10.1371/journal.pbio.3003841.r008

**Authors:** Ines Alvarez-Garcia

22 May 2026

Dear Dr Reiter,

Thank you for the submission of your revised Research Article entitled "Smoothened and ciliary GPCRs regulate ciliary protein kinase A activity involved in Hedgehog signal transduction" for publication in PLOS Biology. On behalf of my colleagues and the Academic Editor, Dagmar Wachten, I am delighted to let you know that we can in principle accept your manuscript for publication, provided you address any remaining formatting and reporting issues. These will be detailed in an email you should receive within 2-3 business days from our colleagues in the journal operations team; no action is required from you until then. Please note that we will not be able to formally accept your manuscript and schedule it for publication until you have completed any requested changes.

Please take a minute to log into Editorial Manager at http://www.editorialmanager.com/pbiology/, click the "Update My Information" link at the top of the page, and update your user information to ensure an efficient production process.

PRESS

We frequently collaborate with press offices. If your institution or institutions have a press office, please notify them about your upcoming paper at this point, to enable them to help maximise its impact. If the press office is planning to promote your findings, we would be grateful if they could coordinate with biologypress@plos.org. If you have previously opted in to the early version process, we ask that you notify us immediately of any press plans so that we may opt out on your behalf.

We also ask that you take this opportunity to read our Embargo Policy regarding the discussion, promotion and media coverage of work that is yet to be published by PLOS. As your manuscript is not yet published, it is bound by the conditions of our Embargo Policy. Please be aware that this policy is in place both to ensure that any press coverage of your article is fully substantiated and to provide a direct link between such coverage and the published work. For full details of our Embargo Policy, please visit http://www.plos.org/about/media-inquiries/embargo-policy/.

Many congratulations and thanks again for choosing PLOS Biology for publication and supporting Open Access publishing. We look forward to publishing your study.

Sincerely,

Ines

--

Ines Alvarez-Garcia, PhD

Senior Editor

PLOS Biology

ialvarez-garcia@plos.org
